# WHO Environmental Noise Guidelines for the European Region: A Systematic Review on Environmental Noise and Annoyance

**DOI:** 10.3390/ijerph14121539

**Published:** 2017-12-08

**Authors:** Rainer Guski, Dirk Schreckenberg, Rudolf Schuemer

**Affiliations:** 1 Department of Psychology, Ruhr-University Bochum, 44801 Bochum, Germany; 2 ZEUS GmbH, Zentrum für Angewandte Psychologie, Umwelt- und Sozialforschung, Sennbrink 46, 58093 Hagen, Germany; schreckenberg@zeusgmbh.de; 3 Independent Researcher, 58095 Hagen, Germany; ar-schuemer@t-online.de

**Keywords:** traffic noise, environment, annoyance, surveys, exposure-response, meta-analysis

## Abstract

*Background*: This paper describes a systematic review and meta-analyses on effects of environmental noise on annoyance. The noise sources include aircraft, road, and rail transportation noise as well as wind turbines and noise source combinations. *Objectives:* Update knowledge about effects of environmental noise on people living in the vicinity of noise sources. *Methods:* Eligible were published studies (2000–2014) providing comparable acoustical and social survey data including exposure-response functions between standard indicators of noise exposure and standard annoyance responses. The systematic literature search in 20 data bases resulted in 62 studies, of which 57 were used for quantitative meta-analyses. By means of questionnaires sent to the study authors, additional study data were obtained. Risk of bias was assessed by means of study characteristics for individual studies and by funnel plots to assess the risk of publication bias. *Main Results:* Tentative exposure-response relations for percent highly annoyed residents (%HA) in relation to noise levels for aircraft, road, rail, wind turbine and noise source combinations are presented as well as meta-analyses of correlations between noise levels and annoyance raw scores, and the OR for increase of %HA with increasing noise levels. Quality of evidence was assessed using the GRADE terminology. The evidence of exposure-response relations between noise levels and %HA is moderate (aircraft and railway) or low (road traffic and wind turbines). The evidence of correlations between noise levels and annoyance raw scores is high (aircraft and railway) or moderate (road traffic and wind turbines). The evidence of ORs representing the %HA increase by a certain noise level increase is moderate (aircraft noise), moderate/high (road and railway traffic), and low (wind turbines). *Strengths and Limitations:* The strength of the evidence is seen in the large total sample size encompassing the included studies (e.g., 18,947 participants in aircraft noise studies). Main limitations are due to the variance in the definition of noise levels and %HA. *Interpretation:* The increase of %HA in newer studies of aircraft, road and railway noise at comparable *L*_den_ levels of earlier studies point to the necessity of adjusting noise limit recommendations. *Funding:* The review was funded by WHO Europe.

## 1. Introduction

Environmental noise annoyance is often observed in the context of environmental noise due to transportation via aircraft, road, and rail, and partially in industrial neighborhoods. When asked to name the main noise effect, 50.6% of 68 international noise experts answered “annoyance” [1]—which points to the high prevalence of annoyance as a noise effect. In terms of ‘burden of disease’, WHO Europe [2] estimated annoyance to be the second major health effect of environmental noise after sleep disturbance. Several reviews showed exposure-response relations (ERR) between noise levels of aircraft, road, and railway noise and the percentage of highly annoyed residents (%HA) (e.g., [3,4,5]). As part of the WHO work on developing new noise guidelines [6], we performed a systematic review of the literature and meta-analyses of survey data on annoyance related to transportation noise.

The main objectives of the systematic review were to assess the strength of association between exposure to environmental noise and long-term noise annoyance, based on field research reported between the years 2000 and 2014, to quantify the increase of annoyance with an incremental increase in noise exposure, and to present an exposure-response relation for each noise source. The main noise sources considered are aircraft, road traffic, railway traffic, and wind turbines. In addition, effects of noise source combinations and stationary sources are considered. This paper presents an overview and the main results; detailed analyses are given in the Supplementary Materials. As a consequence of the analyses, updates of ERRs between %HA and noise levels are suggested with respect to aircraft and railway noise.

## 2. Materials and Methods

### 2.1. Defining the Effect Variable: Annoyance

Environmental noise annoyance as observed in surveys is a retrospective judgment, comprising past experiences with a noise source over a certain time period. The noise annoyance response usually contains three elements:

(1) an often repeated disturbance due to noise (repeated disturbance of intended activities, e.g., communicating with other persons, listening to music or watching TV, reading, working, sleeping), and often combined with behavioral responses in order to minimize disturbances;
(2) an emotional/attitudinal response (anger about the exposure and negative evaluation of the noise source); and
(3) a cognitive response (e.g., the distressful insight that one cannot do much against this unwanted situation).

This multi-faceted response is seen by many researchers as a stress-reaction (e.g., [7]) involving an environmental threat and individual physiological, emotional, cognitive, and behavioral responses which can partly be remembered and be integrated into a verbal long-term annoyance response. The noise annoyance response considered here is related to long-term exposure, i.e., related to residents who live in a more or less noisy area for at least one year and answer noise annoyance questions related to a long period of time. The participants of the included studies were selected according to specified procedures and answered at least one standardized noise annoyance question.

Today, the two annoyance questions and the response scales used in field studies often correspond to the recommendation of International Committee for the Biological Effects of Noise (ICBEN) [8] and International Standards Association (ISO) [9]. We used this recommendation as a standard. This standard relates to three elements: (1) the position of the question within the questionnaire (“early”); (2) the annoyance question (asking for a certain location and integrating over a certain period of time) and (3) the type of response scale (5-point verbal with equal steps, and/or 11-point numerical). For instance, the numerical form of the ICBEN question is “Next is a zero to ten opinion scale for how much (source) noise bothers, disturbs or annoys you when you are here at home. If you are not at all annoyed choose zero, if you are extremely annoyed choose ten, if you are somewhere in between, choose a number between zero and ten. Thinking about the last (12 months or so), what number from zero to ten best shows how much you are bothered, disturbed, or annoyed by (source) noise?”

It should be noted that the location “here at home” mentioned in the standard annoyance question, does not distinguish between “inside” and “outside” of the home. This was intentionally left open to the interpretation of the study participants. There are indications that study participants—at least in Western studies—include the outside part of their homes when answering the unspecified standard question (see Supplementary Materials S34).

A special effect variable is the percentage of “highly annoyed” study participants. “Highly Annoyed” (HA) are respondents who choose a high position on the annoyance response scale. The exact cut-off point between “highly annoyed” and “not highly annoyed” varies somewhat from study to study, but there is de facto a standard, established by Schultz [10]: respondents using about 72% of the response scale (i.e., the upper 28%) are called “highly annoyed”. Nowadays, there is a new standard: ICBEN [8] recommends using the upper two steps of the verbal 5-point response scale for defining “highly annoyed” people (i.e., the upper 40% of the response scale). However, only a minority of our studies used this option.

### 2.2. Search and Selection of Studies

For a start, we performed a literature search in 20 databases, including MEDLINE/PubMed, Scopus (includes Embase), PsycInfo, Psyndex Plus (covering psychological journals, and grey literature), Web of Science, ScienceDirect, DIMDI (a German medical information system, covering journals and grey literature), Bielefeld Academic Search Engine (BASE), EBSCO, Ingenta-Connect, Google Scholar, and Springer-Link. Additionally, we searched the publication lists of Rijksinstituut voor Volksgezondheid en Milieuhygiene (RIVM, The Netherlands), Department for Environment, Food and Rural Affairs (DEFRA, UK), and ICBEN. As far as possible, we used the search string “((noise AND annoyance) AND ((exposure-response) OR (dose-response)))” and restricted the search to the publication years 2000–2014 according to the review protocol defined by WHO Europe. At the end, we got about 1700 hits, of which 112 were non-redundant and described observational studies on residents exposed to noise from at least one of the five noise sources: road traffic, rail traffic, air traffic, industrial sites, and wind turbines.

*Selection criteria for the formal meta-analysis*: We included only studies which fulfilled the following criteria:

(1) Study type: cross-sectional or longitudinal surveys, using an explicit protocol for selecting respondents.
(2) Participants: Studies including members of the general population (mainly residents of noise-exposed areas).
(3) Exposure type: Long-term outside noise levels which are either expressed in *L*_Aeq,24h_, *L*_dn_, *L*_den_ or its components (*L*_day_, *L*_evening_, *L*_night_ and the duration in hours of night—see Supplementary Materials S37 for definitions of these terms), or can be easily converted from similar acoustic variables AND:
  - The level is based on a reliable calculation procedure, using the actual traffic volume, composition, and speed per 24 h per road/railway/airport as input, or the type and sound power of an industrial installation, OR
  - is based on measurements for a minimum of one week by qualified staff, and adjusted for data under point (a) as well as meteorological conditions when necessary.
(4) Outcome measure: The base of the outcome measure is the individual annoyance response made during a standardized survey. The annoyance question and the response format either follow the recommendations given by ICBEN [8] and/or ISO TS 156666 [9] directly, or are very close to them. The paper (or the authors on request) gives at least one original table, formula, or graph which can be used for an ERR.
(5) Confounders: Papers containing a potential second risk factor besides noise (e.g., vibrations in case of railway noise close to the tracks) are included and got special remarks in the list of included papers.
(6) Language: Papers in English, French, Dutch, and German were included as long as they met the selection criteria. These languages were selected according to the language understanding of the present authors.

We excluded all papers on interventions; these are handled by another WHO review group [11].

### 2.3. Data Extraction

Preparing for data extraction, we judged the eligibility of each of the 112 papers from the title, abstract and method section, using essentially comparability criteria, e.g., method of participant selection, method of noise calculation, and/or method of annoyance measurement (see list of included/excluded papers in Supplementary Materials S3). Papers were included when they reported data from a field study about residential noise (data from 1996 to 2014), used more than three noise exposure level categories which were comparable with standard noise levels (like *L*_den_, *L*_day_, *L*_eq,24h_), used a standard participant selection method (e.g., a stratification according to noise levels, and random selection within the strata), used a comparable annoyance response assessment (like the ICBEN/ISO questions and scales [8,9]), and reported an exposure-response relation for a comparable HA-definition. Data from infrastructural change studies and military airports were excluded. This judgment was done independently by the three authors of the present paper. In case of conflict, we discussed it and found a common conclusion. As a result, we got a list of 34 annoyance papers containing 62 individual studies that could possibly be used in the evidence review.

We produced an extensive description of each of the 62 studies, containing data about study details like type of study, main type of noise source, survey date, location, rationale for site selection, noise metrics, distribution of levels in the survey, number of respondents, response rate, non-response analysis, annoyance scale, main outcome measure, definition of highly annoyed, additional co-determinant variables, statistical approach, and type of ERR.

We then asked the study authors to provide additional study data, e.g., on survey dates, noise exposure level range for several descriptors, Pearson correlations between noise levels and annoyance raw scores, four exposure descriptors, HA-definition, percent HA at 50 and 60 dB for four exposure descriptors, type of calculated exposure-response function (ERF), equation parameters, and R^2^ for bivariate and multivariate regression models. The questionnaires can be found in the Supplementary Materials S1. The completed questionnaires sent back by the study authors are the (aggregated) study data used in exposure-response estimations and meta-analyses. As expected, not all of the authors were able to answer all of our questions; hence, many data are missing.

After receiving the authors’ responses, a formal rating of the quality of each study was done on the basis of the ICBEN recommendations for study-reporting (Fields et al. [12]), supplemented by the present authors with respect to rating the quality of the social survey as well as the quality of acoustic sound level estimations. The study quality rating relates to the uncertainties connected with the data for exposure and response within a study. As such, the study quality rating is no rating of the study risk of bias. Even studies with higher degrees of uncertainty (i.e., lower quality) may report unbiased exposure-response relations. However, we expect results from higher quality studies to be more reliable (reproducible) than results from lower quality studies. The study quality comprises six sections: Overall survey design (six items), Social survey sample (three items), Social survey data collection (three items), Nominal acoustical conditions (six items), Basic exposure-response analysis (one item), and Explanatory variable analysis (one item). All quality ratings are based on the published reports, i.e., the ratings can only include aspects reported. The rating table is shown in Supplementary Materials S2.

At the end, we included 62 studies in the qualitative analysis, of which 57 were included in a quantitative synthesis (meta-analysis). Two reviewers independently extracted data from the publications and respective questionnaires of the individual studies included. Meta-analyses were performed by means of the CMA V3 program [13].

**Figure 1:**
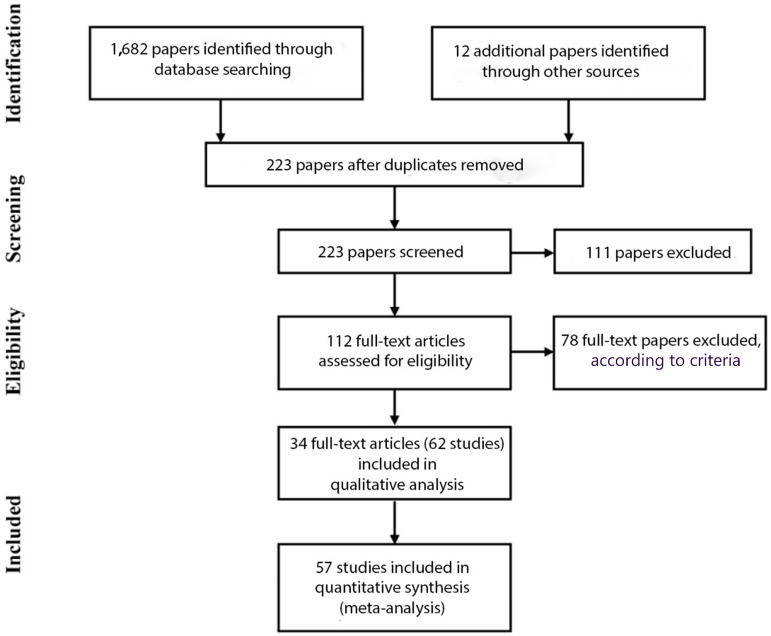
Flow-chart of the study selection process (following the PRISMA flow-chart, Moher et al. [14]). Selection criteria are explained in Supplementary Materials S3.

A flow-chart of the study selection process is given in Figure 1, according to the requirements of the Preferred Reporting Items for Systematic reviews and Meta-Analyses (PRISMA)-statement (see Moher et al. [14]).

### 2.4. Effect-Size Measures

Besides providing exposure-response curves for the relation between noise levels (in terms of *L*_den_) and percent HA, we consider three types of effect-size measures, which are listed here in the order in which they appear in the questionnaire sent to the authors:

- *Pearson correlations for L_Aeq_ vs. annoyance raw scores.* Correlation coefficients using the (partially restricted) range of reported noise exposure levels for a specific source in 1 dB steps and the full range of the noise annoyance scale for each study are taken as effect-size measures for our formal meta-analysis. The noise level ranges vary between noise sources and studies (see Tables 1, 3 and 5. Although correlations as such do not indicate a causal relationship, it is plausible that a statistical association between (external) transportation noise levels—related to the past 12 months—and annoyance judgments due to transportation noise—related to the same 12 months—indicates an effect of noise on annoyance—and not the other way round. Correlation coefficients between noise levels and annoyance raw scores contain the most complete information about the effect of environmental noise levels on noise annoyance, as observed in surveys, although they are rarely used for health impact assessments. Pearson correlations restrict this information to linear relations, but it has been shown in the past that raw annoyance scale variables usually show a linear relation to *L*_Aeq_-variables, and the inclusion of non-linear terms does not improve the correlation—at least with such large samples as used here. Here, mainly *L*_Aeq,24h_ or *L*_den_ are used as exposure variables, and raw scores on the 11-point numeric or 5-point verbal ICBEN scale as response variables.
- *Increase of percent HA with increase of L_Aeq_ levels, based on observed data.* The %HA-increase was determined in terms of odds ratios (OR). The OR denotes the ratio of two odds. Here, each of these odds represents the proportion of highly annoyed participants divided by the proportion of those not highly annoyed at a certain exposure level. Thus, the OR referring to a %HA-increase by an increase of exposure levels is defined as the ratio of the odds for each of the two exposure levels. The increase of the event rate (such as %HA) for an increase of 5 or 10 dB *L*_Aeq_ is sometimes used in noise effect reports [15,16,17], because this metric indicates the increase of a severe noise effect (%HA) with a certain increase of noise exposure. Although the use of this metric is quite popular in political contexts, we should keep in mind that the size of the “increase effect” is heavily dependent on three parameters: (a) the definition of “highly annoyed” (see above); (b) the noise level range considered for the dB-difference, together with the form of the exposure-response relation; and (c) the data source (e.g., observed data vs. calculated ERF). Provided that the standard definition of HA is used, it is often seen that the %HA-curves show a nonlinear relation to equivalent noise levels, taking the form of a “J” (as is the case in the well-known %HA curves in Miedema and Oudshoorn [4]). In such cases, it can be expected that the %HA-difference between two noise levels at the lower end of the exposure scale is lower than the respective difference at medium or higher noise levels. There may be other forms of ERRs and especially in case of a small range of noise levels which are not comparable between studies, the 10-dB-difference approach may produce misleading results. With respect to (c) we should keep in mind that calculated ERFs for %HA use a wide range of noise levels and data from the whole set of respondents together with assumptions about the S-form of the ERR, and %HA can be calculated in small steps on the decibel scale. On the other hand, observed data for certain noise levels (e.g., 50 and 60 dB) often imply using small groups of respondents (often N < 100) around these levels (e.g., from 47.5 to 52.4 dB in the case of a “50 dB group”), leading to “real” subsamples of small size. We use the OR based on the %HA at 50 and 60 dB for transportation noise and the OR based on the %HA at 42.5 and 47.5 dB for low level noise source types, e.g., wind turbines.
- *Increase of %HA with increase of L_Aeq_ levels, based on modelled data.* We used equation/parameter values (e.g., B or exp(B) for logistic regression) for the model, specified for type of ERR (e.g., linear regression, logistic regression: binary, polynomial fit, etc.). Such parameters partially use the full information contained in the ERR and partly restricted information (e.g., in the case of logistic regression). Generally, a modelled ERF may overcome restrictions due to small samples in certain noise level groups. They can be used to calculate predicted annoyance values for specified noise levels as well as for determining the change in annoyance between specified noise level differences. This change could be expressed as an OR. The slope parameter B from logistic regressions represents a logarithmized OR (ln(OR)) and can be used to estimate the effect of a 10 dB difference; these estimated ORs can be compared to the ORs based on the observed %HA at each of the two levels. Furthermore, the regression equations from the studies can be used for estimating aggregated ERR.

### 2.5. Publication Bias Assessment

We mainly used funnel plots in order to assess the risk of publication bias, i.e., the plot of the distribution of effect sizes in relation to a scale indicating the precision of the effect estimation is taken in order to detect a possible publication bias at review level. In addition, information about a possible selection bias (e.g., restricted age range) is taken as information about a risk of a bias at study level. Both methods are taken into account in the assessment of the quality of evidence for the respective exposure-response curves and effect-size measures. However, due to a lack of data, we were not able to account for socio-economic or cultural factors, such as average socio-economic status (SES), education, income, house ownership, or percentage of immigrants in the study samples. It is conceivable—and sometimes empirically shown—that low SES is associated with higher noise levels, and there are indications that house ownership is slightly associated with increased noise annoyance. However, we did not have social data that are comparable between studies, and, therefore, the results shown here are not “adjusted” for social data.

Risk of bias assessments are subsequently used to inform the GRADE assessment in Table S1, Tables S3–S5 (aircraft noise), Tables S6–S9 (road noise), Tables S10–S13 (railway noise), Tables S15, S16, S18 (wind turbine noise) and S20 (combined sources noise).

### 2.6. Quality of Evidence Assessment

The quality of evidence was assessed using the GRADE approach. The GRADE system consists of four levels of quality of evidence: high, moderate, low, and very low. High quality evidence implies: Further research is very unlikely to change our confidence in the estimate of effect. Moderate quality evidence implies: Further research is likely to have an important impact on our confidence in the estimate of effect and may change the estimate. Low quality implies: Further research is very likely to have an important impact on our confidence in the estimate of effect and is likely to change the estimate, Very low quality implies: Any estimate of effect is very uncertain. Further details are given in the Supplementary Materials.

## 3. Results

This section is subdivided into different noise sources: aircraft, road, and rail traffic, wind turbine noise as well as noise source combinations. Within each subsection, a short description of studies selected, exposure-response curves (including a GRADE table), meta-analyses of the three effect-size measures (including GRADE tables), and a short summary is given.

Note: There are instances of extreme heterogeneity in the meta-analyses (e.g., I^2^ > 80), which means that a large part of the total variance is due to “true” variance between studies. Performing a meta-analysis at all may be questioned in such cases. However, provided that predefined eligibility criteria are sound and the data are correct, a meta-analysis may be performed, and the causes of heterogeneity should be explored (see respective sections in the Supplementary Materials).

### 3.1. Aircraft Noise Effects on Annoyance

For many years now, aircraft noise is associated with the highest degree of long-term noise annoyance, as observed in systematic surveys comparing the degree of average or high annoyance between transportation noise sources at comparable long-term noise levels [3,4,18].

#### 3.1.1. Studies Selected

Data from 15 aircraft noise annoyance surveys around national and international airports were collected from publications and the completed authors’ questionnaires. The surveys took place from 2001 to 2014, encompassed a total of 18,947 respondents, and a noise level range from 11 to 74 dB *L*_Aeq,24h_, resp. from 12 to 78 dB *L*_den_ and 11 to 77 dB *L*_dn_, i.e., from small airports with 34 regular flights per day to large international airports with more than 1200 movements per day. Most of the statistical analyses presented in Section 3.1 either used cut-off values for *L*_den_ and *L*_dn_ ≥ 40 dB, or implied exposure levels which exceeded *a-priori* these cut-off values. Fortunately, most of the selected studies use the annoyance question and scales according to ICBEN/ISO as well as several standard noise level descriptors. Except for one study, all studies define “Highly Annoyed” (HA) by the upper 27% of the response scale, i.e., HA ≥ 73% (see Table 1). It should be noted that the six HYENA-studies (indicated as “Babisch 2009”) included residents aged 45–70 years only, while most of the other studies start at 18 years of age. Given the often reported non-linear relation between age and annoyance (e.g., [19], p. 187: “peaking around 45 years”) is true for the HYENA-studies, too, we can assume a certain bias towards higher annoyance. However, we did not have data to test this assumption. Two Japanese studies show a restricted level range (12 dB in terms of *L*_den_). The list of papers included/excluded is shown in Supplementary Materials S3.

#### 3.1.2. Aircraft Noise Effects (1): ERRs in the Full Dataset

The ERR estimated in this review describes the statistical relation between a number of exposure classes (here: noise levels in decibel) and the estimated response (here: %HA) at each exposure class. In this review, the %HA in each of the different exposure classes is based on modelled ERFs provided by study authors, weighted according to the number of participants in the respective study, and subjected to linear or quadratic regression as a curve-fitting tool.

For 12 of the 15 aircraft noise studies, ERF of the relation between *L*_den_ and modelled %HA were available, aggregating data from 17,094 study participants. In all of the studies, HA is defined by a cut-off at ≥73% of the response scale. Different regression models were used in the respective studies. A binary logistic regression was performed in the majority of the studies; in some studies, a polynomial regression model was used, and one study used a multilevel grouped regression. We calculated the percentages for 5-dB steps from 40 to 75 dB in the level range that was actually used in the respective study. For eight of the studies noise levels starting from 40 dB *L*_den_ were used, and four other ones starting from 45/50/55 dB, respectively. For three of the studies noise levels up to 75 dB *L*_den_ were used, for five studies up to 70 dB, for three up to 65 dB, and for one up to 60 dB *L*_den_. The calculation of the (predicted) percentages at the different exposure levels used the parameters of the regression function reported by the authors.

**Figure 2:**
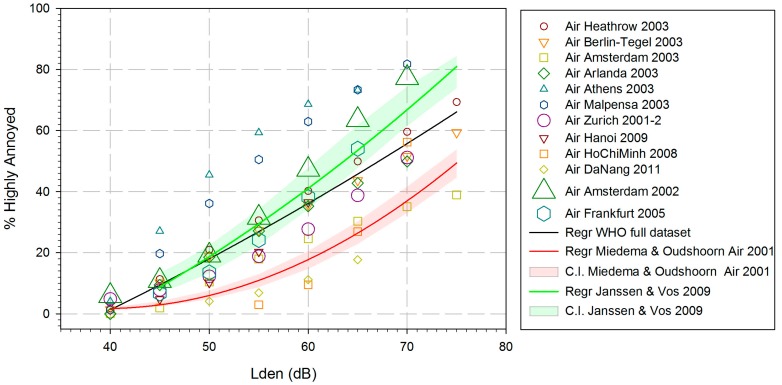
Scatterplot and quadratic regression of the relation between *L*_den_ and the calculated %HA for 12 aircraft noise studies, together with ERFs by Miedema and Oudshoorn ([4], red), and Janssen and Vos ([20], green). Notes: (1) The size of the data points corresponds to the number of participants in the respective study (size = SQRT(N)/10). (2) If two results from different studies fall on the same data point, the last point plotted may mask the former one. (3) The black curve is derived from aggregated secondary data, while the red and green curves are derived from individual data. In addition, the mathematical models used for establishing the three functions differ.

The corresponding estimated data points for each of the 12 studies (called WHO full dataset) are plotted in Figure 2, together with the estimated ERR for the aggregated data (black line). It should be noted that the “estimated data points” do not represent independent empirical observations, but rather predicted values estimated from the regression equation for each of the studies. This means (inter alia) that all estimates of the %HA values for the different exposure levels from the same study are not independent from one another. 

The estimated ERR depicted in Figure 2 is based on a quadratic regression between *L*_den_ and the aggregated (secondary) WHO data set, weighted according to the square root of the respective study sample size. The quadratic regression fits best to the data, in comparison to linear or cubic regressions. The coefficient of determination (R²) of the aggregated data set is R^2^ = 0.700 (squared fit)—which seems large, but we have to keep in mind that the data are not original survey data—they are aggregated secondary data derived from calculations. In order to get an impression of different ERFs, the functions from Miedema and Oudshoorn [4] and Janssen and Vos [20], together with their respective confidence intervals, are depicted in Figure 2 as well, although, different data sources and calculation procedures were used. The equation for estimated %HA by *L*_den_ noise levels of aircraft noise in the WHO dataset is:

Estimated %HA = −50.9693 + 1.0168 × *L*_den_ + 0.0072 × *L*_den_^2^.

A visual inspection of data curves does not provide clear information about the similarity or distinctness of curves. A better alternative would be to compare the confidence intervals or—at least—tolerance intervals. However, this is impossible to do with individual observed data on the one hand and aggregated calculated data on the other. The reader will already have noticed that we do provide confidence intervals for both the Miedema and Oudshoorn [4] and the Janssen and Vos [20] ERFs, but none for the WHO dataset. The latter is technically possible but not applicable, because the calculation of a confidence interval usually assumes a certain measuring model with a certain distribution of errors in combination with independent observations. None of these assumptions is met here; therefore, we do not provide confidence intervals for aggregated data.

#### 3.1.3. Grading the Quality of Evidence for the ERR of %HA by Aircraft Noise

The GRADE system [21,22] classifies the quality of evidence in one of four levels—high, moderate, low, and very low. WHO has adapted the classification criteria for start levels and cross-sectional studies—typical for annoyance surveys—start as “high quality”. The confidence in the evidence with respect to ERRs between aircraft noise levels and the percentage of high aircraft noise annoyance may be decreased for several reasons, including study limitations, inconsistency of results, indirectness of evidence, and publication bias. With respect to the latter, it should be remembered that six of the studies in the WHO data set include residents aged 45–70 years only, which might have contributed to an increase of annoyance. In sum, we are moderately confident in the evidence regarding the ERRs between aircraft noise levels and percentage of high aircraft noise annoyance and like to assign the grade “moderate quality”. For detailed information, see Supplementary Materials S4.

#### 3.1.4. Aircraft Noise Effects (2): ERRs in High-Rate and Low-Rate Airport Change Situations

It is sometimes stated that recent airport noise annoyance surveys are often done in the context of abrupt change, i.e., before and/or after a step change of airport traffic (e.g., by implementing a new runway, changing flight routes, and/or an abrupt increase of the number of aircraft movements). Janssen and Guski [23] call airports “low-rate change airports” as long as there is no indication of a sustained abrupt change of aircraft movements, or the published intention of the airport to change the number of movements within three years before and after the study. “An abrupt change is defined here as a significant deviation in the trend of aircraft movements from the trend typical for the airport. If the typical trend is disrupted significantly and permanent, we call this a ‘high-rate change airport’. We also classify this airport in the latter category if there has been public discussion about operational plans within [three] years before and after the study” ([23], p. 8). This definition might be somewhat arbitrary and far from perfect. For instance, it does not cover changes in the composition of aircraft fleets or tragic aircraft crashes. Irrespective of its shortcomings, the definition has been used already by [24], and we explored the influence of high-rate airport changes on our dataset with respect to this definition as far as possible.

**Figure 3:**
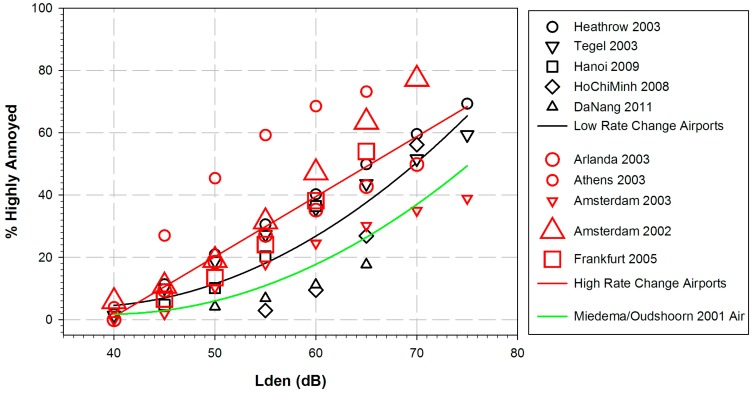
Scatterplot and regression lines of the relation between *L*_den_ and the calculated %HA for five “high-rate change” (red curve) and five “low-rate change” (black curve) airport noise studies, together with exposure-response function by Miedema and Oudshoorn ([4], green curve). Notes: (1) The size of the data points corresponds to the number of participants in the respective study (size = SQRT(N)/10). (2) If two results from different studies fall on the same data point, the last point plotted masks the former one. (3) The red and black curves are derived from aggregated secondary data, while the green curve is derived from individual data. In addition, the mathematical models used for establishing the three functions differ.

From the 12 studies of the WHO aircraft dataset, we consider five airports as “low-rate change”: Heathrow 2003, Tegel 2003, Hanoi 2009, Ho Chi Minh 2008, and Da Nang 2011. Another five airports are considered to be “high-rate change” airports (see Figure 3): Arlanda 2003, Athens 2003, Amsterdam 2002, Amsterdam 2003, and Frankfurt 2005. Arlanda airport opened a new runway in 2003 (the survey was administered 2003–2005), Athens airport opened 2001 (two years before the start of the survey), Amsterdam-Schiphol opened a new runway in 2003 (one of the studies was administered in 2002, the other from 2003 to 2005). At Frankfurt Airport, there is an ongoing public discussion (including citizen protest movements) for decades, and the survey conducted in 2005 preceded an official airport expansion statement from the county administration by two years. We refrained from classifying both the Zurich 2001 and the Milano-Malpensa 2003–2005 studies in terms of “change”, because there are no clear indications related to our high-rate-change definition. There have been public discussions about flight routes (Zurich) or expansion plans (Malpensa), and these have been ongoing for several years before and after the surveys—which indicates a tendency in the direction of “change”, but does not fit exactly to the definition of a “high-rate change”.

The results of the separation of %HA values for “low-rate” and “high-rate change” airports are shown in Figure 3. Although there is a certain overlap of the “change” and “no-change” data points in this figure, it is evident that the majority of the “change” points are higher than the majority of the “no-change” points. The two regression lines (black for “low-rate change” and red for “high-rate change”) overlap only at the highest and lowest noise levels. The “high-rate change” regression line shows a good linear fit (R^2^ = 0.74) to the weighted data points, while the “low-rate change” regression line shows a good quadratic fit (R^2^ = 0.77). The “high-rate change” regression overlaps considerably with the curve published by Janssen and Vos [20] (not shown here), except for the highest noise levels. It has been noted in the preceding paragraph that four of the seven airports in the studies by Janssen and Vos [20] may be seen as “high-rate change” airports. The black “low-rate change” regression line of the WHO dataset seems to be somewhat closer to the Miedema and Oudshoorn [4] curve. However, the gap between the two curves may be seen as an indication of the so-called annoyance trend, i.e., an increase of the percentage of highly annoyed persons in more recent studies as compared to earlier studies, even in low-rate change situations. This is confirmed by results of a more recent study published after the period of publication years considered in this review (2000–2014). In the German Noise-Related Annoyance, Cognition, and Health (NORAH) study [25] the results concerning aircraft noise annoyance suggest that the percentage of highly annoyed people are not only elevated at ”high-rate change” airports (Frankfurt, Berlin-Brandenburg), but also in the vicinity of ”low-rate change” airports (Cologne/Bonn, Stuttgart) compared to the curve published by Miedema and Oudshoorn [4].

#### 3.1.5. Aircraft Noise Effects (3): Correlations between Noise Levels and Annoyance Raw Scores

##### Meta-Analyses in the Full Dataset

All authors of the 15 studies reported Pearson’s r for the relation between individual *L*_Aeq,24h_, *L*_den_ or *L*_dn_ and individual aircraft noise annoyance. Correlation coefficients are our primary effect-size variables. These data were entered (together with the respective sample size) into the meta-analysis program, ordered by author and study. A random effect model was chosen; it assumes that the true effect may vary from study to study. By contrast, the fixed-effect model is based on the assumption that there is one true effect size which underlies all of the studies in the analysis, and that all differences in observed effects are due to sampling error ([26], p. 61).

**Figure 4:**
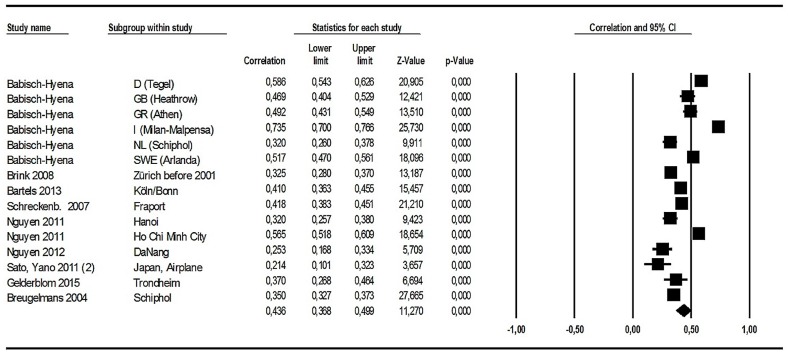
Meta-analysis of 15 aircraft noise studies, based on correlations between individual *L*_den_ or *L*_dn_ and annoyance raw scores, Random Effects Model. The right part of the graph contains a forest plot of the correlations and their respective 95% confidence intervals. The figures of the last row indicate the summary estimates.

Figure 4 contains results from a meta-analysis on correlations between *L*_den_ or *L*_dn_ and annoyance raw scores for 15 aircraft noise annoyance studies. The effect size (correlation in Figure 4) for each study is graphically represented at the right side (“Forest plot”) by means of a square, with the location of the square representing both the direction and magnitude of the effect. The size of each square reflects the weight assigned to the study when the summary effect is computed. The weight is primarily determined by study/sample size. In Figure 4, the sizes of the squares are very similar, because the study samples are of similar size and a random effect model was used for analysis, resulting in smaller weighting differences between studies. In addition, the effect size for each study is bounded by a 95% confidence interval, reflecting the precision with which the effect size has been estimated in that study. At the bottom of the schematic part of the graph, the position and size of the diamond represents the summary effect. At first glance, it is nothing more than the weighted mean of the individual effects. But the assumptions and formulas used to assign the weights (providing the meaning of the summary effect) differ between the so-called “fixed” and “random” effect models. “Under the fixed-effect model, we assume that all studies in the analysis share the same true effect size, and the summary effect is our estimate of this common effect size. Under the random-effects model, we assume that the true effect size varies from study to study, and the summary effect is our estimate of the mean of the distribution of effect sizes” ([26], p. 6). We tend to assume the latter and prefer the random effects model.

As expected, all aircraft noise effects (expressed as correlation coefficients) are positive and statistically highly significant in a test against the null (*p* < 0.01). However, there is a considerable spread (r from 0.21 to 0.74 (see column “Correlation”); lowest value = 0.101, highest value = 0.766 (see columns “Lower Limit” and “Upper Limit”)). The group of HYENA studies shows somewhat larger correlations, compared to the other studies in this analysis, but in view of the potential confounders associated with the HYENA group (age range, two change airports, face-to-face-interviews, annoyance question related to daytime), it seems impossible to explore this aspect thoroughly (some of these aspects are analyzed in Supplementary Materials S7). The summary correlation (last row in Figure 4) is r = 0.436 (95% CI = 0.368–0.499). Two of the studies included show rather low correlation coefficients (r = 0.253 (Da Nang) and r = 0.214 (Japan Airplanes)), which might be due to the restricted range of noise levels (12 dB *L*_den_). There is a proposal to correct low correlation in case of restricted range by means of an estimation procedure which uses—among others—the standard deviation of the noise levels for the restricted as well as unrestricted ranges [27]. Unfortunately, we did not get such data, and we had to take the correlations as they were provided by the study authors.

Additional material related to correlations between aircraft noise levels and annoyance raw scores are given in the Supplementary Materials S5 and S6. S5 compares correlation coefficients between annoyance raw scores and two different descriptors for the 24 h noise exposure. This comparison does not show important differences. S6 shows a funnel plot analysis as a means of detecting a possible publication bias. This plot may be interpreted as showing no bias in the direction expected (large effect sizes at low precision): we have large effects in middle-sized studies, e.g., Milano-Malpensa, Athens, Berlin-Tegel, and Ho Chi Minh City. Supplementary Materials S7 explores the heterogeneity of the correlations between annoyance raw scores and aircraft noise levels.

##### Grading the Quality of Evidence for the Correlation between Aircraft Noise Levels and Annoyance

Our confidence in the quality of evidence with respect to correlations between aircraft noise levels and aircraft noise annoyance is relatively high, and we assign the grade “high quality”. For more information, see Supplementary Materials S8.

#### 3.1.6. Aircraft Noise Effects (4): ORs Referring to the %HA Increase per 10 dB Level Increase

If we concentrate on %HA, we get a somewhat different view as compared to annoyance raw scores: Respondents are called “HA”, when they choose a high position on the annoyance response scale (see Section 2.4). Since the relation between *L*_Aeq_-type noise levels and observed %HA often is non-linear (mostly taking the form of a “J”), the noise level used for comparisons may be critical. With regard to transportation noise, the percentage of HA in the area from 50 to 60 dB *L*_Aeq_ (during daytime, or 24 h) is often used for a discussion of health effects (e.g., [2,28,29]). The difference between %HA at 50 dB vs. 60 dB *L*_den_ or *L*_Aeq,24h_ can be used as an indicator of severe noise annoyance effects at moderate to high noise levels. First, we will present an analysis of the %HA difference based on original data. Then, another analysis based on modelled data, using the full range of noise levels, will be presented. In both types of analyses, the change in the %HA between different exposure levels will be determined as an OR.

##### Meta-Analysis Based on Original Grouped Data

Eleven of the 15 aircraft noise studies in our sample provided original grouped data for %HA at 50 dB and 60 dB *L*_den_*.* One of these studies was excluded due to 0% HA in one of the levels. A meta-analysis of the ORs in studies including one or more zero entries would require a correction for the zero rates. In this case, the results of the analyses would heavily depend on the choice of the correction procedure. Different procedures (e.g., [30,31]) produce divergent results.

The percentages reported in the resulting ten studies were entered into the meta-analysis program as “event rates” and converted to ORs—after dividing by 100, and supplemented by the n of cases at each of the level classes. That is, the program calculates the relation between HA-rate at 60 dB *L*_den_ and 50 dB *L*_den_ and provides an output for ORs.

Generally, the OR is calculated as the ratio of the odds in the two exposure classes. The odds are calculated as the ratio of the rate of highly annoyed in an exposure class and the rate of not highly annoyed in the same class. Here, an OR represents the odds that a certain outcome (to be highly annoyed) will occur, given a certain exposure level (60 dB) as compared to the odds of the same outcome, given a certain lower exposure (50 dB). To give an example: OR = 3 means that the odds or chance to be highly annoyed is three times higher in the upper exposure class (e.g., 60 dB) compared to the corresponding odds in the lower exposure class (e.g., 50 dB).

**Figure 5:**
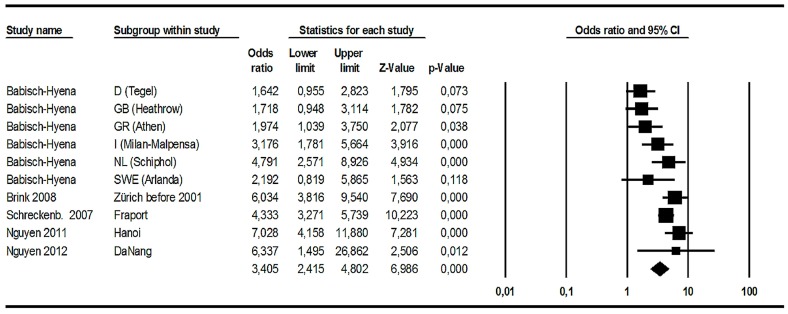
ORs and 95% confidence intervals for the OR referring to a %HA increase by a 10 dB increase (from 50 to 60 dB *L*_den_) aircraft noise. The right part of the graph contains a forest plot of the ORs and their respective 95% confidence intervals. The figures of the last row indicate the summary estimates.

Compared to Figure 4, Figure 5 gives a somewhat different view on the relation between long-term noise levels and annoyance judgments by residents: On the one hand, it can be stated that all ten ORs are above 1.0 and the summary ratio (last row) is 3.4 and highly statistically significant (*p* < 0.01). The size of the summary OR shows that there is a strong aircraft noise effect—which is in line with the analysis based on correlations between noise levels and annoyance raw scores. On the other hand, five of the ORs are greater than 1 but not statistically significant and show a relatively broad confidence interval. This is specifically true for the Arlanda/Brömma study (from HYENA) and the Da Nang study (Nguyen, 2012), see Table 2. It should be noted that the Arlanda/Brömma study contained less than hundred respondents exposed to 50 and 60 dB, respectively, and in the Da Nang study a relatively low correlation between exposure and response was observed.

Testing for the heterogeneity of the ORs in our sample of aircraft studies, we found statistically highly significant *Q*-values (*Q* = 32.589, df = 9, *p* < 0.001, I^2^ = 72.383), which means that a large part of the total variance is due to “true” variance between studies and their respective locations and situations (e.g., rate of change, see Section 3.1.4). Further information about the meta-analysis of the ORs referring to the increase of %HA with increase of noise levels can be found in Supplementary Materials S9 (funnel plot) and S10 (exploring the between-study heterogeneity of ORs in original grouped data).

##### Meta-Analysis Based on Modelled Data

Only four aircraft noise annoyance studies provided parameters of a logistic regression of the ERR. We used the slope parameter to estimate the OR for a 10 dB difference of exposure. The meta-analysis of these estimates resulted in a summary OR = 4.778 (95% CI = 2.272–10.048; *p* < 0.001) which is statistically highly significant. Further details can be found in Supplementary Materials S11.

##### Grading the Evidence Based on ORs Representing the %HA Increase by a 10 dB *L*_den_-Increase of Aircraft Noise

Our confidence in the results regarding the direction of the OR referring to the increase of %HA is high (“high quality”), but limited with respect to the magnitude of the OR (“moderate quality”). For more information, see Supplementary Materials S12.

#### 3.1.7. The Influence of Co-Determinants in Aircraft Noise Studies

It is well known that there are several individual variables which influence the personal aircraft noise annoyance, like noise sensitivity and the coping capacity with respect to noise effects [27,32,33,34,35]. Variables like these often are called “moderator variables” in the noise literature, and they refer to within-study factors. In addition, there are other potential co-determinants, which may influence either the degree of noise annoyance at given noise levels (e.g., the rate of change at an airport), and/or the magnitude of the effect-size indicators considered here (e.g., a restricted range of noise levels in a certain study may decrease the correlation between exposure and annoyance). In this paper, we consider different study factors and restrict the meaning of the term “moderator” to the presence of such interactions where the size of the exposure-annoyance effect differs between the levels of a third variable or where the strength of the exposure-response relationship (ERR) depends on the level of a third variable. “Third variables” considered here are study characteristics such as study quality rating (see Supplementary Materials S2), survey type, noise level range, response rate, and rate of airport change. With respect to study characteristics, it has been shown that at least the “airport change situation” is associated with the level of residential annoyance (see Section 3.1.4 above). However, we found no evidence that the factor “change” has a statistically significant moderating effect on the strength of the ERR in the different studies (cf. Supplementary Materials S7). Further information is given in Supplementary Materials S10 and S13.

#### 3.1.8. Summary of the Analyses Related to Aircraft Noise Effects on Annoyance

Data from 15 aircraft noise annoyance surveys around national and international airports were used for several formal meta-analyses. They encompass a total of 18,947 respondents. All studies used the annoyance question and scales according to ICBEN/ISO as well as several standard noise level descriptors. Except for one study, all studies defined HA by the upper 27% of the response scale. The meta-analysis based on correlations between noise levels and annoyance raw scores used 15 studies and produced a statistically highly significant summary correlation (r = 0.436; 95% CI = 0.368–0.499; *p* < 0.001). This summary correlation shows that about 19% of the variance of aircraft noise annoyance raw scores is accounted for by the variance of *L*_den_ or *L*_dn_. The meta-analysis based on the OR for the increase of %HA per 10 dB increase of noise levels used ten studies with observed data for the level difference between 50 and 60 dB *L*_den_ and resulted in a statistically highly significant OR (OR = 3.405; 95% CI = 2.415–4.802; *p* < 0.001). However, a considerable variation of the ORs could be observed between studies. A corresponding meta-analysis of the OR referring to the increase of %HA per 10 dB level difference based on modelled data used only four studies and the summary OR was higher (OR = 4.778; 95% CI = 2.272–10.048; *p* < 0.001). However, the heterogeneity test was statistically highly significant as well, and there is considerable variance between studies. If we take the two meta-analyses on ORs together, the chance to be highly annoyed by aircraft noise is roughly between three to five times higher when the noise level increases by 10 dB.

A tentative ERF for the relation between *L*_den_ and %HA is shown, using equations from 12 studies and aggregating data from 17,094 study participants. The estimated ERR is based on a quadratic regression between *L*_den_ and the aggregated (secondary) WHO data set, weighted according to the square root of the respective study sample size. The resulting curve runs considerably higher than the curve presented by Miedema and Oudshoorn [4] for aircraft noise annoyance, especially at levels above 50 dB. A distinction between “high-rate change” and “low-rate change” airports results in two different exposure-response curves. Both curves show higher levels of %HA as compared to the Miedema/Oudshoorn [4] curve at comparable noise levels. The curve relating to “high-rate change” airports runs at almost the same level as the curve published by Janssen and Vos [20], while the curve relating to “low-rate change” airports runs somewhat lower.

Taken at face value, the mean percentages of residents highly annoyed by aircraft noise at certain noise levels in the 12 studies are higher than the percentages reported by Miedema and Oudshoorn [4]. Similar observations have been made by van Kempen and van Kamp [36] and Janssen and Vos [20], who partially used the same surveys as we did. The notion of an “aircraft annoyance trend” over time has been discussed in several publications, and numerous statements have been proposed in favor of a trend (e.g., Janssen et al., [37]), or against it (e.g., [24]). The latter maintain that there is no general aircraft noise annoyance trend, and that an increase of aircraft noise annoyance is connected with studies conducted in the context of (anticipated or completed) “high-rate change” airports only. Data of our present review support the idea of a slight general aircraft noise annoyance trend even at low-rate change airports and a considerably higher increase of aircraft noise annoyance at high-rate change airports.

### 3.2. Road Traffic Noise Effects on Annoyance

We included nine publications providing data from 26 studies of road traffic noise annoyance, ranging from a small-scale study in a small French town to a large study in Hong-Kong. The total set includes 34,211 respondents and noise level ranges from 10 to 82 dB *L*_Aeq,24h_, resp. from 16 to 83 dB *L*_den_ and 16 to 86 dB *L*_dn_ The level range data from the Alpine studies differed between different study reports and are not given in Table 3. Most of the statistical analyses presented in Chapter 3.2 either used cut-off values for *L*_den_ and *L*_dn_ ≥ 40 dB, or implied exposure levels which exceeded *a-priori* these cut-off values. Nineteen studies used the annoyance question and scales according to ICBEN, and seven studies used questions similar to the ICBEN-standard together with a 4-point verbal scale. Sixteen of the studies used a cut-off at 73% of the response scale in order to define HA, seven studies used a cut-off at 75%, and three studies used a cut-off at 60%. Several standard noise level descriptors were used; *L*_den_ was the most often included descriptor. Table 3 shows an abbreviated list of study data on road noise annoyance.

Three characteristics of the included road traffic noise studies should be noted:

(1) Some of the Asian studies show a restricted range of road traffic noise levels. We tested the hypothesis that a restricted level range will decrease correlations between noise levels and annoyance raw scores, but could not find a statistically significant difference between “high-range” and “low-range” level studies.
(2) The full data set includes five studies from Alpine valleys in Austria. With respect to acoustics, valleys are different from flat areas due to the so-called amphitheater effect, i.e., the propagation of sound to the valley slopes, including back-and-forth reflections of sounds produced in the valley. In the past, it has been shown that annoyance responses are usually higher in Alpine areas than in non-Alpine areas at similar levels of *L*_Aeq_ [38]. In addition, three of the five Alpine studies used ≥60% of the scale as a criterion for being highly annoyed (see Table 3), and some of the Alpine research sites are subject to long lasting discussions about heavy transalpine road and rail traffic due to the European integration. Especially with respect to road traffic, a large increase of goods traffic has been reported [38]. All of these factors may have contributed to increased annoyance at comparable exposure levels.
(3) The full data set includes the large Hong Kong study as well as nine additional studies from Asia, where many participants are living in air conditioned homes. This co-determinant factor may have contributed to a lower degree of annoyance, compared to the other studies included.

#### 3.2.1. Road Traffic Noise Effects (1): ERRs

##### Data Analysis for ERRs

For 17 of the 26 road traffic noise studies exposure-response equations of the relation between *L*_den_ and modelled %HA were available. In one case the exposure variable was related to *L*_Aeq,24h_. We transformed these values with the formula given in [39]: *L*_den_ = *L*_Aeq,24h_ + 2.6414 dB. In seven other cases, *L_dn_* data were provided and corrected to *L*_den_ = *L*_dn_ + 0.4847. For one study no equation could be obtained. In total, data from 34,112 study participants were used for estimating a common ERR. We calculated the percentages for 5-dB steps from 40 to 80 dB within the empirical range of levels used in the respective study. The range of noise levels for useable %HA data was not homogeneous between studies: for two studies, observed noise levels started at 40 dB and ended at 80 dB; for three studies, noise levels ranged between 40 and 70 dB, for another three 50–70 dB, and the other studies either started and/or ended at other decibel levels. Most of the studies from Vietnam and Thailand had a restricted level range from 65 to 80 dB and had very few respondents at lower noise levels within this range. In addition, the equations for the six HYENA and the Hong Kong studies used quadratic polynomials which may lead to an artificial increase of the estimated %HA below 45 dB. In answering our questionnaire, Wolfgang Babisch pointed out that he set the cut-off at 45 dB when analyzing the HYENA road data. Therefore, the 40 dB %HA data from the six HYENA and the Hong Kong studies were discarded.

**Figure 6:**
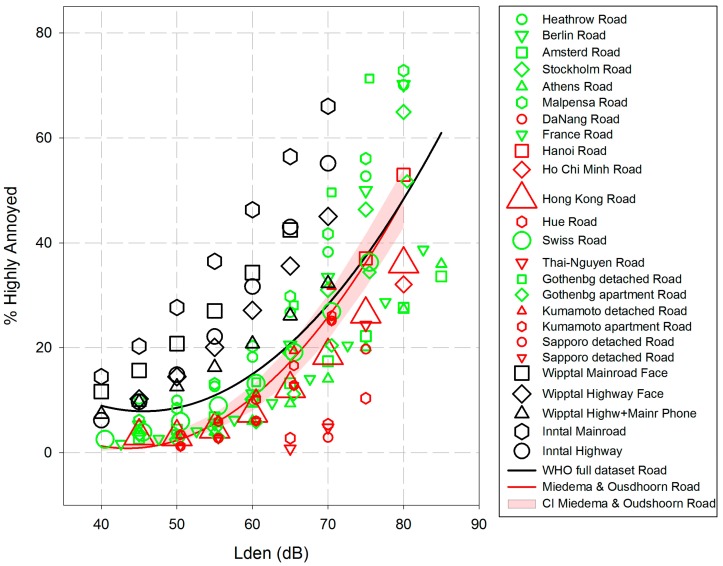
Scatterplot and quadratic regression of the relation between *L*_den_ and the calculated %HA for 25 road traffic noise studies (black line), together with the exposure-response function by Miedema and Oudshoorn [4] (red line). Notes 6: (1) Black symbols refer to valley studies, red symbols refer to Asian studies, and green symbols refer to European no-valley studies. (2) The size of the data points corresponds to the number of participants in the respective study (size = SQRT(N)/10). (3) If two results from different studies fall on the same data point, the last point plotted may mask the former one. (4) The black curve is derived from aggregated secondary data, while the red one is derived from individual data.

The corresponding data points for each of the 25 studies (called WHO full dataset) are plotted in Figure 6, together with the estimated ERR for the aggregated data (black line). The ERR shown below is based on a quadratic regression between *L*_den_ and the aggregated (secondary) WHO full dataset, weighted according to the square root of the respective study sample size. The variance explained by regression of the aggregated data set is R^2^ = 0.546 (squared fit)—which is similar to the regression observed in the aggregated aircraft noise dataset. We still have to keep in mind that the data are not original; they are secondary data derived from calculations. For comparison, the ERF from Miedema and Oudshoorn [4] for road traffic noise is also shown in Figure 6 (together with the respective CI).

It will be noted that the %HA at the lowest noise level (40 dB *L*_den_ in Figure 6) is somewhat higher than at the next higher level (45 dB). This is due to the five Alpine studies; which are the only studies left that provide %HA at 40 dB.

Figure 6 points to the extreme variation of average %HA in the level range 40–70 dB *L*_den_, e.g., from 0.78 to 56.41 %HA at 65 dB. The former is estimated for a relatively small study in Thai-Nguyen, the latter for a larger study at Inntal (Austria, main roads) showing the highest percentages of highly annoyed residents all over the range from 40–70 dB *L*_den_. It should also be mentioned that the second highest percentages are results of another Alpine study (Wipptal, main roads). In contrast, some of the Vietnamese studies show very low percentages of HA at levels above 65 dB *L*_den_. However, the aggregated regression line is almost dominated by the very large Hong Kong study (n = 10,077).

The equation for estimated %HA by *L*_den_ levels of road traffic noise in the full data set is:

Estimated %HA = 78.9270 − 3.1162 × *L*_den_ + 0.0342 × *L*_den_^2^.

**Figure 7:**
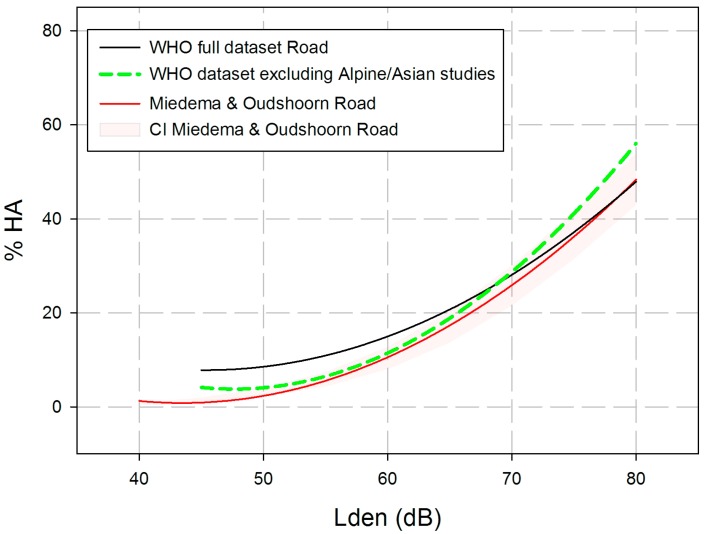
Quadratic regressions of the relation between *L*_den_ and the calculated %HA for 25 road traffic noise studies, (“full WHO data set”, black) vs. 10 studies (dashed green, same data set excluding the Alpine and Asian studies). For comparison, the exposure-response function by Miedema and Oudshoorn ([4], road) is shown (red), together with the respective confidence interval. Note: The black and green curves are derived from aggregated secondary data, while the red curve is derived from individual data.

In comparing the exposure-response estimation for road traffic noise from our full aggregated dataset with the Miedema/Oudshoorn curve [4], it seems evident that the %HA in the WHO dataset are somewhat higher, especially at exposure levels from 40 to 65 dB *L*_den_. On the other hand, there is a large variation of %HA in our dataset. The comparability of the Alpine studies with studies from more or less flat landscapes, as well as the comparability of studies with and without air-conditioned homes may be questioned. Therefore, we computed an additional ERF for the WHO Road dataset excluding the five Alpine studies and the 10 Asian studies. All of the 10 remaining studies took place in European flat terrains, used the ICBEN-type of annoyance question, as well as a HA-criterion ≥73% or ≥75% of the response scale. The results of a simple quadratic regression analysis including 10 of the 25 road traffic noise studies (excluding the Alpine and Asian studies) are displayed in Figure 7. It is evident that the position and slope of the ERR changes considerably, if we exclude the Alpine and Asian studies from the dataset. The new curve runs somewhat closer to the old Miedema and Oudshoorn [4] curve for road traffic noise at levels between 45 to 65 dB Lden. However, the %HA increase considerable above 70 dB.

The equation for estimating %HA by *L*_den_ levels of road traffic noise in the data set excluding the Alpine and Asian studies is:

Estimated %HA = 116.4304 – 4.7342 × *L*_den_ + 0.0497 × *L*_den_^2^.

##### Grading the Quality of Evidence for the ERR of %HA by Road Traffic Noise in the Full WHO Dataset

In view of the extreme variation of average %HA in the full level range, we are not very confident in the evidence with respect to the ERR between road traffic noise levels and %HA by road traffic noise, and we assign the grade “low quality”. For details, see Supplementary Materials S14.

#### 3.2.2. Road Traffic Noise Effects (2): Correlations between Noise Levels and Annoyance Raw Scores

##### Meta-Analysis in the Dataset

Since most of the studies on road traffic noise annoyance analyzed here provide correlations between annoyance raw scores and *L*_den_, we take *L*_den_ as the general acoustic descriptor for the analysis of correlations between the noise load due to road traffic and annoyance raw scores, even in the cases of the Brink 2013, and the Sato et al. studies (which provide correlations with *L*_dn_). This decision was backed up by sensitivity tests resulting in statistically insignificant and very small effects of the acoustic descriptor (*L*_den_ or *L*_dn_) on the respective effect-size estimations. In addition, sensitivity tests did not show any statistically significant effect of the length of the annoyance scale (4-point/5-point/11-point; see Supplementary Materials S15) on the effect-size estimation.

**Figure 8:**
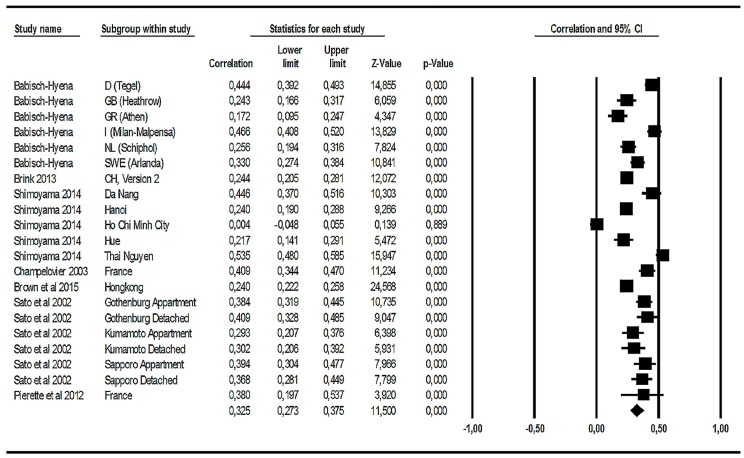
Meta-analysis of 21 studies using Pearson correlations between *L*_den_ or *L*_dn_ and road traffic noise annoyance raw scores. The right part of the graph contains a forest plot of the correlations and their respective 95% confidence intervals. The figures of the last row indicate the summary estimates.

We subjected all 21 available correlation coefficients (together with the respective n) to a meta-analysis. The results are shown in Figure 8. The four Alpine studies are not included, because no correlation coefficients were reported.

Twenty of the 21 road traffic noise annoyance-related correlations with *L*_den_ or *L*_dn_ are positive and statistically highly significant (*p* < 0.001). The summary correlation is 0.325 with a 95% confidence interval ranging from 0.273 to 0.375 (see last row in Figure 8). In sum, this shows a reliable effect of noise levels on road traffic noise annoyance. However, there are two aspects regarding the correlations worth looking at in more detail. First, there is a zero correlation in the Ho Chi Minh sample. Second, the confidence interval for the second French study (labeled ‘Pierrette et al.’ in Figure 8) is relatively large. With respect to the latter, it is probable that the small sample is the main reason for the large confidence interval. With respect to the zero correlation in the Ho Chi Minh study, a possible explanation might be a considerable restriction in the range of noise levels: just 6 dB between the maximum and minimum *L*_den_ levels. Range restriction can be a general problem when comparing correlations [40]. As stated earlier, a correction for range restriction was not feasible due to lack of standard deviations. Information about the between-study heterogeneity of correlations between noise levels and annoyance raw scores can be found in Supplementary Materials S15.

##### Grading the Evidence Based on Correlations between Road Traffic Noise Levels and Annoyance Raw Scores

We are moderately confident in the evidence concerning correlations between road traffic noise levels and road traffic noise annoyance raw scores, and like to assign the grade “moderate quality” (see Supplementary Materials S16).

#### 3.2.3. Road Traffic Noise Effects (3): ORs Referring to the %HA Increase per 10 dB Level Increase

##### Meta-Analysis Based on Observed Data

Twelve of the 26 road traffic noise studies provided observed data for the %HA at 50 and 60 dB or 55 and 65 dB *L*_den_ or *L*_dn_ (see Table 4).

**Figure 9:**
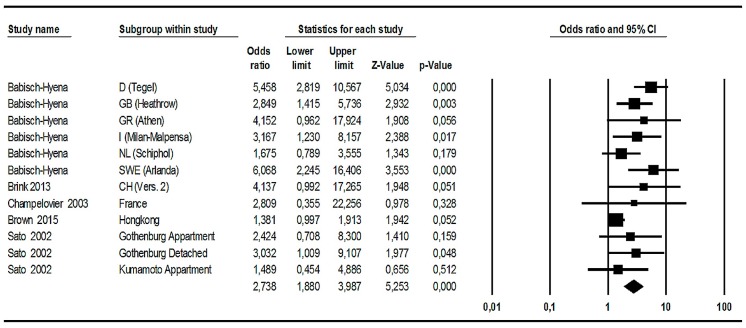
ORs and 95% confidence intervals for the observed “highly annoyed” increase by 10 dB increase (from 50 to 60 dB or 55 to 65 dB *L*_den_ or *L*_dn_) road traffic noise. The right part of the graph contains a forest plot of the ORs and their respective 95% confidence intervals. The figures of the last row indicate the summary estimates.

Four of the studies provided *L*_dn_-based data; all others used *L*_den_. Some studies provided data for the difference between 60 and 70 dB or 60 and 80 dB (see Table 4)—these differences were considered to be not comparable with the 50/60 difference. Hence, these studies were excluded from the meta-analysis on observed data, but they were included in the meta-analysis on modelled data—as far as they provided sufficient information. Data from 12 studies were used in order to calculate ORs in the next meta-analysis (see Figure 9). The percentages were (after dividing by 100 and supplemented by the n of cases at each of the level classes) entered into the meta-analysis program as “event rates” and converted to ORs. That is, the program calculates the ratio of the HA-odds at 50 and 60 dB *L*_den_ and provides an output for the OR (see Section 3.1.6 for a short explanation of OR).

It turned out that, in sum, there is an OR referring to the increase of %HA per 10 dB level increase, which is greater than 1 and statistically highly significant (summary OR = 2.738, 95% CI = 1.880–3.987; *p* < 0.001). This summary OR is somewhat lower than the comparable OR for aircraft noise. On the other hand, the dispersion of ORs for road traffic noise annoyance is much larger than that for aircraft noise annoyance: it ranges from about 1.4 (Hong Kong) to about 6.1 (Arlanda), and the lower confidence interval limits of seven studies are below 1.0—this indicates that the “true” OR in half of the studies included may not indicate an increase in %HA. In addition, it should be noted that there are only three statistically highly significant ORs (*p* < 0.01) in the analysis. Nevertheless, there are two additional studies revealing statistically significant ORs (*p* < 0.05).

So far, the global result of this 12-study meta-analysis on ORs referring to the observed increase of road traffic noise annoyance per 10 dB increase from 50 to 60 or 55 to 65 dB *L*_den_ or *L*_dn_ clearly indicates that there is a statistically highly significant effect on the increase of %HA in general. At the same time, the analysis shows considerable differences between studies—both with respect to the size of the increase effect (some very large, some very small effects) and the size of the confidence interval (great variation even within studies). There are seven studies with statistically non-significant results at *p* < 0.05. We found several indications for heterogeneity: The *Q*-test on heterogeneity is statistically significant (*Q* = 22.999; df = 11; *p* = 0.018), and I^2^ = 52.172, indicating that more than 50% of the total variance is due to “true” variance between studies, and this gives rise to the question of potential effect moderators.

There is additional information in the Supplementary Materials S17 and S18 (S17: Funnel plot of OR referring to the increase of %HA with increasing road traffic noise levels, and S18: Exploring the between-study heterogeneity of ORs in original grouped data).

##### Meta-Analysis Based on Modelled Data

Nineteen of the 26 road traffic noise studies provided parameters of a logistic regression of the ERR, and their slope parameters were used to estimate the OR for a 10 dB difference of exposure. The summary effect of the 10 dB level increase from modelled data is somewhat greater (OR = 3.033; 95% CI = 2.592–3.549; *p* < 0.001) than we have seen in the foregoing analysis based on observed data. Additional information is shown in the Supplementary Materials S19–S21.

##### Grading the Evidence of ORs Representing the %HA-Increase per 10 dB Level Increase of Road Traffic Noise

We are rather confident that there is evidence for an increase of %HA with an increase of road traffic noise level. However, the magnitude of the effect shows a large variation between studies in the case of original (grouped) data and less variation in the case of modelled data. Thus, the quality of evidence is moderate in the case of original data and high in the case of modelled data. More information can be found in Supplementary Materials S22.

##### The Influence of Co-Determinants in Road Traffic Noise Studies

The scientific literature shows evidence of two co-determinants influencing the road traffic noise annoyance: (a) availability of a quiet façade and (b) motorway vs. urban road (see Supplementary Materials S23). The annoyance level may differ between different studies depending on the proportion of respondents with/without a quiet façade or on the proportion of respondents living near motorways or urban roads, respectively.

#### 3.2.4. Summary of the Analyses Related to Road Traffic Noise Effects on Annoyance

Data from 26 studies of road traffic noise annoyance (including 34,211 respondents) were used for several meta-analyses and two tentative ERRs. Twenty-one studies were included in a correlational analysis between noise levels and annoyance raw scores, resulting in a statistically highly significant summary correlation between annoyance raw scores and *L*_den_ or *L*_dn_ (r = 0.325; *p* < 0.001). This summary correlation shows that about 11% of the variance of road traffic noise annoyance raw scores is accounted for by the variance of *L*_den_ or *L*_dn_. Twelve studies provided observed data for the %HA-increase at 50 and 60 dB or 55 and 65 dB *L*_den_ or *L*_dn_. It turned out that there is an OR referring to the %HA-increase per 10 dB level increase which is greater than 1 and statistically highly significant (summary OR = 2.738, 95% CI = 1.880–3.987; *p* < 0.001). The slope parameters of a logistic regression of the ERR were available for 19 road traffic noise annoyance studies. This parameter was used in order to estimate the OR for the %HA-increase per 10 dB increase of exposure. The summary effect of the 10 dB level increase from modelled data is somewhat greater (OR = 3.033; 95% CI = 2.592–3.549; *p* < 0.001) than the one obtained from observed data. If we take the two latter analyses together, we can state that the odds or chance to be highly annoyed is about three times higher when the road traffic noise level increases by 10 dB. The two funnel plots (for observed and for modelled data) both point to a certain publication bias in the direction of overestimation of the reported effects. There are statistically highly significant effects of the 10 dB increase, but the size of this increase may be overestimated in the studies analyzed here. Two tentative ERRs are presented for road traffic noise annoyance: a set of 25 studies including Alpine and Asian studies, and a set of 10 studies excluding them. The estimated ERRs between %HA and *L*_den_ are based on a quadratic regression between *L*_den_ and the aggregated (secondary) WHO data set, weighted according to the square root of the study sample size. The curve including the Alpine and Asian studies shows higher %HA at levels between 45 and 60 dB *L*_den_, while the curve excluding the Alpine and Asian studies is located mainly within the confidence interval of the Miedema/Oudshoorn [4] curve for road traffic noise annoyance—except for noise levels above 70 dB *L*_den_.

### 3.3. Railway Noise Effects on Annoyance

The eight publications included in the railway noise annoyance analysis contain descriptions of a total of 11 individual studies, performed from 1997 to 2010, with sample sizes from about 520 to 2000 (a total of 12,477 respondents), and annual noise levels from 24 to 86 dB *L*_eq,24h_, resp. 30 to 93 dB *L*_den_ and 30 to 84 dB *L*_dn_. The level range data from the Alpine studies differed between different study reports and are not given in Table 5. For most of the statistical analyses presented in Chapter 3.3 noise levels for *L*_Aeq,24h_ and *L*_den_ ≥ 40 dB were available. Ten studies used the ICBEN/ISO annoyance question together with the standardized 5-point verbal and/or 11-point answer scales. Six of the studies defined HA by ≥60% of the response scale, the remaining five studies defined HA ≥ 73% of the response scale (see Table 5).

Two of the studies are part of “combination studies” (either two noise sources, or noise and vibration), but only the latter is included here, because it has been shown that vibrations are a concomitant phenomenon of railway noise in some residential areas. Two of the studies involved high speed trains, the remaining studies involved conventional passenger and freight trains. We included the three Alpine railway noise studies as well as the Rhine valley study in spite of the acoustic differences between valleys and flat terrain and in spite of the long lasting public discussions about railway noise in these areas, and we included the two different definitions of HA in the same dataset, because we found the number of studies in subsets (e.g., four valley studies vs. 7 non-valley studies) too small in order to get reliable results.

#### 3.3.1. Railway Noise Effects (1): ERRs

##### Data Analysis

ERFs for *L*_den_ were provided by the authors of ten railway noise studies, aggregating data from 10,970 study participants. The studies included are characterized by a variety of potentially confounding or moderating variables: vibration (one study), valley areas and public discussions about the negative consequences of increased freight rail traffic (four studies), and high-speed trains (Shinkansen; two studies). As done with the other noise sources, we calculated the percentages for 5 dB steps from 40 to 80 dB within the empirical range of levels used in the respective study. The *L*_Aeq,24h_-values for railway noise provided by the French study (Champelovier et al., 2003) were transformed to *L*_den_ using the formula given by Brink [39]: *L*_den_ = *L*_Aeq,24h_ + 5.9144. 

**Figure 10:**
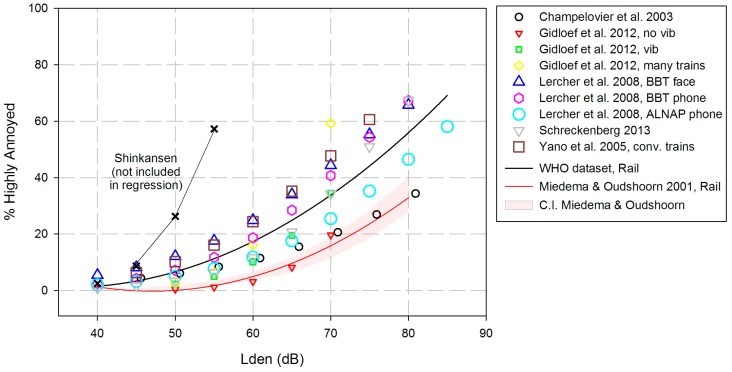
Scatterplot of the relation between *L*_den_ and %HA including ten railway noise studies. The quadratic regression (black line) was calculated excluding the Shinkansen data. In addition, the exposure-response function by Miedema and Oudshoorn ([4], railway, red curve) is shown together with the confidence interval. Notes: (1) The size of the data points corresponds to the number of participants in the respective study (size = SQRT(N)/10). (2) If two results from different studies fall on the same data point, the last point plotted may mask the former one. (3) The black curve is derived from aggregated data, while the red one is derived from individual data.

Right at the start, it was observed that the Yano-Shinkansen study had an ERR which differed considerably from all of the other studies (see Figure 10). Potential causes for this observation may be an infrastructure change effect, together with elevated tracks, and strong vibrations. Therefore, we excluded the Shinkansen study from the estimation of a common ERR.

The scatterplot of the nine railway noise studies shows a certain spread around the black regression line, but the overall fit of the regression is higher than has been observed with the other two transportation noise sources: R^2^ = 0.79.

The equation for estimated %HA by *L*_den_ levels of railway noise is:

Estimated %HA = 38.1596 − 2.05538 × *L*_den_ + 0.0285 × *L*_den_^2^.

If we try to compare the ERF estimated from aggregated (and rather coarse) data with the old Miedema/Oudshoorn [4] curve, we have to keep in mind that the circumstances for comparison are far from ideal:

(1) The number of studies is rather small in both data sets—each includes nine studies; the older ones contain two tramway studies, the newer ones only long-distance lines in a variety of situations (see next paragraph).
(2) The reasons presented in Section 3.2 relating to the exclusion of the Alpine and Asian studies from a common road traffic noise exposure-response curve should be applied here, too: four of the nine rail studies took place in valleys and are subject to an “amphitheater effect”, and the Japanese study includes respondents mostly living in air-conditioned houses. (In this case, it should be mentioned that Japanese houses often are built close to the railway tracks, and are prone to vibrations). In addition, the four studies performed in valleys underwent long-lasting public discussion about the possible effects of railway noise. However, excluding five of nine studies from the full set of eleven studies would not allow for providing any exposure-response curve at all. Therefore, we refrained from additional exposure-response analyses in subsets of data.
(3) The definition of HA differs between the two datasets: While the EU standard curves use a cut-off at 72% of the response scale, five of the present studies define HA by the upper two scale points of the 5-point scale, i.e., HA: ≥60% of the response scale.

At least two of the study characteristics, respondents living in a valley, and defining HA by means of the 60%-criterion, may have contributed to the increased percentages of highly annoyed people in the new dataset. It still seems remarkable that just the 5th percentiles (not shown here) of the new curve based on aggregated estimations are included in the upper limit of the Miedema/Oudshoorn [4] curves’ confidence interval. This underlines the necessity to re-evaluate the old railway exposure-response relation.

##### Grading the Quality of Evidence for the ERR of %HA by Railway Traffic Noise

We are moderately confident in the evidence with respect to ERRs between railway noise levels and percentage of high railway traffic noise annoyance, and assign the grade “moderate quality” (see Supplementary Materials S24).

#### 3.3.2. Railway Noise Effects (2): Correlations between Noise Levels and Annoyance Raw Scores

##### Meta-Analysis in the Full Dataset

**Figure 11:**
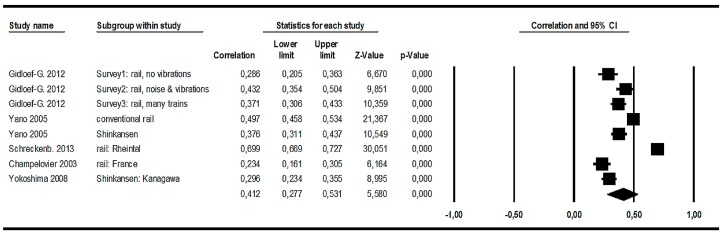
Meta-analysis of eight studies using Pearson correlations between *L*_Aeq,24h_ and railway noise annoyance raw scores. The right part of the graph contains a forest plot of the correlations and their respective 95% confidence intervals. The figures of the last row indicate the summary estimates.

Eight of the 11 studies provided correlations between *L*_Aeq,24h_ and railway noise annoyance raw scores. It is usually more difficult (less valid) to calculate *L*_den_ from reported *L*_Aeq,24h_ levels for railway noise compared to other transportation sources, because valid data for railway noise with respect to the traffic distribution over the course of a day (in particular the evening) is not always available. Therefore, it was decided to choose the *L*_Aeq,24h_ instead of *L*_den_ for the meta-analysis. We subjected them (together with the respective n) to a meta-analysis and found a relative large noise effect, but a remarkable variation between studies as well (see Figure 11).

All of the correlations between noise levels and annoyance raw scores are highly statistically significant (*p* < 0.001) and range from 0.234 to 0.699 with confidence intervals with lower limits from 0.161 to 0.669, and upper limits from 0.305 to 0.727. The summary (last row, diamond symbol) shows a highly statistically significant average weighted correlation of 0.412 with a confidence interval from 0.277 to 0.531. In sum, the correlational analysis shows a considerable effect of railway noise levels on railway noise annoyance raw scores, including a remarkable variance between studies. There are indications of publication biases (see the funnel plot in Supplementary Materials S25) as well as between-study heterogeneity (S26). One of the potential factors contributing to the between-study heterogeneity may be the inclusion of the Yano/Shinkansen study. However, omitting this study only results in a slightly increased summary correlation (r = 0.417 vs. r = 0.412; see Supplementary Materials S26).

##### Grading the Evidence Based on Railway Noise Correlations between Noise Levels and Annoyance Raw Scores

We are confident in the evidence regarding the correlations between railway noise levels and railway traffic noise annoyance raw scores, and like to assign the grade “high quality” (see Supplementary Materials S27).

#### 3.3.3. Railway Noise Effects (3): ORs Referring to the Increase of %HA per 10 dB Level Increase

##### Meta-Analysis Based on Observed Data

Seven of the 11 railway noise annoyance studies provided data for the percentage of highly annoyed persons at 50 and 60 dB *L*_Aeq,24h_—just five provided the same data related to *L*_den_. Therefore, we used *L*_Aeq,24h_ data (see Table 6).

**Figure 12:**
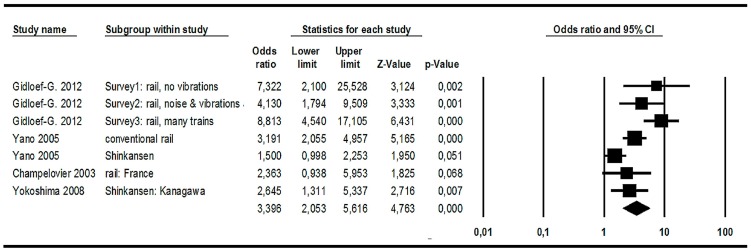
Odds Ratios and 95% confidence intervals from seven studies (based on observed data) for the increase of the rate of “highly annoyed” persons from 50 to 60 dB *L*_Aeq,24h_ railway noise. The right part of the graph contains a forest plot of the ORs and their respective 95% confidence intervals. The figures of the last row indicate the summary estimates.

These percentages were (after dividing by 100 and supplemented by the n of cases at each of the level classes) entered into the meta-analysis program as “event rates”, and converted to ORs. That is, the program calculates the odds from the HA-rates at each exposure class (60 dB *L*_Aeq,24h_ and 50 dB *L*_Aeq,24h_) and converts these to an estimate of the OR (Figure 12). It turned out that in sum, the OR (referring to a 10 dB level increase) is greater than 1 and statistically highly significant (OR = 3.396, 95% CI = 2.053–5.616; *p* < 0.001). The ORs range from 1.5 to 8.8. In general, these ORs referring to the %HA-increase are roughly comparable with the ORs estimated for aircraft noise (see Section 3.1.6). However, the dispersion of ORs for high railway noise annoyance is much larger than that for aircraft noise annoyance. Especially the first study from Sweden calls attention: it shows the second highest OR, but the confidence interval is extraordinary large (from 2.1 to 25.528). It should be noted that the Swedish rail studies are fully comparable with other studies in the correlational analysis, and they have a high study quality rating.

There are indications of publication biases (see the funnel plot in Supplementary Materials S28) as well as between-study heterogeneity (S18). One of the factors contributing to the between-study heterogeneity seems to be the inclusion of the Yano/Shinkansen study. When this study is omitted, the summary OR increases from 3.396 to 4.023, and the heterogeneity slightly decreases (see Supplementary Materials S29).

##### Meta-Analysis of Railway Noise ORs, Based on Modelled Data

Ten of 11 railway noise annoyance studies provided parameters from logistic regression. These data were used in order to calculate ORs referring to the %HA increase per 10 dB level increase. All of them are statistically highly significant. The summary OR is 3.526, which is comparable to the summary OR based on observed data. For more details, see Supplementary Materials S30 and S31.

##### Grading the Evidence of ORs Representing the %HA Increase per 10 dB Level Increase of Railway Noise

We are confident in the evidence of a statistically significant OR referring to the increase of %HA with a 10 dB increase of railway noise levels, but there might be a certain overestimation of the effect, especially with modelled data. In terms of the GRADE system, we assign “moderate quality” to the effects based on original grouped data and “high quality” to the effects based on modelled data (see Supplementary Materials S32).

#### 3.3.4. The Influence of Co-Determinants in Railway Noise Annoyance Studies

In the scientific literature, at least six co-determinants are mentioned, which should be taken into account when analyzing noise annoyance from railway noise: ground-borne vibrations, the distance between residential buildings and railway tracks, the construction type of the building, the relation between passenger trains and freight trains, the relation between conventional passenger trains and high-speed passenger trains, and the availability of a quiet façade at home (see Supplementary Materials S33). Differences between studies with regard to these factors may contribute to their different results.

#### 3.3.5. Summary of the Analyses Related to Railway Noise Effects on Annoyance

A total of 11 individual studies (including 12,477 respondents) on railway noise annoyance provided data for a series of meta-analyses. The correlational analysis, based on seven studies, shows a summary correlation between noise levels and annoyance raw scores of 0.417 (*p* < 0.001; 95% CI = 0.263–0.550). This summary correlation shows that about 17% of the variance of railway noise annoyance raw scores is accounted for by the variance of *L*_Aeq,24h_. However, a large percentage of the variance between studies could not be explained. The meta-analysis based on the observed %HA-difference at 10 dB difference (50 and 60 dB *L*_Aeq,24h_) shows ORs which are greater than 1 and statistically highly significant (including the Yano/Shinkansen study: OR = 3.396; excluding the Yano/Shinkansen study: OR = 4.023). In other words: the chance to be highly annoyed is more than three times higher when the railway noise level increases from 50 to 60 dB. However, a large part of between study variance is left unexplained. A similar analysis, based on modelled data, shows similar results at somewhat lower ORs (3.526 to 3.181). The exclusion of one of the Japanese Shinkansen train studies decreased the heterogeneity to some degree, but did not account for all of the variance. A factor which seems to be systematically related to the between-study variance is the noise level range: studies using a smaller range of noise levels were associated with higher ORs. However, the noise levels in these studies are usually higher than in studies using a larger level range. In other words, we cannot clarify whether higher ORs are due to higher levels, due to the level range, or due to both level characteristics.

A tentative ERR is given in the present report. The estimated ERR between %HA and *L*_den_ is based on a quadratic regression between *L*_den_ and the aggregated (secondary) WHO data set, weighted according to the square root of the total sample size. This curve shows a steeper increase of the %HA with increasing *L*_den_ as compared to the Miedema and Oudshoorn [4] curves on railway annoyance. However, it should be noted that the definition of HA in our dataset is less stringent than the one used in the Miedema and Oudshoorn [4] curves.

### 3.4. Wind Turbine Noise Effects on Annoyance

The two publications [41,42] included in the wind turbine noise annoyance analysis contain descriptions of a total of four individual studies (a total of 2481 respondents). Although there are differences between studies with respect to the annoyance rating (e.g., spatial frame of reference, response scale) and noise descriptor, we performed comparisons between reported ERFs for %HA, increase of %HA with 5 dB level increase, and exposure-response correlations between noise levels and annoyance raw scores. The two comparisons based on %HA (ERFs and increase of %HA with level increase) led to inconsistent results and a low quality of evidence. In contrast, the formal meta-analysis based on correlations between noise levels and annoyance raw scores showed a moderate quality of evidence (summary correlation r = 0.278; *p* = 0.001; 95% CI = 0.11–0.430). It is evident that the level of wind turbine sounds is systematically related to noise annoyance, even at levels below 40 dB *L*_den_. However_,_ the ERR between noise levels and %HA is subject to inconsistency between studies (see Supplementary Materials S34).

### 3.5. Combined Noise Effects

We included five studies on noise source combinations, contained in four publications. All studies include road traffic noise; two of the studies combine road and railway noise, two combine road and aircraft noise, and one combines road and industrial noise. The total dataset includes 1949 respondents. After performing the analyses, however, it became apparent that it is unwise to integrate different noise source combinations in a single analysis. Unfortunately, there were not enough studies available for the meta-analysis of a single source combination. With respect to the weights given for the separate noise levels in future combination studies, our results point to the importance of the dominant source in terms of annoyance. For more information, see Supplementary Materials S35.

### 3.6. Effects of Noise from Stationary Sources

We simply describe the results from an overview given by Miedema and Vos [43]. Details are presented in Supplementary Materials S36.

## 4. Discussion

The systematic review presented here has three major goals: (a) to assess the strength of association between exposure to environmental noise and long-term noise annoyance, (b) to quantify the increase of annoyance with an incremental increase in noise exposure, and (c) to estimate an ERR for each noise source. Since the review used field research reported between the years 2000 and 2014, a comparison with earlier results is obligatory wherever possible.

Generally, we found moderate to high quality evidence for statistically significant correlations between noise levels and annoyance raw scores with respect to aircraft, road, rail, and noise source combinations. We also found moderate to high quality evidence for the increase of %HA (expressed in terms of OR) with a 10 dB increase in levels of aircraft, road, and rail noise, while we judged the comparable effects with wind turbine noise to be of low quality.

It turned out that ERRs between noise levels (in terms of *L*_den_) and the percentage of highly annoyed persons (%HA) partially differ between newer studies (2000–2014) and older ones (before 2000). This can be seen especially with aircraft noise as %HA in the more recent studies is usually higher at the same *L*_den_ levels (Figure 2) than in the so-called European Standard curves [4]. Some of the newer road and railway studies show a similar increase in %HA: the new road traffic noise studies reveal a considerable increase of %HA at noise levels between 40 and 60 dB *L*_den_ in the full dataset (Figure 6). On the other hand, there is an increase of %HA at levels above 70 dB *L*_den_ in the dataset excluding the Alpine and Asian road traffic datasets (Figure 7). In contrast to the diverging road traffic noise ERRs, the new railway ERR shows an increase of %HA at all levels above 45 dB *L*_den_ (Figure 10). At present, the causes of these differences are not clear; they may be due to co-determinant factors, like vibration, valleys, or high traffic volume as well as other factors, such as societal factors.

For years, the rise of %HA with respect to aircraft noise has been a matter of debate, and some reasons have been discussed and tested by Janssen and her group [5,20,37]. They found the type of annoyance scale, the type of contact, and the response percentage to be sources of heterogeneity between old and new studies, but only the scale factor was systematically associated with the study year. That is, the numerical 11-point version of the ICBEN/ISO response scale was increasingly used in newer studies, while the old studies mostly used verbal 5-point scales. This result may be interpreted in the sense that using the 11-point numeric scale may be associated with higher annoyance, compared to using the 5-point verbal scale. Brink [44] reports a similar effect in a systematic field experiment. Another study by Brink et al. [45], however, showed closer associations between noise levels and annoyance expressed by means of the verbal 5-point scale, as compared to the numeric 11-point scale. We found the situational context of the survey to be associated with the percentage of highly annoyed respondents: participants living in the context of “airport change” tend to express higher noise annoyance, as compared to participants in “no change” conditions. Due to a lack of data, we could not systematically analyse effects of the number of loud events, the use of software models for calculating the noise levels, the influence of response formats, and the influence of moderating variables.

With respect to railway noise annoyance, we also found the exposure-response curve for %HA at higher *L*_den_ levels to be well above the European standard curve [4]. This may partially be due to different definitions of “high annoyance”, partially due to the increased number of freight trains in the sample of newer surveys, and partially be due to other factors (see above). The reasons for the differences between “old” and “new” results could not be analysed systematically within the scope of this review, and we suggest doing so by means of original data before deciding upon a revision of the earlier curve.

## 5. Conclusions

The analysis of newer surveys (2000–2014) on annoyance due to traffic noise shows statistically significant correlations between noise levels and annoyance scores with moderate strength of the relationship. Summary correlations between noise levels and annoyance raw scores vary from 0.33 (road) to 0.44 (aircraft and noise source combinations). The statistical relations between wind turbine noise levels and annoyance are less clear. The ORs referring to the %HA for a 10 dB increase in traffic noise levels vary from 2.7 (road) to 4.0 (rail) for observed data, and from 3.0 (road) to 4.8 (aircraft) for modelled data (with railway noise at 3.2, and 3.5, resp.). An OR equal to 3 means that the chance to be highly annoyed is three times higher than at base level. With respect to aircraft and railway noise, we observed an increase of the percentage of highly annoyed residents as compared to the so-called EU standard curves.

## Figures and Tables

**Table 1:** Aircraft noise annoyance studies included.

Publication (See Supplementary Materials S3 for References)	Location	Year Data	Sample Type	Type of Survey	Sample Size (n)	Response Rate (RR)	Age/Age Range	Noise Level Descriptors	Noise Level Range	Annoyance Scale	Remarks	Study Quality Rating
Babisch et al. 2009	Amsterdam Schiphol, The Netherlands	2003–2005	Stratified.	face-to-face interview	898	46%	45–70 years	*L*_Aeq,24h_	36–72 *	ICBEN 11-p num.	New runway opened 2003	23
			Persons (living for at least 5 years near the airport) selected at random from registers					*L*_Aeq,16h_	38–74	Annoyance during the day and during the night were assessed separately in the HYENA study. Only annoyance during the day is used here.		
								*L*_den_	40–75			
								*L*_dn_	39–77	HA ≥ 73%		
Babisch et al. 2009	Athens Elephterios Venizelos, Greece	2003–2005	Stratified; random (see the first entry above)	face-to-face interview	635	56%	45–70 years	*L*_Aeq,24h_	36–64	ICBEN 11-p num.	Airport opened 2001	23
								*L*_Aeq,16h_	37–66	Annoyance during the day.		
								*L*_den_	40–66			
								*L*_dn_	39–64	HA ≥ 73%		
Babisch et al. 2009	Berlin Tegel, Germany	2003–2005	Stratified; random (see the first entry above)	face-to-face interview	972	“not less than 30% in Germany…”	45–70 years	*L*_Aeq,24h_	30–73	ICBEN 11-p num.		23
								*L*_Aeq,16h_	32–74	Annoyance during the day.		
								*L*_den_	32–76			
								*L*_dn_	31–74	HA ≥ 73%		
Babisch et al. 2009	London Heathrow, UK	2003–2005	Stratified; random (see the first entry above)	face-to-face interview	600	“not less than 30% in… the UK”	45–70 years	*L*_Aeq,24h_	29–74	ICBEN 11-p num.		23
								*L*_Aeq,16h_	31–76	Annoyance during the day.		
								*L*_den_	34–78			
								*L*_dn_	32–77	HA ≥ 73%		
Babisch et al. 2009	Milano Malpensa, Italy	2003–2005	Stratified; random (see the first entry above)	face-to-face interview	753	“not less than 30% in… Italy”	45–70 years	*L*_Aeq,24h_	22–68	ICBEN 11-p num.	Airport expanded 1998. Long lasting public discussion about expansion	23
								*L*_Aeq,16h_	24–70	Annoyance during the day.		
								*L*_den_	22–70			
								*L*_dn_	22–68	HA ≥ 73%		
Babisch et al. 2009	Stockholm Arlanda + Brömma, Sweden	2003–2005	Stratified; random (see the first entry above)	face-to-face interview	1003	78%	45–70 years	*L*_Aeq,24h_	11–64	ICBEN 11-p num.	New runway 2003	23
								*L*_Aeq,16h_	13–66	Annoyance during the day.		
								*L*_den_	12–68			
								*L*_dn_	11–67	HA ≥ 73%		
Bartels et al. 2013	Cologne/Bonn, Germany	2010	Random within 3 exposure classes (40, 50, 55 *L*_dn_)	Phone interview	1262	More than 4000 numbers dialed; 9.2% not valid; 34.1% persons interested to take part	18–95 years	*L*_Aeq,24h_	40–55	ICBEN 5-p verbal (general + night)	Night-time air traffic	20
				CATI				*L*_Aeq,6–22h_	40–55			
								*L*_Aeq,22–6h_	40–55	HA ≥ 60%		
								*L*_dn_	46–61			
Breugelmans et al. 2004	Amsterdam Schiphol, The Netherlands	2002	Stratified;	Written questionnaire; mailed	5873	46.10%	Age: ≥18 years	*L*_den_	33–72	ICBEN 11-p num.	New runway 2003	23
			Randomly selected within strata									
										HA ≥ 73%		
Brink et al. 2008	Zurich, Switzerland	2001	Random within 20 km from airport	Written questionnaire; mailed	1816	54%	18–98 years	*L*_Aeq,24h_	22–69	ICBEN 11-p num.	Change of flights in October 2001. Only data before change are used here	23
								*L*_Aeq,16h_	35–70			
								*L*_den_	35–70	HA ≥ 73%		
								*L*_dn_	36–70			
Gelderblom et al. 2014	Trondheim, Norway	2014	Random within 55 dB *L*_dn_ contour	phone	300		16–92	*L*_Aeq,24h_	36–65	ICBEN 11-p num.	Only Trondheim (civil airport) is used here	21
								*L*_Aeq,16h_	37–66			
								*L*_den_	40–68	HA ≥ 73%		
								*L*_dn_	39–68			
Nguyen et al. 2011	Ho Chi Minh Tan Son Nhat, Vietnam	2008	8 sites under flight path + 2 control sites.	Face-to-face	880	87%	Age: >18 years	*L*_Aeq,24h_	49–66	ICBEN 5-p verbal + 11-p num.	Only data for aircraft noise are used here.	16
			Convenience sample; selection with regard to age (>18 years) and gender					*L*_day_	50–67			
								*L*_den_	53–71	HA ≥ 73%		
								*L*_dn_	53–70	(for the 11p scale)		
Nguyen et al. 2011	Hanoi Noi Bai, Vietnam	2009	7 sites under flight path + 2 control sites.	Face-to-face	824	84%	Age: >18 years	*L*_Aeq,24h_	44–57	ICBEN 5-p verbal + 11-p num.	Only data for aircraft noise are used here.	16
			Convenience sample (see above)					*L*_day_	46–58			
								*L*_den_	48–61	HA ≥ 73%		
								*L*_dn_	48–61			
Nguyen et al. 2012	Da Nang, Vietnam	2011	6 sites around the airport	Face-to-face	528	84%		*L*_Aeq,24h_	49–60	ICBEN 5-p verbal + 11-p num.		17
								*L*_day_	51–62	HA ≥ 73%		
								*L*_den_	52–64			
								*L*_dn_	51–63			
Sato & Yano 2011	Sapporo Okadama, Japan	2008	5 sites around the airport.	Postal	291	76%	Age: >18 years	*L*_Aeq,24h_	28–40	ICBEN 5-p verbal + 11-p num.	Only data for airplane noise are used	16
			Respondents (age >18 years) selected on a one-person-per-family basis.					*L*_den_	28–40			
								*L*_dn_	28–40	HA ≥ 73% (for the 11p-scale)		
Schreckenberg + Meis 2007	Frankfurt/M, Germany	2005	Stratified; random	Face-to-face	2312	61%	Age: 17–93 years; (M = 52.7; s = 18.4)	*L*_Aeq,24h_	40–62	ICBEN 5-p verbal + 11-p num.	Long lasting public discussion about airport expansion. New runway opened 2011	24
								*L*_Aeq,16h_	41–63			
								*L*_den_	43–66	HA ≥ 73% (for the 11 p-scale)		
								*L*_dn_	42–65			

* Cut-off values used in most analyses: for *L*_Aeq,24h_: 35 dB; for *L*_Aeq,16h_: 40 dB; for *L*_den_: 40 dB; for *L*_dn_: 40 dB. Note: The study quality rating has been done by means of a list of criteria, comprising six dimensions: Overall survey design, Social survey sample, Social survey data collection, Nominal acoustical conditions, Basic exposure-response analysis, and Explanatory variable analysis. More information is given in Supplementary Materials S2.

**Table 2:** Air traffic noise—HA rates and the number of respondents in two classes of exposure (*L*_den_).

Study	Subgroup	Midpoints of the Two Exposure Classes	HA Rate in the Upper dB Class	Number of Respondents in the Upper dB Class	HA Rate in the Lower dB Class	Number of Respondents in the Lower dB Class
Babisch-Hyena	GB (Heathrow)	63 vs. 53	0.424	170	0.300	70
Babisch-Hyena	D (Tegel)	63 vs. 53	0.398	171	0.287	94
Babisch-Hyena	NL (Schiphol)	63 vs. 53	0.259	286	0.068	191
Babisch-Hyena	SWE (Arlanda)	63 vs 53	0.271	48	0.145	55
Babisch-Hyena	GR (Athens)	63 vs. 53	0.690	58	0.530	151
Babisch-Hyena	I (Milan Malpensa)	63 vs. 53	0.703	101	0.427	103
Brink 2008	Zurich before 2001	60 vs. 50	0.327	199	0.074	457
Schreckenberg & Meis 2007	Fraport	60 vs. 50	0.413	611	0.139	603
Nguyen 2011	Hanoi	60 vs. 50	0.395	190	0.085	259
Nguyen 2012	Da Nang	60 vs. 50	0.163	257	0.030	67
Gelderblom 2015 *	Trondheim	60 vs. 50	0.038	52	0	76

* This study has not been used in the meta-analysis on observed data because of 0%HA at 50 dB. An extended discussion of the problems related to 0% is given in Supplementary Materials S34.

**Table 3:** Road traffic noise studies included.

Publication (See Supplementary Materials S3 for References)	Location	Year Data	Sample Type	Type of Survey	Sample Size (n)	Response Rate (RR)	Age/Age Range	Noise Level Descriptors	Noise Level Range	Annoyance Scale	Remarks	Study Quality Rating
Babisch et al. 2009	Amsterdam Schiphol, The Netherlands	2003–2005	Stratified. Persons (living for at least 5 years near the airport) selected at random from registers.	face-to-face interviews	898	46%	45–70 years	*L*_Aeq,24h_	36–74 *	ICBEN 11-p numeric. Annoyance during the day and during the night were assessed separately in the HYENA study. Used here: only the annoyance during the day. HA ≥ 73%.		23
								*L*_Aeq,16h_	37–75			
								*L*_den_	39–77			
								*L*_dn_	39–77			
Babisch et al. 2009	Athens Elephterios Venizelos, Greece	2003–2005	Stratified; random (see the first entry above)	face-to-face interviews	635	56%	45–70 years	*L*_Aeq,24h_	10–69 *	ICBEN 11-p numeric. Annoyance during the day. HA ≥ 73%		23
								*L*_Aeq,16h_	10–70			
								*L*_den_	16–72			
								*L*_dn_	16–71			
Babisch et al. 2009	Berlin Tegel, Germany	2003–2005	Stratified; random (see the first entry above)	face-to-face interviews	972	“not less than 30% in Germany …”	45–70 years	*L*_Aeq,24h_	45–73 *	ICBEN 11-p numeric. Annoyance during the day. HA ≥ 73%		23
								*L*_Aeq,16h_	46–74			
								*L*_den_	45–77			
								*L*_dn_	46–76			
Babisch et al. 2009	London Heathrow, UK	2003–2005	Stratified; random (see the first entry above)	face-to-face interviews	600	“not less than 30% in … the UK”	45–70 years	*L*_Aeq,24h_	40–75 *	ICBEN 11-p numeric. Annoyance during the day. HA ≥ 73%		23
								*L*_Aeq,16h_	41–76			
								*L*_den_	42–77			
								*L*_dn_	42–76			
Babisch et al. 2009	Milano Malpensa, Italy	2003–2005	Stratified; random (see the first entry above)	face-to-face interviews	753	“not less than 30% in … Italy”	45–70 years	*L*_Aeq,24h_	25–77 *	ICBEN 11-p numeric. Annoyance during the day. HA ≥ 73%		23
								*L*_Aeq,16h_	26–78			
								*L*_den_	25–79			
								*L*_dn_	22–78			
Babisch et al. 2009	Stockholm Arlanda + Brömma, Sweden	2003–2005	Stratified; random (see the first entry above)	face-to-face interviews	1003	78%	45–70 years	*L*_Aeq,24h_	25–71 *	ICBEN 11-p numeric. Annoyance during the day. HA ≥ 73%		23
								*L*_Aeq,16h_	26–72			
								*L*_den_	28–74			
								*L*_dn_	27–73			
Brink 2013	German speaking Switzerland	2012–2013	Stratified; random	Written questionnaire, mailed	2386			*L*_Aeq,24h_	42–75	ICBEN 5-p & 11-p	Data pooled from two different waves of the same survey. The results from one wave were not part of the Brink 2013 paper.	20
								*L*_Aeq,16h_	44–77			
								*L*_dn_	44–78			
										HA ≥ 73% (for the 11p scale)		
Brown et al. 2015	Hong Kong, China	2009–2010	Random	Face-to-face Interviews conducted by the Census department; routine thematic household survey	10,077	76%	Age: ≥18 years	*L*_den_	30–80 (Most analyses used only the range of 42 to 77 dB)	ICBEN 11-p numeric	High road traffic intensity.	22
										HA ≥ 73%		
Champelovier et al. 2003	France 61 sites all over France	1997–1998	Convenience sample	Face-to-face interviews	701 in total; a subsample with n = 673 used here		Age: ≥18 years	*L*_Aeq,24h_	41–78	4-p verbal scale (inside) & 11p scale.	Only road data used	19
								*L*_Aeq,16h_	42–80			
								*L*_den_	43–81	HA ≥ 73% (for 11p)		
								*L*_dn_	42–81			
Heimann/Lercher 2007; Lercher et al. 2008	Inn valley, Austria	2006	Stratified random sampling; (Strata = distance to source)	Computer-assisted telephone interviewing	1641	35%	25–75 years	*L*_den_		ICBEN 5-p verbal	Alpine areas, Main road. The Inn valley is part of a route for heavy goods traffic over the Brenner. Long lasting public discussion about road traffic noise.	22
										HA ≥ 60%		
Heimann/Lercher 2007; Lercher et al. 2008	Inn valley, Austria	2006	Stratified random sampling; (Strata = distance to source)	Computer-assisted telephone interviewing	1641	35%	25–75 years	*L*_den_		ICBEN 5-p verbal	Alpine areas, Highway. The Inn valley is part of a route for heavy goods traffic over the Brenner. Long lasting public discussion about road traffic noise.	22
										HA ≥ 60%		
Pierrette et al. 2012	Near Lyon, France	?	Residents living near an industrial site and surrounded by two roads	Face-to-face interviews	99		Mean age: 45.9 years (s = 17.9)	*L*_den_	43–70	ICBEN 5-p & 11-p	Only road data used.	20
Med.Univ. Innsbruck/Lercher 2008	Wipp valley, Austria	2004	Stratified (distance)	Face to face	1991	80%	17–85 years	*L*_den_		ICBEN 11-p numeric	Alpine areas, Main road The Wipp valley is part of a route for heavy goods traffic over the Brenner. Long lasting public discussion about road traffic noise.	22
										HA ≥ 73%		
Med.Univ. Innsbruck Lercher/2008	Wipp valley, Austria	2004	Stratified (distance)	Face to face interviews	1762	80%	17–85 years	*L*_den_		ICBEN 11-p numeric	Alpine areas; Highway The Wipp valley is part of a route for heavy goods traffic over the Brenner. Long lasting public discussion about road traffic noise.	22
										HA ≥ 73%		
Med.Univ. Innsbruck Lercher/2008	Wipp valley, Austria	2004	Stratified (distance)	Phone	1327	62%	17–85 years	*L*_den_		ICBEN 5-p verbal	Alpine areas. Motorway + main road (others below 40 dB(A)) The Wipp valley is part of a route for heavy goods traffic over the Brenner. Long lasting public discussion about road traffic noise.	22
										HA ≥ 60%		
Sato et al. 2002	Gothenburg, Sweden, detached	1996	11–15 residential areas. Respondents randomly selected on a one person-per-family basis	Written questionnaire; by mail	436	73.3%	18–75 years	*L*_Aeq,24h_	46.2–73.6	4-p verbal scale plus “notice filter”		14
								*L*_dn_	50.1–76.9	HA ≥ 75%		
Sato et al. 2002	Gothenburg, Sweden, Apartments	1996	11–15 residential areas. Respondents randomly selected on a one person-per-family basis	Written questionnaire; by mail	706	66.4%	18–75 years	*L*_Aeq,24h_	48.5–82.3	4-p verbal scale plus “notice filter”		14
								*L*_dn_	51.8–86.2			
										HA ≥ 75%’		
Sato et al. 2002	Kumamoto, Japan, detached	1996	11–15 residential areas. Respondents randomly selected on a one person-per-family basis	Written questionnaire; distribute-collect method	378	76.1%	20–75 years	*L*_Aeq,24h_	49.3–73.7	4-p verbal scale plus “notice filter”		14
								*L*_dn_	52.4–76.8			
										HA ≥ 75%		
Sato et al. 2002	Kumamoto, Japan, Apartments	1996	11–15 residential areas. Respondents randomly selected on a one person-per-family basis	Written questionnaire; distribute-collect method	458	64.6%	20–75 years	*L*_Aeq,24h_	51.3–73.5	4-p verbal scale plus “notice filter”		14
								*L*_dn_	54.4–78.7			
										HA ≥ 75%		
Sato et al. 2002	Sapporo, Japan, detached	1997–1998	11–15 residential areas. Respondents randomly selected on a one person-per-family basis	Written questionnaire; distribute-collect method	411	63.5%	20–75 years	*L*_Aeq,24h_	53.3–73.6	4-p verbal scale plus “notice filter”		14
								L_dn_	57.5–77.5			
										HA ≥ 75%		
Sato et al. 2002	Sapporo, Japan, Apartment	1997–1998	11–15 residential areas. Respondents randomly selected on a one person-per-family basis	Written questionnaire; distribute-collect method	369	52.0%	20–75 years	*L*_Aeq,24h_	52.1–70.7	4-p verbal scale plus “notice filter”		14
								*L*_dn_	56.3–75.8			
										HA ≥ 75%		
Shimoyama et al. 2014 **	Hanoi, Vietnam	2005	8 sites. One Member from each household in the selected sites.	Face-to-face.	1503	50%	Age: >18 years (Most of the respondents were in their 20s)	*L*_Aeq,24h_	64.5–76.5	ICBEN 5-p & 11-p	Motorbikes are the most dominant traffic constituent.	11
										HA ≥ 73% (for the 11p scale)		
								*L*_den_	69.5–81.2			
Shimoyama et al. 2014 **	Ho Chi Minh City, Vietnam	2007	8 sites. One Member from each household in the selected sites.	Face-to-face	1471	61%	Age: >18 years	*L*_Aeq,24h_	70.3–78.5	ICBEN 5-p & 11-p	Motorbikes are the most dominant traffic constituent	11
								*L*_den_	74.9–83.1			
										HA ≥ 73%		
Shimoyama et al. 2014 **	Da Nang, Vietnam	2011	6 sites.	Face-to-face	492	82%	Age: >18 years	*L*_Aeq,24h_	63.3–72.1	ICBEN 5-p & 11-p		11
								*L*_den_	66.4–75.8	HA ≥ 73%		
Shimoyama et al. 2014 **	Hue, Vietnam	2012	7 sites	Face-to-face	688	98%	Age: >18 years	*L*_Aeq,24h_	58.0–75.6	ICBEN 5-p & 11-p		11
								*L*_den_	60.9–79.6	HA ≥ 73%		
Shimoyama et al. 2014 **	Thai Nguyen, Vietnam	2013	10 sites	Face-to-face	813	81%	Age: >18 years	*L*_Aeq,24h_	57.8–73.7	ICBEN 5-p & 11-p		11
								*L*_den_	60.9–77.9	HA ≥ 73%		

* A cut-off at 45 dB(A) for road traffic noise was used in most analyses of the HYENA study. ** The Shimoyama et al. data were kindly provided by Thu Lan Nguyen.

**Table 4:** Road traffic noise—HA rates and the number of respondents in two classes of exposure (*L*_den_ or *L*_dn_ resp.).

Study (See Supplementary Materials S3 for References)	Subgroup	Midpoints of the Two Exposure Classes	Exposure Descriptor	HA Rate in the Upper dB Class	Number of Respondents in the Upper dB Class	HA Rate in the Lower dB Class	Number of Respondents in the Lower dB Class
Babisch-Hyena	D (Tegel)	60 vs. 50	*L*_den_	0.288	156	0.069	189
Babisch-Hyena	GB (Heathrow)	60 vs. 50	*L*_den_	0.224	98	0.092	174
Babisch-Hyena	GR (Athens)	60 vs. 50	*L*_den_	0.154	26	0.042	95
Babisch-Hyena	I (Milano Malpensa)	60 vs. 50	*L*_den_	0.209	115	0.077	78
Babisch-Hyena	NL (Schiphol)	60 vs. 50	*L*_den_	0.115	139	0.072	195
Babisch-Hyena	SWE (Arlanda)	60 vs. 50	*L*_den_	0.125	72	0.023	341
Brink 2013	CH, Vers. 2	60 vs. 50	*L*_dn_	0.129	652	0.035	58
Champelovier 2003	France	60 vs. 50	*L*_den_	0.081	161	0.030	33
Brown 2015	Hong Kong	60 vs. 50	*L*_den_	0.060	3037	0.044	1089
Sato 2002	Gothenburg Apartment	65 vs. 55	*L*_dn_	0.134	217	0.060	50
Sato 2002	Gothenburg Detached	65 vs. 55	*L*_dn_	0.252	143	0.100	40
Sato 2002	Kumamoto Apartment	65 vs. 55	*L*_dn_	0.146	89	0.103	39
Sato 2002	Sapporo Detached	70 vs. 60	*L*_dn_	0.243	189	0.094	32
Sato 2002	Sapporo Apartment	70 vs. 60	*L*_dn_	0.332	187	0.030	33
Sato 2002	Kumamoto Detached	70 vs. 60	*L*_dn_	0.268	112	0.114	70
Shimoyama 2014	Hanoi	80 vs. 70	*L*_den_	0.523	704	0.290	31
Shimoyama 2014	Ho Chi Minh City	80 vs. 70	*L*_den_	0.406	1423	0	
Shimoyama 2014	Da Nang	80 vs. 70	*L*_den_	0		0.0402	199
Shimoyama 2014	Hue	70 vs. 60	*L*_den_	0.0616	292	0.0213	47
Shimoyama 2014	Thai Nguyen	80 vs. 60	*L*_den_	0.4667	90	0.0154	65

**Table 5:** Railway noise studies included.

Publication (See Supplementary Materials S3 for References)	Location	Year Data	Sample Type	Type of Survey	Sample Size (n)	Response Rate (RR)	Age/Age Range	Noise Level Descriptors	Noise Level Range	Annoyance Scale	Remarks	Study Quality Rating
Champelovier et al. 2003	61 sites all over France	1997–1998	Convenience sample	Face-to-face interviews	701 in total; a subsample with n = 673 used here		Age: ≥18 years	*L*_Aeq,24h_	38–79	4-p verbal scale (inside) & 11p scale.	Only rail data used. Noise from TGV and from conventional trains	19
								*L*_Aeq,16h_	36–79	HA ≥ 73% (for 11p)		
								*L*_den_	43–85			
								*L*_dn_	43–84			
Gidlöf-Gunnarsson et al. 2012	Area with 2 different study sites in Sweden	2007–2008	Stratified	Postal; questionnaire sent by mail	521	53% (Total RR for the three Swedish studies)	Age: 18–75 years	*L*_Aeq,24h_	41–65	ICBEN 5-p & 11-p	No vibrations. 124 trains/24 h (44 freight trains)	20
								*L*_den_	48–72	HA ≥ 60%		
Gidlöf-Gunnarsson et al. 2012	Area with 2 different study sites in Sweden	2007–2008	Stratified	Postal; questionnaire sent by mail	459	53% (Total RR for the three Swedish studies)	Age: 18–75 years	*L*_Aeq,24h_	41–64	ICBEN 5-p & 11-p	Noise + vibration. 206 or 179 trains resp./24 h (48 or 22 freight trains resp./24 h)	21
								*L*_den_	48–71	HA ≥ 60%		
Gidlöf-Gunnarsson et al. 2012	Area with one study site in Sweden	2007–2008	Stratified	Postal; questionnaire sent by mail	715	53% (Total RR for the three Swedish studies)	Age: 18–75 years	*L*_Aeq,24h_	45–66	ICBEN 5-p & 11-p	Many trains: 481 trains/24 h (15 freight trains)	21
								*L*_den_	49–70	HA ≥ 60%		
Lercher et al. 2008	Wipp valley, Austria	2004	Stratified; random (Strata = distance to source)	Face-to-face interviews	2017 in total; a subsample with n = 1449 used here	80%	17–85 years	*L*_den_		ICBEN 11-p	Alpine areas; only rail data used. High proportion of freight trains. Public discussion about rail traffic noise	22
										HA ≥ 73%		
Lercher et al. 2008	Wipp valley, Austria	2004	Stratified; random	Phone-interviews	2002 in total, a subsample with n = 1081 used here	62%	17–85 years	*L*_den_		ICBEN 5-p HA ≥ 60%	Alpine areas; only rail data used. High proportion of freight trains. Public discussion about rail traffic noise	22
Lercher et al. 2008 Heimann/ Lercher 2007	Inn valley, Austria	2006	Stratified; random	Phone-interviews	1643	35%	25–75 years.	*L*_den_		ICBEN 5-p	Alpine areas; only rail data used. Noise barriers were erected before interviews. High proportion of freight trains. Public discussion about rail traffic noise	22
										HA ≥ 60%		
Schreckenberg 2013	Railway Rhine valley, Germany	2010	random sampling in 2 areas	Phone-interviews	1211 Respondents. (Main sample: n = 1005; supplemental sample: n = 206).	Main sample: response rate: 41%. Supplemental sample: response rate: 58%.	16–95 years	*L*_Aeq,24h_	37–86	ICBEN 5-p	Long lasting public discussion about railway noise. High proportion of freight trains; many freight trains during the night.	24
								*L*_den_	44–93	HA ≥ 60%		
Yano et al. 2005	Fukuoka Prefecture, Japan	2002	All Detached houses in railway vicinity	Written questionnaire; distribute- collect method	1612	64%		*L*_Aeq,24h_	24–78	ICBEN 5-p + 11-p.	Conventional trains. 52–381 trains per day.	14
								*L*_den_	30–82	The 11-p-scale used here with HA ≥ 73%		
								*L*_dn_	30–82			
Yano et al. 2005	Fukuoka Prefecture, Japan	2003	Detached houses in railway vicinity; one person per family; random selection	Written questionnaire; distribute-collect method	724	66%	20–75 years	*L*_Aeq,24h_	32–50	ICBEN 5-p + 11-p.	Shinkansen trains + vibration. 180 trains per day.	14
								*L*_den_	36–54	The 11-p-scale used here with HA ≥ 73%		
								*L*_dn_	35–53			
Yokoshima et al. 2008	Kanagawa, Japan (Data from Kanagawa and Fukuoka; but only data from Kanagawa used here; see Yano et al. 2005 for Shinkansen in Fukuoka)	2001–2002	Detached houses in railway vicinity	Distribution-by-mail: Questionnaires distributed at 98 survey sites	872 respondents. (114 from 986 excluded because of aircraft noise).	55%	Age: ≥18 years	*L*_Aeq,24h_	28–61	ICBEN 5-p	Shinkansen trains. 287 and 180 trains per day, resp.	13
								*L*_dn_	31–64	HA ≥ 73% (after weighting of the category “4” by 0.4) used here		

**Table 6:** Railway traffic noise—HA rates and the number of respondents in two classes of exposure (*L*_Aeq,24h_).

Study (See Supplementary Materials S3 for References)	Subgroup	Type of Rail	Midpoints of the Two Exposure Classes	HA Rate in the Upper dB Class	Number of Respondents in the Upper dB Class	HA Rate in the Lower dB Class	Number of Respondents in the Lower dB Class
Gidloef-G. 2012	Survey1: rail, no vibrations	conventional	60 vs. 50	0.130	48	0.020	230
Gidloef-G. 2012	Survey2: rail, noise & vibrations	conventional	60 vs. 50	0.290	45	0.090	167
Gidloef-G. 2012	Survey3: rail, many trains	conventional	60 vs. 50	0.360	128	0.060	220
Yano 2005	conventional rail	conventional	60 vs. 50	0.353	292	0.146	226
Yano 2005	Shinkansen	Shinkansen	60 vs. 50	0.338	160	0.254	346
Champelovier 2003	rail: France	Conv. + TGV	60 vs. 50	0.157	178	0.073	82
Yokoshima 2008	Shinkansen: Kanagawa	Shinkansen	60 vs. 50	0.483	36	0.261	305

## Supplementary Materials

The following are available online at www.mdpi.com/1660-4601/14/12/1539/s1, S1. Author’s questionnaires; S2. Items used for rating the study quality; S3. List of papers included/excluded in the Evidence Review on Noise; S4. Grading the quality of evidence for the exposure-response relation of %HA by aircraft noise; S5. Correlations between aircraft noise annoyance raw scores and weighted vs. unweighted 24-h-noise levels; S6. An analysis to detect a bias in reported correlations between aircraft noise annoyance raw scores and aircraft noise; S7. Exploring the heterogeneity of correlations between annoyance raw scores and noise levels; S8. Grading the quality of evidence for the correlation between aircraft noise levels and annoyance; S9. Figure S2. Funnel plot OR and %HA-difference for aircraft noise studies; S10. Exploring the heterogeneity of between-study heterogeneity of Odds Ratios in original grouped data; S11. Meta-analysis based on modelled data; S12. Grading the evidence based on Odds Ratios representing the %HA increase by a 10 dB *L*_den_-increase of aircraft noise; S13. The influence of co-determinants in aircraft noise studies; S14. Grading the quality of evidence for the exposure-response relation of %HA by road traffic noise in the full WHO dataset; S15. Exploring the heterogeneity between road traffic noise studies with respect to correlations; S16. Grading the evidence based on road traffic noise correlations; S17. Figure S5. Funnel plot of the relation between OR and %HA difference effect for road traffic noise, based on observed data; S18. Exploring the between-study heterogeneity of Odds Ratios in original grouped road traffic noise data; S19. Meta-analysis based on modelled data; S20. Figure S7. Funnel plot of the relation between OR and %HA difference effect for road traffic noise annoyance, based on modelled data; S21. Exploring the between-study heterogeneity of Odds Ratios in modelled road traffic noise data; S22. Grading the evidence of Odds Ratios representing the %HA increase per 10 dB level increase of road traffic noise; S23. The influence of co-determinants in road traffic noise studies; S23. The influence of co-determinants in road traffic noise studies; S24. Grading the quality of evidence for the exposure-response relation of %HA by railway traffic noise; S25. Figure S8. Funnel plot for the meta-analysis of eight studies using Pearson correlations between *L*_Aeq,24h_ and railway noise and annoyance raw scores; S26. Exploring the heterogeneity between railway noise studies, based on correlations; S27. Grading the evidence based on railway noise correlations; S28. Figure S10. Funnel plot of noise effects based on the increase of %HA by a 10 dB increase (from 50 to 60 dB *L*_Aeq,24h_) railway noise in observed data; S29. Exploring the between-study heterogeneity of Odds Ratios in original grouped data on railway noise annoyance; S30. Meta-analysis of railway noise ORs based on modelled data; S31. Exploring the between study heterogeneity of Odds Ratios based on modelled data on railway noise annoyance; S32. Grading the evidence of Odds Ratios representing the %HA increase per 10 dB level increase of railway noise; S33. The influence of co-determinants in railway noise annoyance studies; S34. Wind turbine noise effects on annoyance; S35. Combined noise effects; S36. Effects of noise from stationary sources; S37. Abbreviations and terms used; References (Supplement).
