# Supplementary material for: WHO Environmental Noise Guidelines for the European Region: A Systematic Review on Environmental Noise and Annoyance
Source: Int J Environ Res Public Health. 2017 Dec 8;14(12):1539. doi: 10.3390/ijerph14121539 (PMC5750957; doi:10.3390/ijerph14121539)
Supplement: Supplementary file 1 [file ijerph-14-01539-s001.pdf]

Supplementary Materials:WHO Environmental Noise Guidelines for the European Region: A Systematic Review on Environmental Noise and Annoyance

Rainer Guski [1]*, Dirk Schreckenberg [2] and Rudolf Schuemer [3]

[1] Ruhr-University Bochum, Department of Psychology, 44801 Bochum, Germany
[2] ZEUS GmbH, 58093 Hagen, Germany; schreckenberg@zeusgmbh.de
[3] 58095 Hagen, Germany; ar-schuemer@t-online.de
* Correspondence: rainer.guski@ruhr-uni-bochum.de

S1. Author's questionnaires

1.1. Author's questionnaire for transportation noise

Effect-size data for the study published as: (please, insert the bibliographic data here)

No.	Question	Response
	Your Name:	
	Source (aircraft, road traffic, railway traffic, wind turbines, industry, …). If your study contains more than one source, please use a separate copy of this table for each source! Also, if your paper contains more than one survey, please use a separate copy of this table for each survey!	
1a	Dates of survey (month/year; e.g., 05/2001 - 09/2001)	
1b	Number of participants ("subjects"):	
2	Range of noise levels ($L_{Aeq,16h}$):	
3	Range of noise levels ($L_{Aeq,24h}$):	
4	Range of noise levels (L_{dn}):	
5	Range of noise levels (L_{den}):	
6	Classification of noise levels (continuous vs. discrete):	
6a	If discrete: number of steps:	
6b	If discrete: exact boundaries of steps:	
7	Pearson correlation $L_{Aeq,16h}$ vs. Annoyance-Scale 5-p verbal (raw scores):	r =<br>N =
8	Pearson correlation $L_{Aeq,16h}$ vs. Annoyance-Scale 11-p numeric (raw scores):	r =<br>N =
9	Pearson correlation $L_{Aeq,24h}$ vs. Annoyance-Scale 5-p verbal (raw scores):	r =<br>N =
10	Pearson correlation $L_{Aeq,24h}$ vs. Annoyance-Scale 11-p numeric (raw scores):	r =<br>N =
11	Pearson correlation L_{dn} vs. Annoyance-Scale 5-p verbal (raw scores):	r =<br>N =
12	Pearson correlation L_{dn} vs. Annoyance-Scale 11-p numeric (raw scores):	r =<br>N =
13	Pearson correlation L_{den} vs. Annoyance-Scale 5-p verbal (raw scores):	r =<br>N =
14	Pearson correlation L_{den} vs. Annoyance-Scale 11-p numeric (raw scores):	r =<br>N =
15	Definition of Highly Annoyed (HA, e.g., 60 or 72 or 75 % of Response Scale):	
16	Percent HA at 50 dB $L_{Aeq,16h}$ (grouped data):	Category boundaries =<br>%HA =<br>N =
17	Percent HA at 60 dB $L_{Aeq,16h}$ (grouped data):	Category boundaries =

No.	Question	Response
		%HA =<br>N =
18	Percent HA at 50 dB $L_{Aeq,24h}$ (grouped data):	Category boundaries =<br>%HA =<br>N =
19	Percent HA at 60 dB $L_{Aeq,24h}$ (grouped data):	Category boundaries =<br>%HA =<br>N =
20	Percent HA at 50 dB L_{dn} (grouped data):	Category boundaries =<br>%HA =<br>N =
21	Percent HA at 60 dB L_{dn} (grouped data):	Category boundaries =<br>%HA =<br>N =
22	Percent HA at 50 dB L_{den} (grouped data):	Category boundaries =<br>%HA =<br>N =
23	Percent HA at 60 dB L_{den} (grouped data):	Category boundaries =<br>%HA =<br>N =
24	Type of exposure-response relationship for %HA (e.g., linear regression; logistic regression: binary/ordinal; multilevel group regression; polynomial fit; …):	
25	Noise metric and confounders, if any, considered in the models, specified in 24.:	
26a	Equation/parameter values (e.g., B or exp(B) for logistic regression) for the model, specified in 24.: bivariate regression from annoyance on exposure; model without additional confounders	Equation/parameters; standard errors of parameters;<br>N=
26b	Equation/parameter values (e.g., B or exp(B) for logistic regression) for the model, specified in 24.: multivariate model with additional confounders	Equation/parameters; standard errors of parameters;<br>N=
27	Bivariate non-linear regression $L_{Aeq,16h}$ vs. %HA:	R2 =
28	Bivariate non-linear regression $L_{Aeq,24h}$ vs. %HA:	R2 =
29	Bivariate non-linear regression L_{dn} vs. %HA:	R2 =
30	Bivariate non-linear regression L_{den} vs. %HA:	R2 =
31	Multivar. non-linear regression (adj. for moderators/confounders) $L_{Aeq,16h}$ vs. %HA:	R2 =
32	Multivar. non-linear regression (adj. for moderators/confounders) $L_{Aeq,24h}$ vs. %HA:	R2 =
33	Multivar. non-linear regression (adj. for moderators/confounders) L_{dn} vs. %HA:	R2 =
34	Multivar. non-linear regression (adj. for moderators/confounders) L_{den} vs. %HA:	R2 =

1.2. Author's questionnaire for wind turbine noise

Effect-size data for the study published as: (please, insert the bibliographic data here)

No.	Question	Response
	Your Name:	
	Source (aircraft, road traffic, railway traffic, wind turbines, industry, …). If your study contains more than one source, please use a separate copy of this table for each source!<br>Also, if your paper contains more than one survey, please use a separate copy of this table for each survey!	
1a	Dates of survey (month/year; e.g., 05/2001 - 09/2001)	
1b	Number of participants ("subjects"):	
2	Range of noise levels ($L_{Aeq,16h}$):	
3	Range of noise levels ($L_{Aeq,24h}$):	
4	Range of noise levels (L_{dn}):	
5	Range of noise levels (L_{den}):	
6	Classification of noise levels (continuous vs. discrete):	
6a	If discrete: number of steps:	
6b	If discrete: exact boundaries of steps:	
7		
8		
9		
10		
11		
12		
13a	Point-biserial correlation L_{den} vs. Highly Annoyed indoors	Number of Scale categories included in the definition of "highly annoyed":<br>r =<br>N =
13b	Point-biserial correlation L_{den} vs. Highly Annoyed outdoors	Number of Scale categories included in the definition of "highly annoyed":<br>r =<br>N =
14		
15	Definition of Highly Annoyed (HA): a) which categories of the response scale were used for the definition of HA?; b) which percent of the response scale correspond to the cut-off? (e.g., 60 or 72 or 75 % of the response scale; according to the scale transformation into 0-100% by Miedema & Vos):	Categories:<br>Percent:
16		
17		
18		
19		

No.	Question	Response
20		
21		
22a	Percent HA at 42.5 dB L_{den} indoors (grouped original data):	Category boundaries =<br>%HA =<br>N =
22b	Percent HA at 42.5 dB L_{den} outdoors (grouped original data):	Category boundaries =<br>%HA =<br>N =
23a	Percent HA et 47.5 dB L_{den} indoors (grouped original data):	Category boundaries =<br>%HA =<br>N =
23b	Percent HA et 47.5 dB L_{den} outdoors (grouped original data):	Category boundaries =<br>%HA =<br>N =
24	Type of exposure-response relationship for %HA (e.g., linear regression; logistic regression: binary / ordinal; multilevel group regression; polynomial fit; …):	
25	Noise metric and confounders, if any, considered in the models, specified in 24.:	
26a	Equation/parameter values (e.g., B or exp(B) for logistic regression) for the indoor model, specified in 24.: bivariate regression from annoyance on exposure; model without additional confounders	Equation/parameters; standard errors of parameters;<br>N=
26b	Equation/parameter values (e.g. B or exp(B) for logistic regression) for the indoor model, specified in 24.: multivariate model with additional confounders	Equation/parameters; standard errors of parameters;<br>N=
26c	Equation/parameter values (e.g., B or exp(B) for logistic regression) for the outdoor model, specified in 24.: bivariate regression from annoyance on exposure; model without additional confounders	Equation/parameters; standard errors of parameters;<br>N=
26d	Equation/parameter values (e.g., B or exp(B) for logistic regression) for the outdoor model, specified in 24.: multivariate model with additional confounders	Equation/parameters; standard errors of parameters;<br>N=
27		
28		
29		
30	Bivariate non-linear regression L_{den} vs. %HA<br>(if possible, please indicate R2 according to Nagelkerke):	R2 =
31		
32		
33		
34	Multivar. non-linear regression (adj. for moderators/confounders) L_{den} vs. %HA<br>(if possible, please indicate R2 according to Nagelkerke):	R2 =

S2. Items used for rating the study quality

Topic area	Item	Topic	Information	Max. Rating
Overall survey design	1	Survey date	Year and months when the social survey information was obtained from respondents	1
	2a	Site location	The country & community(s) where the study sites were located	1
	2b	Unusual site characteristics	Any important, unusual characteristics of the study period or study sites (even if no unusual events or characteristics are to be reported)	1
	3	Site selection	The rationale and method for selecting study sites including all criteria that were explicitly used to select or exclude possible study sites	1
	4	Site size	The number of sites, areas, or locations where the social survey was conducted	1
	5	Study purpose	* The goals and purposes for conducting the study.<br>* The name of the organization that sponsored the survey.	1
Social survey sample	6	Sample selection	The general method for selecting respondents (probability, judgmental, etc.), the detailed procedures that were followed and any criteria that were followed to exclude some people in the study area (for example: age, gender, length of residence, etc.)<br>Quality rating: 0 = not reported, 1 = opportunity; 2 = stratified according to noise levels, or random design	2
	7a	Sample size (Issued)	A survey response rate and reference to the exact formula and operational definitions that were used to calculate the response rate<br>Quality rating: 0 = not reported; 1 = reported, but no standard formula; 2 = reported, and standard formula used	2
	7b	Selection bias	Methods used for assessing risk of selection bias (e.g., non-responder-analysis).<br>Quality rating: 0 = not reported; 1 = reported without non-responder analysis; 2 = non-responder analysis performed	2
Social survey data collection	8	Survey methods	The method used to obtain respondents' answers (Face-to-face interviews, telephone interviews, mail surveys, etc.). If interviewers are used, the training and qualifications of the interviewers are provided.	1
	9	Questionnaire wording	Exact wording of survey questions in the respondents' language and translated into language of the publication for annoyance questions and any other questions that were analyzed for the publication	1
	10	Precision of sample estimate	The number of respondents who provided answers that could be used in the analysis. The confidence intervals and results of significance tests for major results reported in the article	1
Nominal acoustical conditions (i.e., the common reference positions and	11	Noise source	The primary noise source studied (aircraft, road traffic, etc.) and any types of noise, types of operations or noise levels from that noise source that are not included in the reported noise exposure values	1
	12	Noise metrics	The complete, standard label for any noise metrics appearing in the article. If these metrics are not $L_{Aeq24hr}$, DENL and DNL, then an appropriate conversion rule should be given for estimating $L_{AeS24hr}$, DENL, and DNL from noise metrics used in the article.	1

conditions that the acoustical estimates represent)	13	Time period	The time period that the noise metric represents, in terms of hours of the day and number of days or months that the reported noise exposure values are assumed to represent	1
	14	Estimation measurement procedure	If the respondents' noise exposure is estimated, describe or cite the noise prediction model version. If the exposure is measured, describe the sound sampling, measurement and estimation protocols<br>Quality rating: 0 = not reported; 1 = occasional measurements at an unspecified place in the vicinity of participant's location; 2 = more than 6 days continuous measurements near the participants' location; 3 = noise levels from noise maps; 4 = calculated according to national/international standards (e.g., ISO 1996, ISO 20906)	4
	15	Reference position	The reference position for which the noise exposure values are normalized relative to the noise source and reflecting surfaces and a conversion rule for estimating the exposure at the noisiest façade of the respondents' dwelling excluding sound reflected from the façade	1
	16	Precision of noise estimate	Provide the best information available about accuracy of noise exposure estimates for the periods they nominally represent. For example, describe any unusual factors that affected the accuracy or ability to estimate long-term noise exposure.	1
Basic exposure-response analysis (if a study goal)	17	Exposure-response relationships	Present a tabulation of each degree of reaction for each category of noise exposure. A formula for estimating exposure-response relation would be equivalent	1
Explanatory variable analysis (if part of study objectives)	18	Non-noise variables' impacts on reactions (e.g., demographic, personal or community variables)	Present the size of each non-noise variable's effect controlled for noise level and in units or graphs that permit comparisons to the size of effects from noise exposure.<br>Conclusions should be reported for all variables, even if no statistically significant effect is found.<br>Compare the ability of noise level alone and of all explanatory variables together to explain response (e.g., correlation (r) and multiple correlation coefficient (R^2))	1

S3. List of papers included/excluded in the Evidence Review on Noise

1. Aircraft noise, papers included

Babisch, W., Houthuijs, D., Pershagen, G., Cadum, E., Katsouyanni, K., Velonakis, M., . for the HYENA-team. (2009). Annoyance due to aircraft noise has increased over the years - results of the HYENA study. Environment International, 35, 1169-1176. (aircraft data only)

Bartels, S., Müller, U. & Vogt, J. (2013). Predictors of aircraft noise annoyance: results of a telephone study. Paper presented at the Inter-Noise 2013, Innsbruck (A).

Breugelmans, O. R. P., van Wiechen, C. M. A. G., van Kamp, I., Heisterkamp, S. H. & Houthuijs, D. (2004). Gezondheid en beleving van de omgevingskwaliteit in de regio Schiphol 2002. Tussenrapportage Monitoring Gezondheidskundige Evaluatie Schiphol. Interim Report 630100001, Bilthoven (NL): RIVM.

Brink, M., Wirth, K., Thomann, G., Bauer, G. & Schierz, C. (2008). Annoyance responses to stable and changing aircraft noise exposure. Journal of the Acoustical Society of America, 124(5), 2930-2941.

Gelderblom, F. B., Gjestland, T. T., Granoien, I. L. N. & Taraldsen, G. (2014). *The Impact of Civil Versus Military Aircraft Noise on Noise Annoyance*. Paper presented at the Inter-Noise 2014, Melbourne, AUS.

Janssen, S. A. & Vos, H. (2009). A comparison of recent surveys to aircraft noise exposure-response relationships TNO Report (Vol. TNO-034-DTM-2009-01799, pp. 14).

Nguyen, T. L., Yano, T., Nguyen, H. Q., Nishimura, T., Fukushima, H., Sato, T., . . . Hashimoto, Y. (2011). Community response to aircraft noise in Ho Chi Minh City and Hanoi. Applied Acoustics, 72(11), 814-822.

Nguyen, T., Yano, T., Nguyen, H., Nishimura, T., Sato, T. & Morihara, T. (2012). *Community response to aircraft noise around three airports in Vietnam*. Paper presented at the Acoustics 2012, Hong Kong.

Sato, T. & Yano, T. (2011). *Effects of airplane and helicopter noise on people living around a small airport in Sapporo, Japan*. Paper presented at the 10th International Congress on Noise as a Public Health Problem (ICBEN), London (UK).

Schreckenberg, D. & Meis, M. (2007). Lärmbelästigung und Lebensqualität in der Bevölkerung am Frankfurter Flughafen. Lärmbekämpfung, 2(6), 225-233.

2. Aircraft noise, papers excluded

Ancona, C., Mataloni, F., Badaloni, C. & Forastiere, F. (2011). Aircraft noise and annoyance in the populations living near the Ciampino airport in Rome. Paper presented at the 10th International Congress on Noise as a Public Health Problem (ICBEN), London, UK. (**Reason: 3 noise levels only**)

Anderson, G. S., Rapoza, A. S., Fleming, G. G. & Miller, N. P. (2011). Aircraft noise dose-response relations for national parks. Noise Control Engineering Journal, 59(5), 519-540. (**Reason: No residents, only park visitors**)

Breugelmans, O. R. P., Stellato, R. K. & van Poll, R. (2007). Blootstelling-responsrelaties voor geluidhinder en slaapverstoring. Een analyse van nationale gegevens [Exposure-response relationship for noise annoyance and sleep disturbance. An analysis of national data] (pp. 54). Den Haag (NL): RIVM. (**Reason: very few persons exposed to noise**)

Brooker, P. (2008). Finding a good aircraft noise annoyance curve. Acoustics Bulletin, 33(4), 36-40. (**Reason: no new data**)

Brooker, P. (2009). Do people react more strongly to aircraft noise today than in the past? Applied Acoustics, 70(5), 747-752. (**Reason: no new data**)

Clark, C., Head, J. & Stansfeld, S. A. (2013). Longitudinal effects of aircraft noise exposure on children's health and cognition: A six-year follow-up of the UK RANCH cohort. Journal of Environmental Psychology, 35, 1-9. (**Reason: Response scale difficult to compare with ICBEN scale**)

Elmehdi, H. M. (2012). Relationship between civil aircraft noise and community annoyance near Dubai International Airport. Acoustical Science and Technology, 33(1), 6-10. (**Reason: Insufficient data**)

Fidell, S., Pearsons, K., Silvati, L. & Sneddon, M. (2002). Relationship between low-frequency aircraft noise and annoyance due to rattle and vibration. Journal of the Acoustical Society of America, 111(4), 1743-1750. (**Reason: No comparable acoustic data**)

Fidell, S. & Silvati, L. (2004). Parsimonious alternative to regression analysis for characterizing prevalence rates of aircraft noise annoyance. Noise Control Engineering Journal, 5(2), 56-68.(**Reason: Few new data**)

Fidell, S., Silvati, L. & Haboly, E. (2002). Social survey of community response to a step change in aircraft noise exposure. Journal of the Acoustical Society of America, 111(1), 200-209. (**Reason: Change study**)

Houthuijs, D., Ameling, C., van Acker, M., Bouwman-Notenboom, A.J., ten Brinke, J., van den Brink, M., Dijkshoorn, H., Heemskerk, M., van de Laar, A., Mulder, M., Rozema, B., Schütz, F., Verhagen. C., Marra, M., Breugelmans, O., Swart, W., van de Kassteele, J., van den Brink, C.L., van Wiechen, C. (2011). Mapping of severe annoyance due to aircraft noise. Paper presented at the 10th International Congress on Noise as a Public Health Problem (ICBEN) 2011, London, UK. (**Reason: No individual data**)

Janssen, S. A. & Vos, H. (2011). Dose-response relationship between DNL and aircraft noise annoyance: Contribution of TNO (Vol. Report TNO-060-UT-2011-00207). Utrecht (NL): TNO. (**Reason: Data contained in Janssen & Vos 2009**)

Janssen, S. A., Vos, H., Van Kempen, E., Breugelmans, O. & Miedema, H. M. E. (2011). Trends in aircraft noise annoyance: The role of study and sample characteristics. Journal of the Acoustical Society of America, 129(4), 1953-1962. (**Reason: Data contained in Janssen & Vos 2009**)

Kroesen, M., Molin, E. J. E., Miedema, H. M. E., Vos, H., Janssen, S. A. & van Wee, B. (2010). Estimation of the effects of aircraft noise on residential satisfaction. Transportation Research Part D: Transport and Environment, 15(3), 144-153. (**Reason: outcome not comparable**)

Krog, N. H. & Engdahl, B. (2004). Annoyance with aircraft noise in local recreational areas, contingent on changes in exposure and other context variables. Journal of the Acoustical Society of America, 116(1), 323-333. (**Reason: no residents**)

Lim, C., Kim, J., Hong, J., Lee, S. & Lee, S. (2007). The relationship between civil aircraft noise and community annoyance in Korea. Journal of Sound & Vibration, 299(3), 575-586. (**Reason: Insufficient data**)

Lim, C., Kim, J., Hong, J. & Lee, S. (2008). Effect of background noise levels on community annoyance from aircraft noise. Journal of the Acoustical Society of America, 123(2), 766-771. (**Reason: Data contained in Lim et al 2007**)

Miedema, H. M. E. & Oudshoorn, C. G. (2001). Annoyance from transportation noise: Relationships with exposure Metrics DNL and DENL and their confidence intervals. Environmental Health Perspectives, 109, 409-416. (**Reason: mean age group of data: 1978 (air/road), no data after 1993**)

Morinaga, M., Tsukioka, H., Yamada, I. & Matsui, T. (2011). The effect of regional living environmental improvement on community response to aircraft noise. Paper presented at the 10th International Congress on Noise as a Public Health Problem (ICBEN), London (UK). (**Reason: military airport**)

MVA-Consultancy. (2007). ANASE: Attitudes to Noise from Aviation Sources in England. Final Report. Woking / Norwich (UK): Queen's Printer and Controller of HMSO. (**Reason: Insufficient individual data**)

Nguyen, T. L., Yano, T., Nguyenhuy, Q., Nishimura, T., Sato, T., Morihara, T. & Hashimoto, Y. (2011). Dose-response relationships for aircraft noise annoyance in Ho Chi Minh City and Hanoi. Paper presented at the 10th International Congress on Noise as a Public Health Problem (ICBEN), London (UK). (**Reason: Data contained in Nguyen et al. 2011**)

Quehl, J. & Basner, M. (2006). Annoyance from nocturnal aircraft noise exposure: Laboratory and field-specific dose–response curves. Journal of Environmental Psychology, 26(2), 127-140. (**Reason: Acoustics and annoyance variable not comparable**)

Seabi, J. (2013). An epidemiological prospective study of children's health and annoyance reactions to aircraft noise exposure in South Africa. Int J Environ Res Public Health, 10(7), 2760-2777. (**Reason: no comparable data**)

Schreckenberg, D., Meis, M., Kahl, C., Peschel, C. & Eikmann, T. (2010). Aircraft Noise and Quality of Life around Frankfurt Airport. International Journal of Environmental Research and Public Health, 7, 3382-3405. (**Reason: Data contained in Schreckenberg & Meis 2007**)

Stansfeld, S. A., Berglund, B., Clark, C., Lopez-Barrio, I., Fischer, P., Öhrström, E., Haines, M.M., Head, J., Hygge, S., van Kamp, I. & Berry, B. F. (2005). Aircraft and road traffic noise and children's cognition and health: a cross-national study. The Lancet, 365(9475), 1942-1949. (**Reason: included in v.Kempen et al. 2009**)

Van Kamp, I., Job, R. F., Hatfield, J., Stellato, R. K. & Stansfeld, S. A. (2004). The role of noise sensitivity in the noise-response relation: a comparison of three international airport studies. Journal of the Acoustical Society of America, 116, 3471-3479. (**Reason: No common acoustic descriptor**)

Van Kempen, E. & Van Kamp, I. (2005). Annoyance from air traffic noise. Possible trends in exposure-response relationships (Vol. RIVM Report 01/2005). Bilthoven (NL): RIVM. (**Reason: no common exp.-resp. function**)

van Kempen, E. E. M. M., van Kamp, I., Stellato, R. K., Houthuijs, D. J. M. & Fischer, P. H. (2005). Het effect van geluid van vlieg- en wegverkeer op cognitie, hinderbeleving en de bloeddruk van basisschoolkinderen. [The effect of aircraft and road traffic noise on the cognitive performance, annoyance and blood pressure of primary school children] (Vol. RIVM Report 441520021, pp. 100): RIVM. (**Reason: Data contained in van Kempen et al. 2009**)

Wirth, K., Brink, M. & Schierz, C. (2004). Lärmstudie 2000: Fluglärmbelästigung um den Flughafen Zürich-Kloten. Zeitschrift für Lärmbekämpfung, 51, 48-56. (**Reason: data contained in Brink et al. 2008**)

3. Road traffic noise, papers included

Babisch, W., Houthuijs, D., Pershagen, G., Cadum, E., Katsouyanni, K., Velonakis, M., . . . for the HYENA-team. (2009). Annoyance due to aircraft noise has increased over the years - results of the HYENA study. *Environment International, 35*, 1169-1176. (road data only)

Brink, M. (2013). *Annoyance Assessment in Postal Surveys Using the 5-point and 11-point ICBEN Scales: Effects of Sale and Question Arrangement*. Paper presented at the Inter-Noise 2013, Innsbruck (A).

Brown, A. L., Lam, K. C., van Kamp, I. & Yeung, M. K. L. (2014). Urban road traffic noise. Exposure and human response in a dense, high-rise city in Asia. Paper presented at the ICBEN 2014, Nara (Jap).

Brown, A. L., Lam, K. C. & Van Kamp, I. (2015). Quantification of the exposure and effects of road traffic noise in a dense Asian city: a comparison with western cities. *Environmental Health, 2015*, 14-22. doi: 10.1186/s12940-015-0009-8.

Champelovier, P., Cremezi-Charlet, C. & Lambert, J. (2003). Evaluation de la gêne due à l'exposition combinée aux bruits routier et ferroviaire (Vol. Report 242). Lyon: INRETS. (road data only)

Heimann, D., de Franceschi, M., Emeis, S., Lercher, P. & Seibert, P. (Eds. 2007). Air Pollution, Traffic Noise and Related Health Effects in the Alpine Space - A Guide for Authorities and Consulters. ALPNAP comprehensive report. Trento (I): Universita degli Studi di Trento, Dipartimento di Ingegneria Civile e Ambientale. [road traffic data only]

Medizinische Universität Innsbruck. (2008). Galleria di Base del Brennero - Brenner Baistunnel - Sozioökonomie (Public health) - Zusammenfassender Bericht (pp. 815). Bolzano (I) / Innsbruck (A): Medizinische Universität Innsbruck & Brenner Basistunnel BBT SE. (Authors: Lercher et al.; road data only)

Pierrette, M., Marquis-Favre, C., Morel, J., Rioux, L., Vallet, M., Viollon, S. & Moch, A. (2012). Noise annoyance from industrial and road traffic combined noises: A survey and a total annoyance model comparison. *Journal of Environmental Psychology, 32*(2), 178-186. (road data only)

Sato, T., Yano, T., Björkman, M. & Rylander, R. (2002). Comparison of community response to road traffic noise in Japan and Sweden - Part I: Outline of surveys and dose response relationships. Journal of Sound and Vibration, 250, 161-167.

Shimoyama, K., Nguyen, T. L., Yano, T. & Morihara, T. (2014). *Social surveys on community response to road traffic in five cities in Vietnam*. Paper presented at the Inter-Noise 2014, Melbourne (AUS).

4. Road traffic noise, papers excluded

Ali, S. A. (2004). Investigation of the dose–response relationship for road traffic noise in Assiut, Egypt. Applied Acoustics, 65(11), 1113-1120. (**Reason: Insufficient data**)

Amundsen, A. H., Klæboe, R. & Aasvang, G. M. (2013). Long-term effects of noise reduction measures on noise annoyance and sleep disturbance: The Norwegian facade insulation study. Journal of the Acoustical Society of America, 133(6), 3921-3928. (**Reason**: **Change study**)

Babisch, W., Schulz, C., Seiwert, M. & Conrad, A. (2012). Noise annoyance as reported by 8- to 14-year-old children. Environment and Behavior, 44(1), 68-86. (**Reason: Exposure variable (15 min L_{Aeq}) not comparable**)

Banerjee, D. (2013). Road traffic noise exposure and annoyance: A cross-sectional study among adult Indian population. Noise & Health, 15(66), 342--346. (**Reason: Outcome not comparable**)

Birk, M., Ivina, O., von Klot, S., Babisch, W. & Heinrich, J. (2011). Road traffic noise: self-reported noise annoyance versus GIS modelled road traffic noise exposure. Journal of Environmental Monitoring, 13(11), 3237-3245. (**Reason: Outcome not comparable**)

Bluhm, G., Nordling, E. & Berglind, N. (2004). Road Traffic Noise and Annoyance - An increasing Environmental Health Problem. Noise & Health, 6, 43-49. (**Reason: Outcome not comparable**)

de Kluizenaar, Y., Janssen, S. A., Vos, H., Salomons, E. M., Zhou, H. & v. d. Berg, F. (2013). Road traffic noise and annoyance: a quantification of the effect of quiet side exposure at dwellings. Int J Environ Res Public Health, 10(6), 2258-2270. (**Reason: Outcome not comparable**)

Dusseldorp, A., Houthuijs, D., van Overveld, A., van Kamp, I. & Marra, M. (2011). Handreiking geluidhinder wegverkeer [Guideline Noise Annoyance] (pp. 67): RIVM. (**Reason: Insufficient data**)

Gidlöf-Gunnarsson, A., Öhrström, E. & Forssén, J. (2012b). The effect of creating a quiet side on annoyance and sleep disturbances due to road traffic noise. Paper presented at the Inter-Noise 2012, New York City (USA). (**Reason: Change study**)

Heinonen-Guzejev, M., Vuorinen, H. S., Kaprio, J., Heikklikä, K., Mussalo-Rauhamaa, H. & Koskenvuo, M. (2000). Self-report of transportation noise exposure, annoyance and noise sensitivity in relation to noise map information. Journal of Sound and Vibration, 234, 191-206. (**Reason: Outcome not comparable**)

Jakovljevic, B., Paunovic, K. & Belojevic, G. (2009). Road-traffic noise and factors influencing noise annoyance in an urban population. Environment International, 35(3), 552-556. (**Reason: Outcome not comparable**)

Klaeboe, R., Amundsen, A. H., Fyhri, A. & Solberg, S. (2004). Road traffic noise – the relationship between noise exposure and noise annoyance in Norway. Applied Acoustics, 65(9), 893-912. (**Reason: Change study**)

Lercher, P., Boeckstael, A., Coensel, B. D., DeKoninck, L. & Botteldooren, D. (2012). The application of a notice-event model to improve classical exposure-annoyance estimation. Paper presented at the Acoustics 2012, Hong-Kong. (**Reason: Data included in Lercher et al. 2008**)

Michaud, D. S., Keith, S. E. & McMurchy, D. (2008). Annoyance and disturbance of daily activities from road traffic noise in Canada. Journal of the Acoustical Society of America, 123(2), 784-792. (**Reason: No exposure-response data**)

Miedema, H. M. E. & Oudshoorn, C. G. (2001). Annoyance from transportation noise: Relationships with exposure Metrics DNL and DENL and their confidence intervals. Environmental Health Perspectives, 109, 409-416. (**Reason: Mean age group of data: 1978 (air/road), no data after 1993**)

Morihara, T., Sato, T. & Yano, T. (2004). Comparison of dose–response relationships between railway and road traffic noises: the moderating effect of distance. Journal of Sound & Vibration, 277(3), 559-565. (**Reason: Insufficient data**)

Nilsson, M. E. & Berglund, B. (2006). Noise annoyance and activity disturbance before and after the erection of a roadside noise barrier. Journal of the Acoustical Society of America, 119(4), 2178-2188. (**Reason: No exposure-response data; change study**)

Phan, H. Y. T., Yano, T., Phan, H. A. T., Nishimura, T., Sato, T. & Hashimoto, Y. (2010). Community responses to road traffic noise in Hanoi and Ho Chi Minh City. Applied Acoustics, 71(2), 107-114. (**Reason: Data included in Shimoyama et al. 2014**)

Renew, W. (2000). Responses To Road Traffic Noise In Brisbane. Paper presented at the Inter-Noise 2000, Nice (F). (**Reason: Outcome not comparable**)

5. Railway noise, papers included

Champelovier, P., Cremezi-Charlet, C. & Lambert, J. (2003). Evaluation de la gêne due à l'exposition combinée aux bruits routier et ferroviaire (Vol. Report 242). Lyon: INRETS. (for railway data)

Gidlöf-Gunnarsson, A., Ögren, M., Jerson, T. & Öhrström, E. (2012a). Railway noise annoyance and the importance of number of trains, ground vibration, and building situational factors. Noise & Health, 14(59), 190-201.

Lercher, P., de Greve, B., Botteldooren, D., Dekoninck, L., Oettl, D., Uhrner, U. & Rüdisser, J. (2008). *Health effects and major co-determinants associated with rail and road noise exposure along transalpine traffic corridors*. Paper presented at the 9th International Congress on Noise as a Public Health Problem (ICBEN), Foxwoods (USA, CT).

Sato, T., Yano, T. & Morihara, T. (2004). Community Response to Noise from Shinkansen in Comparison with Ordinary Railways: A Survey in Kyushu, Japan. Paper presented at the International Congress on Acoustics ICA 2004.

Schreckenberg, D. (2013). Exposure-response relationship for railway noise annoyance in the middle Rhine Valley. Paper presented at the Inter-Noise 2013, Innsbruck (A).

Yano, T., Morihara, T. & Sato, T. (2005). Community response to Shinkansen noise and vibration: a survey in areas along the Sanyo Shinkansen Line. Paper presented at the Forum Acusticum, Budapest (H).

Yokoshima, S., Morihara, T., Ota, A. & Tamura, A. (2008). Reanalysis of dose-response curves of Shinkansen railway noise. Paper presented at the 9th International Congress on Noise as a Public Health Problem (ICBEN), Foxwoods (USA, CT).

6. Railway noise, papers excluded

Aasvang, G. M., Engdahl, B. & Rothschild, K. (2007). Annoyance and self-reported sleep disturbances due to structurally radiated noise from railway tunnels. Applied Acoustics, 68(9), 970-981. (**Reason: Acoustics not comparable**)

Breugelmans, O. R. P., Stellato, R. K. & van Poll, R. (2007). Blootstelling-responsrelaties voor geluidhinder en slaapverstoring. Een analyse van nationale gegevens [Exposure-response relationship for noise annoyance and sleep disturbance. An analysis of national data] (pp. 54). Den Haag (NL): RIVM. (**Reason: Very few persons exposed**)

Chen, X., Tang, F., Huang, Z. & Wang, G. (2007). High-speed maglev noise impacts on residents: A case study in Shanghai. Transportation Research: Part D, 12(6), 437-448. (**Reason: Acoustics not comparable**)

Elmenhorst, E.-M., Pennig, S., Rolny, V., Quehl, J., Mueller, U., Maaß, H. & Basner, M. (2012). Examining nocturnal railway noise and aircraft noise in the field: Sleep, psychomotor performance, and annoyance Science of the Total Environment, 424, 48-56. (**Reason: Outcome and acoustics not comparable**)

Heinonen-Guzejev, M., Vuorinen, H. S., Kaprio, J., Heikklikä, K., Mussalo-Rauhamaa, H. & Koskenvuo, M. (2000). Self-report of transportation noise exposure, annoyance and noise sensitivity in relation to noise map information. Journal of Sound and Vibration, 234, 191-206. (**Reason: Outcome not comparable**)

Koziel, Z. (2011). Exposure-response relationships from railway noise in the presence of vibration. (MSc), University of Salford. (**Reason: Insufficient data**)

Lam, K.-C. & Au, W.-H. (2008). Human Response to a Step Change in Noise Exposure Following the Opening of a New Railway Extension in Hong Kong. Acta Acustica united with Acustica, 94, 553-562. (**Reason: Outcome not comparable**)

Lim, C., Kim, J., Hong, J. & Lee, S. (2006). The relationship between railway noise and community annoyance in Korea. The Journal of the Acoustical Society of America, 120(4), 2037-2042. (**Reason: Insufficient data**)

Miedema, H. M. E. & Oudshoorn, C. G. (2001). Annoyance from transportation noise: Relationships with exposure Metrics DNL and DENL and their confidence intervals. Environmental Health Perspectives, 109, 409-416. (**Reason: Mean age group of rail data: 1981, no data after 1993**)

Morihara, T., Sato, T. & Yano, T. (2004). Comparison of dose–response relationships between railway and road traffic noises: the moderating effect of distance. Journal of Sound & Vibration, 277(3), 559-565. (**Reason: Insufficient data**)

Oka, S., Murakami, Y., Tetsuya, H. & Yano, T. (2013). Community response to a step change in railway noise and vibration exposures by the opening of a new Shinkansen Line. Paper presented at the Inter-Noise 2013, Innsbruck (A). (**Reason: Change study**)

Pennig, S., Quehl, J., Müller, U., Elmenhorst, E.-M., Rolny, V., Maaß, H. & Basner, M. (2011). Effects of nocturnal railway noise on annoyance: Dose-response relationships from a field study in comparison to nocturnal aircraft noise annoyance. Paper presented at the 10th International Congress on Noise as a Public Health Problem (ICBEN), London (UK). (**Reason: Exposure-response data only for L_{night}**)

Pennig, S., Quehl, J., Mueller, U., Rolny, V., Maass, H., Basner, M. & Elmenhorst, E. M. (2012). Annoyance and self-reported sleep disturbance due to night-time railway noise examined in the field. Journal of the Acoustical Society of America, 132(5), 109-117. (**Reason: exposure-response data only for L_{night}**)

Schreckenberg, D., Moehler, U., Liepert, M. & Schuemer, R. (2013). The impact of railway grinding on noise levels and resident's noise responses -- Part 2: The role of information. Paper presented at the Inter-Noise 2013, Innsbruck (A). (**Reason: No exposure-response data**)

7. Wind turbine noise, papers included

Janssen, S. A., Vos, H., Eisses, A. R. & Pedersen, E. (2011). A comparison between exposure-response relationships for wind turbine annoyance and annoyance due to other noise sources. Journal of the Acoustical Society of America, 130(6), 3746-3753.

Kuwano, S., Yano, T., Kageyama, T., Sueoka, S. & Tachibanae, H. (2014). Social survey on wind turbine noise in Japan. Noise Control Engineering Journal, 62(6), 503-520.

8. Wind turbine noise, papers excluded

Bakker, R. H., Pedersen, E., Berg, G. P. v. d., Stewart, R. E., Lok, W. & Bouma, J. (2012). Impact of wind turbine sound on annoyance, self-reported sleep disturbance and psychological distress. Science of The Total Environment, 425, 42-51. (**Reason: data included in Janssen et al 2011**)

Magari, S. R., Smith, C. E., Schiff, M. & Rohr, A. C. (2014). Evaluation of community response to wind turbine-related noise in Western New York State. Noise & Health, 16(71), 228-239. (**Reason: No exposure response data**)

Pawlaczyk-Luszczynska, M., Dudarewicz, A., Zaborowski, K., Zamojska, M. & Waszkowska, M. (2013). Assessment of annoyance due to wind turbine noise. Paper presented at the ICA 2013, Montreal (CDN). (**Reason: Response scale and HA definition not comparable**)

Pawlaczyk-Luszczynska, M., Dudarewicz, A. & Zaborowski, K. e. a. (2014). Annoyance Related to Wind Turbine Noise. Archives of Acoustics, 39(1), 89-102. (**Reason: Response scale and HA definition not comparable**)

Pedersen, E. & Persson-Waye, K. (2004). Perception and annoyance due to wind turbine noise--a dose-response relationship. Journal of the Acoustical Society of America, 116(6), 3460-3470. (**Reason: Data included in Janssen et al., 2011**)

Pedersen, E. & Persson Waye, K. (2007). Wind turbine noise, annoyance and self-reported health and well-being in different living environments. Occupational and Environmental Medicine, 64, 480-486. (**Reason: Data included in Janssen et al., 2011**)

Pedersen, E., van den Berg, F., Bakker, R. & Bouma, J. (2009). Response to noise from modern wind farms in The Netherlands. Journal of the Acoustical Society of America, 126(2), 634-643. (**Reason: Data included in Janssen et al., 2011**)

Van den Berg, F., Pedersen, E., Bouma, J. & Bakker, R. (2008). WINDFARM perception. Visual and acoustic impact of wind turbine farms on residents. Final report (Vol. FP6-2005-Science-and-Society-20, Project no. 044628). Groningen (NL): University of Groningen. (**Reason: Data included in Janssen et al., 2011**)

Yano T., Kuwano S., Kageyama T. , Sueoka S., Tachibana H. (2013). Dose-response relationships for wind turbine noise in Japan. Proceedings of Inter-Noise 2013, Innsbruck/Austria. (**Reason: Data included in Kuwano et al., 2014**)

9. Combined transportation noise, papers included

Note: Some of the following papers provide exposure-response data for single noise sources. These data are considered in the respective quantitative sections of the report.

Champelovier, P., Cremezi-Charlet, C. & Lambert, J. (2003). Evaluation de la gêne due à l'exposition combinée aux bruits routier et ferroviaire (Report 242). Lyon: INRETS. [Sources: road traffic, rail; combined]

Lercher, P., Botteldooren, D., de Greve, B., Dekoninck, L. & Rüdisser, J. (2007). *The effects of noise from combined traffic sources on annoyance: the case of interactions between rail and road noise*. Paper presented at the Inter-Noise 2007, Istanbul, TR. [Sources: railway, road traffic; combined]

Nguyen, T. L., Nguyen, H. Q., Yano, T., Nishimura, T., Sato, T., Morihara, T. & Hashimoto, Y. (2012). Comparison of models to predict annoyance from combined noise in Ho Chi Minh City and Hanoi. Applied Acoustics, 73(9), 952-959. [Sources: aircraft + road combined]

Pierrette, M., Marquis-Favre, C., Morel, J., Rioux, L., Vallet, M., Viollon, S. & Moch, A. (2012). Noise annoyance from industrial and road traffic combined noises: A survey and a total annoyance model comparison. *Journal of Environmental Psychology, 32*(2), 178-186. [Sources: road traffic, industrial noise; combined]

10. Combined transportation noise, papers excluded

Brink, M. & Lercher, P. (2007). *The effects of noise from combined traffic sources on annoyance: the interaction between aircraft and road traffic noise*. Paper presented at the Inter-Noise 2007, Istanbul (TR). (**Reason: Insufficient data**)

Cremezi, C., Gautier, P. E., Lambert, J. & Champelovier, P. (2001). Annoyance due to combined noise sources - advanced results. Paper presented at the 17. International Congress on Acoustics (ICA), Rome (I). (**Reason: Data contained in Champelovier et al., 2003**)

Di, G., Liu, X., Lin, Q., Zheng, Y. & He, L. (2012). The relationship between urban combined traffic noise and annoyance: An investigation in Dalian, north of China. Science of the Total Environment, 432(0), 189-194. (**Reason: Unspecific annoyance question + insufficient data**)

Joncour, S., Cailhau, D., Gautier, P. E., Champelovier, P. & Lambert, J. (2000). Annoyance due to combined noise sources. Paper presented at the Inter-Noise 2000, Nice (F). (**Reason: Data contained in Champelovier et al., 2003)**

Lam, K.-C., Chan, P.-K., Chan, T.-C., Aua, W.-H. & Hui, W.-C. (2009). Annoyance response to mixed transportation noise in Hong Kong. Applied Acoustics, 70(1), 1–10. (**Reason: Insufficient data**)

Öhrström, E., Barregård, K., Andersson, E., Skånberg, A., Svensson, H. & Ängerheim, P. (2007). Annoyance due to single and combined sound exposure from railway and road traffic. Journal of the Acoustical Society of America, 122(5), 2642-2652. (**Reason: Insufficient data**)

Sato, T. & Yano, T. (2011). Effects of airplane and helicopter noise on people living around a small airport in Sapporo, Japan. Paper presented at the 10th International Congress on Noise as a Public Health Problem (ICBEN), London (UK). (**Reason: Insufficient data**)

11. Stationary noise sources, paper included

Miedema, H. M. E. & Vos, H. (2004). Noise annoyance from stationary sources: Relationships with exposure metric day evening night level (DENL) and their confidence intervals. Journal of the Acoustical Society of America, 116, 334-343. [Sources: industrial, seasonal, and shunting noise]

S4. Grading the quality of evidence for the exposure-response relation of %HA by aircraft noise

The confidence in the evidence with respect to exposure-response relations between aircraft noise levels and the percentage of high aircraft noise annoyance may be decreased for several reasons, including

Study limitations: for ethical reasons, randomized controlled trials are not feasible, and research on the effects of environmental noise on residents in the vicinity of airports is confined to observational studies. These have been done by means of diverse methods of participant selection, survey type, and noise exposure assessment. We have taken the study limitations into account by grading the quality of each study selected, and using it in heterogeneity analyses, as far as possible.

Inconsistency of results: The exposure-response relations shown in Figure 2 in section 3.1.2 of the main text reveal wide scatter between the 12 studies used here. The amount of scatter could not be analyzed properly, but it may partially be due to the mixture of studies from airports in "low-rate" and "high-rate" change situations (see 3.1.4). The scatter leads to a downgrading of the quality of evidence.

Indirectness of evidence: the GRADE system distinguishes between two types of indirectness: the first is related to experimental interventions – which are not applicable here and have been replaced by exposure descriptions -, the second type "includes differences between the population, intervention, comparator to the intervention, and outcome of interest, and those included in the relevant studies" [1, p. 997]. In sum, Population, Exposure, Comparator, and Outcomes (PECO) in the studies selected here are judged to be comparable:

- **Populations**: even though our sample of studies comprises different methods of participant selection, all studies include participants exposed to everyday aircraft noise, and we do not see relevant differences between the population and the sample of participants included in the studies, except with respect to the age range: none of the studies includes children – they would need special types of annoyance questions. The typical age range for noise surveys starts at 18 years and goes up to more than 80 years. Exceptions in our sample are the six studies done in the context of the HYENA project [2]: due to the primary goal of the project – to study the relation between hypertension and noise – the age range is 45-70 years.
- **Exposure**: all studies analyzed here include aircraft noise, described by L_{den}
- **Comparator**: all studies use comparable annoyance questions, comparable response scales, and the same criterion of being highly annoyed (≥73% of the response scale length).

Outcomes are comparable, too (see Comparator).

Imprecision: This dimension is relevant mainly to small samples. In contrast, the samples of the studies reported here are between about 300 and nearly 6,000 in size.

Publication bias: Most of the studies selected are journal publications, a small fraction is due to conference papers. This distribution may be prone to publication bias, because authors and journals may tend to publish large effects more than small effects. On the other hand, the funnel plot of correlations (not shown here) shows a distribution of studies which is not in line with the expectation associated with publication bias: the largest effect sizes are seen in studies of medium precision – not in studies with low precision. However, it should be remembered that six of the studies in the WHO data set include residents aged 45-70 years only – which might have contributed to an increase of annoyance (see main text 3.1.3).

With respect to the exposure-response relations reported here, it should be noted that all studies included here show a statistically significant correlation between noise levels and raw scores (see 3.1.5), and they all show a clear increase of %HA with increasing noise levels as well. However, the methods used in order to show the relation between L_{den} and %HA vary between studies (e.g., some used binary logistic regression, some used a polynomial regression model, and one study used a multilevel grouped regression), and we aggregated the resulting estimated data. In addition, only a

minority of studies reported statistical information about the effect size (e.g., Nagelkerke's R^2). Due to these restrictions, an assessment in terms of GRADE both of the exposure-response relation itself, and with respect to the size of the effect of noise levels on %HA was not possible.

In sum, we are moderately confident in the evidence with respect to exposure-response relations between aircraft noise levels and percentage of high aircraft noise annoyance, and like to assign the grade "moderate quality" – see Table S1.

Table S1. GRADE summary of findings for the quality of evidence related to aircraft noise and percent of high annoyance. Health outcome based on exposure-response relations, 12 studies.

Domains	Criterion	Assessment	Grading
Start Level	Study design: cross-sectional = high quality	High quality	High quality
1. Study Limitations	Quality of majority of studies (risk of bias)	High quality of majority of studies	No downgrade
2. Inconsistency	Conflicting results	High between study scatter	Downgrade one level
3. Directness	Direct comparison; same PECO	Same PECO	No downgrade
4. Precision	Small sample sizes OR Low numbers of events (HA) OR Wide confidence intervals	Large study samples	No downgrade
5. Publication Bias	Funnel plot indicates	No publication bias	No downgrade
Overall Judgment			Moderate quality
6. Exposure-response	Statistically significant trend %HA vs. L_{den}	Not assessable	
7. Magnitude of effect	Fit of logistic regression	Not assessable	
8. Confounding adjusted	Effect in spite of confounding working towards the nil	No adjustments	No upgrade
Overall Judgment			Moderate quality

PECO = Population, Exposure, Comparator, Outcome. See explanations in 3.1.3 above.

S5. Correlations between aircraft noise annoyance raw scores and weighted vs. unweighted 24-h-noise levels

In order to show comparative correlational data, Table S2 depicts correlation coefficients between annoyance raw scores and L_{Aeq} values from 14 aircraft noise studies both for the correlation with $L_{Aeq,24h}$ and for the correlation with L_{den} or L_{dn} – the latter is used in the study at Cologne Airport.

Table S2. Pearson correlations between aircraft noise annoyance raw scores and $L_{Aeq,24h}$ vs. L_{den} or L_{dn}. The coefficients are not weighted according to sample size.

**Study** (see S3 for references)	**Location**	**Correlation with $L_{Aeq,24h}$**	**Correlation with L_{den} or L_{dn}**	**Difference L_{den} - $L_{Aeq,24h}$**
Babisch-Hyena	D (Tegel)	0.587	0.586	-0.001
Babisch-Hyena	GB (Heathrow)	0.469	0.469	0.000
Babisch-Hyena	GR (Athens)	0.517	0.492	-0.025
Babisch-Hyena	I (Milano-Malpensa)	0.735	0.735	0.000
Babisch-Hyena	NL (Schiphol)	0.331	0.320	-0.011
Babisch-Hyena	SWE (Arlanda)	0.513	0.517	+0.004
Brink 2008	Zurich before 2001	0.331	0.325	-0.006
Bartels et al. 2013	Cologne/Bonn	0.414	0.410*	-0.004
Gelderblom et al. 2014	Trondheim	0.360	0.370	+0.010
Schreckenberg & Meis 2007	Fraport	0.434	0.418	-0.016
Nguyen 2012	Da Nang	0.180	0.253	+0.073
Nguyen 2011	Hanoi	0.363	0.320	-0.043
Nguyen 2011	Ho Chi Minh City	0.556	0.565	+0.009
Sato & Yano 2011	Airplanes	0.213	0.214	+0.001
Unweighted average, calculated via Fisher's z'		0.429	0.428	-.001

*This correlation relates to L_{dn}

It can be seen from Table S2 that the differences in the direction and amount of correlations between aircraft noise annoyance raw scores and $L_{Aeq,24h}$ or L_{den} in the aircraft noise studies used here are rather small. The largest difference relates to a study at Da Nang airport in Vietnam, and the reasons for this difference are unclear. The restricted range of noise levels (48.9 - 60.3 dB $L_{Aeq,24h}$) may be a problem, but it is shared with other studies in our sample.

S6. An analysis to detect a bias in reported correlations between aircraft noise annoyance raw scores and aircraft noise

So-called "funnel plots" are often used in order to guess the **presence of a bias** from the distribution of effect sizes in relation to a scale indicating the precision of the effect estimation. In former times, the funnel plot had the effect size on the X axis and the sample size or variance on the Y axis. Today, the standard error of the effect size is a common variable at the Y axis. "Large studies appear toward the top of the graph and generally cluster around the mean effect size. Smaller studies appear toward the bottom of the graph, and (since smaller studies have more sampling error variation in effect sizes) tend to be spread across a broad range of values. This pattern resembles a funnel, hence the plot's name […]. The use of the standard error (rather than sample size or variance) on the Y axis has the advantage of spreading out the points on the *bottom half* of the scale, where the smaller studies are plotted. This could make it easier to identify asymmetry" [3, p.283].

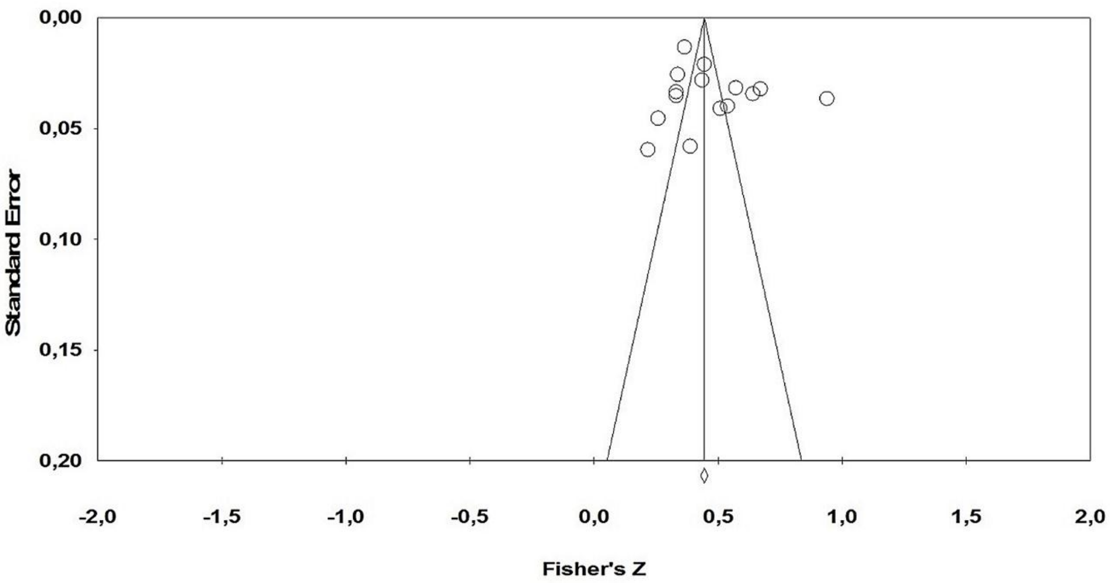

Figure S1. Funnel plot of the relation between the correlational aircraft noise annoyance effect (related to L_{den}) and the standard error of the effect in the sample of 15 studies. "Fisher's Z" means Fisher's z', and indicates the direction and strength of the (correlational) noise effect, and "Standard Error" indicates the imprecision of the noise effect estimation – a larger standard error indicates lower precision. The outer point to the right is due to the high correlation at Malpensa Airport.

In the *absence of bias*, the studies will be distributed *symmetrically* around the mean effect size, since the sampling error is random. In the *presence of bias,* the bottom of the plot will show a larger concentration of studies on one side of the mean than on the other. This reflects the idea that smaller studies (which appear toward the bottom) are more likely to be published if they have larger than average effects, since these studies are likelier to be statistically significant [4]. Looking at Figure S1, a bias could be detected, but its direction is not quite in line with the usual expectation (of large effect sizes at low precision): we have large effects in middle-sized studies, e.g., Milano-Malpensa, Athens, Berlin-Tegel, and Ho Chi Minh City.

S7. Exploring the heterogeneity of correlations between annoyance raw scores and noise levels

There are indications of **heterogeneity** in our sample of studies when we compare correlations between studies: The Q-test is statistically highly significant (Q = 397.877; df = 14; $p < 0.001$), and I^2, the ratio of true to total variance (Higgins & Thompson [5]) is 96.481, which means that a large part of the total variance is due to "true" variance between studies with respect to annoyance/level correlations.

Aggregating data from heterogeneous studies may be seen as a questionable enterprise. However, "heterogeneity is to be expected in a meta-analysis: it would be surprising if multiple studies, performed by different teams in different places with different methods, all ended up estimating the same underlying parameter." And "any amount of heterogeneity is acceptable, providing both that the predefined eligibility criteria for the meta-analysis are sound and that the data are correct." (Higgins, [6], p. 1158). There are two common ways to explore the causes of heterogeneity: (a) explore the influence of "outliers", and (b) explore study characteristics as potential effect moderators.

Ad (a) Even if we ignore the problem of defining an outlier, this method raises "important questions about the validity of the subsequent meta-analysis, since removal of studies is tantamount to manipulation of the [study] eligibility criteria" (Higgins, [6], p.1158). In addition, a study which seems to produce an outlier with respect to a certain effect size will not necessarily produce an outlier with respect to another effect size under consideration. In our case, this would further contribute to

a reduction in comparability between datasets which are not comparable between different effect size measures.

Ad (b) The second well-known method for exploring the causes for between-study heterogeneity is to explore study characteristics which may systematically differ between studies. With large datasets, this is usually done by means of meta-regression, using the effect size estimates as the dependent variable in the regression model and the study characteristics as covariates or predictor variables. (As the dependent variable is an effect size – e.g., the correlation between annoyance and the exposure level or an Odds Ratio –, the study characteristics can be interpreted as factors moderating the effect, i.e., the dose-response relationship.) However, Borenstein et al. [3] write on p.188: "As is true in primary studies, where we need an appropriately large ratio of subjects to covariates in order for the analysis to be meaningful, in meta-analysis we need an appropriately large ratio of studies to covariates. Therefore, the use of meta-regression, especially with multiple covariates, is not a recommended option when the number of studies is small. In primary studies some have recommended a ratio of at least ten subjects for each covariate, which would correspond to ten studies for each covariate in meta-regression." This requirement rules out performing meta-regression with datasets containing less than ten studies, but even with a considerable amount of studies, the result of a meta-regression analysis will depend on the distribution of the moderating variable within the dataset. With small datasets, a somewhat safer method is to use each of the potential moderators separately as a means to split up groups, and perform separate meta-analyses for each of the two groups, together with a mixed effects analysis comparing the groups. Mixed effect models assume a common study variance component among the studies within each subgroup and no common among-study variance component between the subgroups. This method has disadvantages, too, especially the risk of overestimating differences between groups – see next section.

Exploring the between-study heterogeneity of correlational effects

We explored the heterogeneity of aircraft noise annoyance studies with respect to correlations by means of subgroup analyses. Overall study quality, survey type, response rate, noise level range, and rate of airport change were used as potential moderators of the correlations between aircraft noise levels and individual annoyance judgments. It should be noted that all subgroup analyses reported here (and in the following sections on road and railway noise, too) are multiple post-hoc tests without Bonferroni correction for Q-values, and may be subject to confounding in the sense that studies which differ with respect to one dimension (e.g., survey type) may partially differ with respect to other dimensions as well (e.g., noise level range, or survey type). In order to counteract confounding it would have been desirable to perform meta-regressions involving several of the potential moderating factors as predictors in the same analysis. But this would require a greater number of studies; as a rule of thumb the ratio of the number of studies to the number of potentially moderating factors should be 10:1 or greater (see the preceding paragraph). The subgroup analyses reported here are explorative, and still have their value: they point to potential effect moderators.

With respect to **study quality,** it seems plausible that the effect size is related to study quality in the sense that increasing study quality generally contributes to decreasing error variance. On the other hand, study quality also relates to systematic effects, like sampling bias, which may result in biased exposure-response correlations. Therefore, we had no specific expectations with respect to the relation between study quality and the size of correlations. We divided the total group of 15 aircraft noise studies in two subgroups according to our study quality rating (see Table 1 in section 3.1.1 of the main text, rightmost column) into “higher” (quality rating >21) and “lower” (quality rating ≤21), and performed subgroup meta-analyses with correlations as effect size.

Results for “higher **quality**” (Berlin-Tegel, London-Heathrow, Athens, Milano-Malpensa, Amsterdam-Schiphol 2003-05, Stockholm-Arlanda, Zurich before 2001, Fraport, Amsterdam-Schiphol 2002) vs. “lower quality” (Cologne/Bonn, Hanoi, Ho Chi Minh City, Da Nang, Japan Airplanes, Trondheim):

- “higher quality” (nine studies): summary r = 0.479 (0.389 – 0.560); I^2 = 97.515
- “lower quality” (six studies): summary r = 0.365 (0.254 – 0.466); I^2 = 93.085

- Q between groups (mixed effect) = 2.747; df = 1; p = 0.097.

Although the point estimates of the pooled correlations are somewhat higher in the higher-quality group as compared to the lower-quality group, the Q-value of between-groups heterogeneity is not statistically significant. In other words: the study quality does not seem to have a systematic influence on the effect estimate based on correlations, and the heterogeneity within subgroups is still very high.

With respect to **survey type**, there was no clear expectation. Standardized face-to-face interviews, postal questionnaires, and telephone surveys all have their advantages and disadvantages. For instance, face-to-face interviews seem to be better suited to explore very personal experiences of respondents as compared to telephone and postal surveys; on the other hand, in face-to-face interviews the personal influence of interviewers is difficult to control. Higher annoyance scores have been reported with postal vs. non-postal surveys (Janssen et al., [7]). However, it is unclear whether there might be a similar effect with respect to exposure-annoyance correlations. We divided the total group of 15 aircraft noise studies in two subgroups according to the survey type "face-to-face" and "no-face-to-face" (telephone or postal survey) and performed a mixed effects meta-analysis. Joining postal and telephone surveys into one group may look questionable, but due to lack of studies, it was not possible to perform analyses separating the two.

Results for "**face-to-face**" (Berlin-Tegel, London-Heathrow, Athens, Milano-Malpensa, Amsterdam-Schiphol 2003-05, Stockholm-Arlanda, Frankfurt, Hanoi, Ho Chi Minh City, Da Nang) vs. "**no face-to-face**" (Zurich before 2001, Cologne/Bonn, Japan Airplanes, Trondheim, Amsterdam-Schiphol 2002):

- "face-to-face" (ten studies): summary r = 0.481 (0.388 – 0.564); I^2 = 96.737
- "no face-to-face" (five studies): summary r = 0.346 (0.302 – 0.388); I^2 = 70.732
- Q between groups (mixed effect) = 6.604; df = 1; p = 0.010.

The results of the mixed effects analysis between survey type groups show statistically significant higher point estimates of the correlations for the "face-to-face" group as compared to the "no-face-to-face" group. The heterogeneity (I^2) within the "face-to-face" group is extremely high as compared to the "no face-to-face" group. The Q-value of between-heterogeneity is statistically highly significant. In other words: face-to-face interviews show higher correlations between noise levels and annoyance scores as compared to no-face-to face interviews, but the heterogeneity within both groups is very high.

With respect to **response rate**, it is sometimes said that a low response rate in noise surveys may be associated with a selection bias in favor of people highly annoyed. This might be associated with different effects, e.g., higher annoyance judgments at all noise levels included in the study, higher annoyance judgments at certain noise levels, a restricted range of annoyance judgments, larger or smaller %HA differences at 10 dB increase of noise levels, and with a lower correlation between noise levels and annoyance judgments. In this section, we test the latter assumption. We divided the total group of 15 aircraft noise studies in two subgroups according to the response rate in the total survey: "high response rate" (>50%) and "low response rate" (<50%) and performed a mixed effects meta-analysis.

Results for "high **response rate**" (Athens, Stockholm-Arlanda, Zurich before 2001, Frankfurt, Hanoi, Ho Chi Minh City, Da Nang, Japan Airplanes) vs. "low response rate" (Berlin-Tegel, London-Heathrow, Milano-Malpensa, Amsterdam-Schiphol 2003-05, Cologne/Bonn, Trondheim, Amsterdam-Schiphol 2002):

- "high response rate" (eight studies): summary r = 0.398 (0.318 – 0.473); I^2 = 93.810
- "low response rate" (seven studies): summary r = 0.478 (0.354 – 0.586); I^2 = 97.889
- Q between groups (mixed effect) = 1.199; df = 1; p = 0.273.

The results of the mixed effects analysis between response rate groups do not show a statistically significant difference. The heterogeneity within each group is extremely high, and the subgroup classification according to response rate does not show any systematic relation with the correlations between noise levels and annoyance scores.

The effects of a restricted noise **level range** may be seen as an example of a well-known statistical effect: if two variables are submitted to a correlational analysis, the resulting correlation is generally

lower in case one of the variables shows little variation. As shown in Table 1 in section 3.1.1 of the main text, some aircraft noise surveys were done in a very narrow range of noise levels, e.g., 28-40 dB or 52-64 dB (i.e., a range of 12 dB), while others report a much wider range, e.g., 40-75 or even 12-80 dB. We divided the total group of 15 aircraft noise studies in two subgroups according to noise level range, a "high range" (>30 dB) and a "low range" group (<30 dB) and performed a mixed effects meta-analysis.

Results for "high **level range**" (Berlin-Tegel, London-Heathrow, Milano-Malpensa, Amsterdam-Schiphol 2003-05, Stockholm-Arlanda, Zurich before 2001, Amsterdam-Schiphol 2002) vs. "low level range" (Athens, Ho Chi Minh, Frankfurt, Hanoi, Cologne/Bonn, Japan Airplanes, Trondheim):

- "high level range" (seven studies): summary r = 0.486 (0.366 – 0.591); I^2 = 98.107
- "low level range" (eight studies): summary r = 0.390 (0.316 – 0.459); I^2 = 91.300
- Q between groups (mixed effect) = 1.900; df = 1; p = 0.168.

The results of the mixed effects analysis between level range groups does not show a statistically significant difference. The summary correlations are very similar, and the heterogeneity indices are very high. It should be noted that some of the studies with very low minimum noise levels (e.g., <35 dB) used a cut-off at 35 or 40 dB in their own statistical analyses; even if we follow this procedure when building subgroups, there is no statistically significant effect of the grouping according to level range classes.

With respect to rate of **airport change**, we expected correlations between annoyance scores and noise levels to be somewhat lower in airport change situations, because annoyance in change situations might be somewhat more influenced by the change situation as such, i.e., by the fact that the airport has changed or will change in the near future. In order to explore the influence of change, we divided the set of 13 aircraft noise studies according to the definition of change proposed by Janssen and Guski [8] as we did before in section 3.1.2. As a consequence, two groups of studies could clearly be defined, one group of eight studies, called "low rate change", and another group of five studies, called "high rate change" group. As before, both the Zurich 2001 and the Milano-Malpensa (2003-2005) did not fit exactly in one of the two groups. We performed a mixed-effect meta-analysis of correlations between level and annoyance in the two "change"-groups.

Results for "low rate **change**" (Berlin-Tegel, London-Heathrow, Cologne/Bonn, Hanoi, Ho Chi Minh City, Da Nang, Japan Airplanes, Trondheim) vs. "high rate change" (Athens, Amsterdam-Schiphol 2003-05, Stockholm-Arlanda, Frankfurt 2005, Schiphol 2002):

- "low rate change" (eight studies): summary r = 0.410 (0.311 – 0.499); I^2 = 94.331
- "high rate change" (five studies): summary r = 0.420 (0.351 – 0.485); I^2 = 92.910
- Q between groups (mixed effect) = 0.033; df = 1; p = 0.855

The two groups look similar with respect to their summary correlations and within-heterogeneity. There is no statistically significant difference between the two airport change groups. This may look as a contrast to the results shown in section 3.1.2 with respect to the higher percentage of highly annoyed persons at "high rate change" airports. But there is no contradiction, because (a) the meaning of the data (%HA on one side, and correlation coefficients on the other side) is very different, and (b) correlations are independent of the level of response, i.e., the same correlation may occur in two groups differing in mean annoyance and/or mean noise level.

In summarizing the results of five different approaches to explore the heterogeneity between studies, we have to state that there is only one moderator which shows statistically significant results (given the restrictions mentioned at the beginning of this section): "face-to-face" surveys on aircraft noise annoyance show higher correlations between aircraft noise levels and aircraft noise annoyance as compared to "no face-to-face" surveys – at least in our sample of 15 aircraft noise annoyance studies. The other potential moderators tested (overall study quality, response rate, noise level range, and rate of airport change) do not show statistically significant relations to the observed exposure-response correlations.

S8. Grading the quality of evidence for the correlation between aircraft noise levels and annoyance

The confidence in the evidence with respect to correlations between aircraft noise levels and aircraft noise annoyance may be decreased for several reasons, including

Study limitations: We have taken the study limitations into account by grading the quality of each study selected and using it in heterogeneity analyses, as far as possible.

Inconsistency of results: The meta-analysis of the full range of studies reveals wide confidence intervals and a high degree of heterogeneity, which could only to a small degree be attributed to the survey type (face-to-face interviews vs. no face-to-face). The tests related to overall study quality, response rate, noise level range, and rate of airport change did not show any statistically significant difference between respective groups. The heterogeneity between studies lead to a downgrading of the quality of evidence. Despite the heterogeneity, all studies show positive correlations between noise level and annoyance, and many studies show exposure-response relations.

Indirectness of evidence: **P**opulation, **E**xposure, **C**omparator, and **O**utcomes (PECO) in the studies selected here are judged to be comparable.

Imprecision: The samples of the studies reported here are between about 300 and nearly 6,000 in size, i.e., the precision is assumed to be high. In addition, the meta-analysis program weights the input data with respect to standard error and sample size.

Publication bias: The funnel plot of Figure S1 in section 5 of this Suppmentary shows a distribution of studies which is not in line with the expectation associated with publication bias: the largest effect sizes are seen in studies of medium precision – not in studies with low precision.

In sum, we are confident in the evidence with respect to correlations between aircraft noise levels and aircraft noise annoyance, and like to assign the grade "high quality" (see Table S3).

Table S3. GRADE summary of findings for the quality of evidence related to aircraft noise and degree of annoyance. Health outcome based on correlations, 15 studies.

Domains	Criterion	Assessment	Grading
Start Level	Study design: cross-sectional = high qual.	High quality	High quality
1. Study Limitations	Quality of majority of studies (risk of bias)	High quality of majority of studies	No downgrade
2. Inconsistency	Conflicting results; high I^2	High between study variance	Downgrade one level
3. Directness	Direct comparison; same PECO	Same PECO	No downgrade
4. Precision	Small sample sizes OR Low numbers of events (HA) OR Wide confidence intervals	Large study samples	No downgrade
5. Publication Bias	Funnel plot indicates	No publication bias	No downgrade
Overall Judgment			Moderate quality
6. Exposure-response	Statistically significant trend	All studies show statistically significant exposure-response relations	Upgrade one level
7. Magnitude of effect	Weighted mean r > .5	Weighted mean r = .436	No upgrade
8. Confounding adjusted	Effect in spite of confounding working towards the nil	No adjustments	No upgrade
Overall Judgment			High quality

PECO = Population, Exposure, Comparator, Outcome. For explanations see section S4.

S9. Figure S2 (funnel plot OR and %HA-difference for aircraft noise studies)

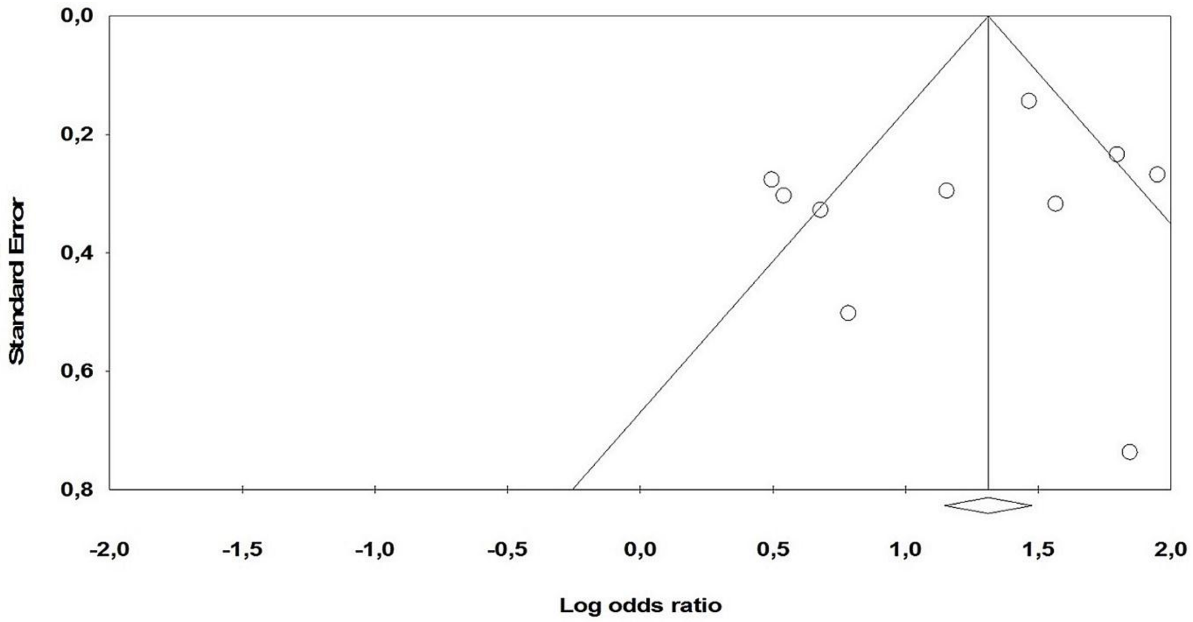

Figure S2. Funnel plot of the relation between the logarithmized Odds Ratios and standard errors of the %HA-difference effect in the sample of ten aircraft noise studies.

The funnel plot of the relation between logarithmized ORs and standard errors (Figure S2) shows a certain asymmetry with respect to higher standard errors (lower precision): there is one study showing a relatively small effect at relatively low precision (Arlanda), as well as a relatively large effect at an even lower precision (Zurich 2001). It is unclear whether this asymmetry may be due to publication bias.

S10. Exploring the heterogeneity of between-study heterogeneity of Odds Ratios in original grouped data

We explored the heterogeneity of aircraft noise annoyance studies with respect to the OR (referring to the increase of %HA by a 50-60 dB level increase) by means of subgroup analyses. As discussed in section 3.1.3 with respect to correlations as effect-size measures, we imagine five study characteristics to be possible effect moderators: study quality, survey type, response rate, noise level range, and rate of airport change. Subgroup comparisons for study quality and survey type could not be performed in this section, because there were less than three studies in one of the respective comparison groups. With decreasing numbers of studies in a subgroup, the results of observational studies are increasingly subject to uncontrollable influences.

Results for "wider **noise level range**" studies (Berlin-Tegel, London-Heathrow, Milano-Malpensa, Amsterdam-Schiphol, Stockholm-Arlanda, Zurich before 2001) vs. "smaller range studies" (Athens, Frankfurt, Hanoi, Da Nang):

- "wider range" (six studies): summary OR = 2.944 (1.813 – 4.782); I^2 = 74.414
- "smaller range" (four studies): summary OR = 4.243 (2.541 – 7.086), I^2 = 67.727
- Q between groups (mixed effect) = 1.029; df = 1; p = 0.310.

The two groups do not differ statistically significantly, although the summary OR is somewhat higher in the "smaller range" group as compared to the "wider range" group.

Results for "higher **response rate**" (Athens, Stockholm-Arlanda, Zurich before 2001, Frankfurt, Hanoi, Da Nang) vs. "lower response rate" (Berlin-Tegel, London-Heathrow, Milano-Malpensa, Amsterdam-Schiphol 2003-05):

- "higher response rate" (six studies): summary OR = 4.281 (2.917 – 6.281); I^2 = 61.597
- "smaller response rate" (four studies): summary OR = 2.532 (1.541 – 4.159); I^2 = 65.476
- Q between groups (mixed effect) = 2.693; df = 1; p = 0.101.

There is a statistically non-significant tendency for a somewhat greater OR for the increase of %HA between 50 and 60 dB L_{den} in studies with higher response rate, and the within heterogeneity is somewhat smaller in this group, but these differences may have occurred by chance.

Results for "high rate **airport change**" studies (Athens, Amsterdam-Schiphol 2003-05, Stockholm-Arlanda, Frankfurt 2005) vs. "low-rate change" studies (Berlin-Tegel, London-Heathrow, Hanoi, Da Nang):

- "high rate change" (four studies): summary OR = 3.377 (2.204 – 5.175); I^2 = 54.345
- "low rate change" (four studies): summary OR = 3.129 (1.341 – 7.302), I^2 = 84.415
- Q between groups (mixed effect) = 0.025; df = 1; p = 0.875.

There is no statistically significant effect of the rate of airport change on the OR for the increase of %HA from 50 to 60 dB L_{den}. On the other hand, it seems remarkable that the heterogeneity within the "high-rate change" group is considerably lower than within the "low-rate change" group.

In sum, attempts to explain the large degree of heterogeneity in the full set of aircraft noise studies reporting the %HA-difference between 50 and 60 dB L_{den} by means of subgroup analyses were not successful. Neither the range of noise levels, nor the response rate or the rate of airport change show a statistically significant influence on the OR.

S11. Meta-analysis based on modelled data

The parameters of a logistic regression of the exposure-response relationship (i.e., the slope parameter B and respective standard error -- calculated from logistic regressions using "highly annoyed" vs. "not highly annoyed" as dependent variable, and the noise exposure level as independent variable) were available only for four aircraft noise annoyance studies. Therefore, these four studies were selected for the meta-analysis of ORs based on modelled data. We used the slope parameter to estimate the OR for a 10 dB difference of exposure (either L_{den} or L_{dn}). This estimation describes the OR without referencing to a certain noise level – it refers to the full range of exposure levels used in a certain study. The results are illustrated in Figure S3.

Study name	Subgroup within study	Statistics for each study				
		Odds ratio	Lower limit	Upper limit	Z-Value	p-Value
Brink 2008	Zürich before 2001	2,737	2,201	3,405	9,044	0,000
Nguyen 2011	Hanoi	5,078	3,260	7,912	7,184	0,000
Nguyen 2011	Ho Chi Minh City	12,205	7,770	19,172	10,859	0,000
Nguyen 2012	DaNang	2,915	1,246	6,819	2,467	0,014
		4,778	2,272	10,048	4,124	0,000

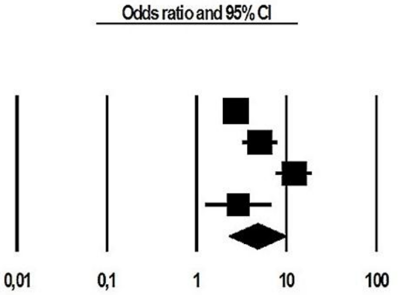

Figure S3. Odds Ratios for increase of %HA-by 10 dB L_{den} increase based on modelled exposure-response relations from four studies on aircraft noise annoyance. The right part of the graph contains a forest plot of the ORs and their respective 95 % confidence intervals. The figures of the last row indicate the summary estimates.

The meta-analysis on modelled data shows a somewhat higher summary OR (4.778) as compared to the analysis on observed data (summary OR = 3.405). The summary OR is greater than 1 and statistically highly significant (p < 0.001). However, the summary OR shows a wide confidence interval (from 2.272 to 10.048 in the summary row). This is wider than the summary confidence interval of the observed data.

The test on heterogeneity shows Q = 36.345; df = 3; p = 0.000; I2 = 91.746 which means that a very large percentage of the total variance is due to "true" variance between studies. Exploring the between-study heterogeneity of ORs based on modelled data is impossible to do in a systematic way,

since there are only four studies in the data set, and a subgroup analysis cannot produce reliable information.

S12. Grading the evidence based on Odds Ratios representing the %HA increase by a 10 dB Lden-increase of aircraft noise

The evidence with respect to OR used to determine the relative change of %HA with a 10 dB increase of aircraft noise in terms of L_{den} has been studied by means of two different types of data: (a) the difference between observed %HA at 50 vs. 60 dB (grouped observed data), and (b) the slope parameter of logistic regression analyses modelling the relation between %HA and noise exposure level. Both approaches led to statistically significant effects of the 10 dB aircraft noise level increase. The confidence in the evidence is somewhat mixed: on the one hand, the studies are consistent with respect to the direction on the effect: all studies show an increase in both types of data. On the other hand, a large variation with respect to the magnitude of the increase was observed, and the causes of this between-study heterogeneity could not be detected by the data at hand. Therefore, our confidence in the results is high with respect to the direction of the increase of %HA, but limited with respect to the magnitude of the increase. This limitation is due to several reasons, including

- **Study limitations**: As stated in the main text (section 3.1.1), we used data from observational studies which have been done by means of diverse methods of participant selection, survey type, and noise exposure assessment. We tried to take the study limitations into account by grading the quality of each study selected and using it in heterogeneity analyses, as far as possible.
- **Inconsistency of results**: The meta-analysis of the full range of studies reveals wide confidence intervals and a high degree of heterogeneity, which could not be explained by means of subgroup analyses.
- **Indirectness of evidence**: We do not see relevant differences between the population and the sample of participants included in the studies, except with respect to the age range (see Babisch-Hyena).
- **Imprecision**: The samples of the studies reported here are between about 300 and nearly 6,000 in size. In addition, the meta-analysis program weights the input data with respect to standard error and sample size.
- **Publication bias**: There is a certain unexplained asymmetry in the funnel plot of the meta-analysis based on observed data which might be due to publication bias.

In sum, we are confident in the evidence with respect to the direction of ORs indicating an-increase of %HA per 10 dB noise level increase, and like to assign the grade "high quality" in this regard (see Table S4 with respect to original grouped data, and Table S5 with respect to modelled data). We are uncertain with respect to the magnitude of the increase and like to assign the grade "moderate quality" in this regard.

Table S4. GRADE summary of findings for the quality of evidence related to aircraft noise and percent of highly annoyed persons. Health outcome: OR referring to the %HA increase per 10 dB level increase (50-60 dB L_{den}), based on original grouped data, ten studies.

Domains	Criterion	Assessment	Grading
Start Level	Study design: cross-sectional = high qual.	High quality	High quality
1. Study Limitations	Quality of majority of studies (risk of bias)	High quality of majority of studies	No downgrade
2. Inconsistency	Conflicting results; high I^2	High between study variance	Downgrade one level
3. Directness	Direct comparison; same PECO	Same PECO	No downgrade
4. Precision	Small sample sizes OR Low numbers of events (HA) OR Wide confidence intervals	Large study samples	No downgrade
5. Publication Bias	Funnel plot indicates	Small publication bias	Downgrade one level
Overall Judgment			Medium quality
6. Exposure-response	Statistically significant trend	Most studies show statistically significant ORs	Upgrade one level
7. Magnitude of effect	Weighted mean OR > 2.5	Weighted mean OR = 3.405	Upgrade one level
8. Confounding adjusted	Effect in spite of confounding working towards the nil	No adjustments	No upgrade
Overall Judgment			High quality

PECO = Population, Exposure, Comparator, Outcome. For explanations, see section S4.
OR = 2.5 converted to Cohen's [9] d = 0.5 = medium effect.

Table S5. GRADE summary of findings for the quality of evidence related to aircraft noise and percent of highly annoyed persons. Health outcome: OR referring to the %HA increase per 10 dB level increase, based on modelled data, four studies.

Domains	Criterion	Assessment	Grading
Start Level	Study design: cross-sectional = high qual.	High quality	High quality
1. Study Limitations	Quality of majority of studies (risk of bias)	High quality of majority of studies	No downgrade
2. Inconsistency	Conflicting results; high I^2	High between study variance	Downgrade one level
3. Directness	Direct comparison; same PECO	Same PECO	No downgrade
4. Precision	Confidence interval contains 25% harm or benefit and no effect OR optimal information size reached	Large study samples	No downgrade
5. Publication Bias	Funnel plot indicates	Not applicable, just four studies	Downgrade one level
Overall Judgment			Medium quality
6. Exposure-response	Statistically significant trend	All studies show statistically significant ORs	Upgrade one level
7. Magnitude of effect	Weighted mean OR > 2.5	Weighted mean OR = 4.778	Upgrade one level
8. Confounding adjusted	Effect in spite of confounding working towards the nil	No adjustments	No upgrade
Overall Judgment			High quality

PECO = Population, Exposure, Comparator, Outcome. For explanations, see section S4.
OR = 2.5 converted to Cohen's [9] d = 0.5 = medium effect.

S13. The influence of co-determinants in aircraft noise studies

Individual (or personal) confounding or moderating within-study variables are not considered here, but it should be kept in mind that they are of great importance in explaining the variance of individual annoyance judgments – they often show correlations with individual annoyance judgments of the same strength as do noise levels.

Attempts to explore study characteristics as between-study factors influencing the aircraft noise effects have been done in several sections of this chapter, and it turned out that there is at least one situational variable which seems to influence the relation between aircraft noise levels and aircraft noise annoyance: surveys done in "airport change situations" often report higher noise annoyance than surveys done in "no change" situations. This factor of "change" has been taken into account in section 3.1.4 of the main text, using the distinction between "high-rate change airports" and "low-rate change airports". Our presentation of the difference with respect to the height of %HA at "low-rate change" and "high-rate change" airports is no proof of the "change effect". However, we maintain that it should be considered in comparing exposure-response functions by different surveys, and by drawing "general" conclusions about the effects of aircraft noise on residents in the vicinity of airports.

Other attempts to find study characteristics as potential effect moderators have not been very successful. Only in the case of correlations, there is an indication that "face-to-face" interviews are

associated with higher correlations as compared to other survey types (telephone and postal interviews).

S14. Grading the quality of evidence for the exposure-response relation of %HA by road traffic noise in the full WHO dataset

To a certain extent, the arguments posed in section S4 with respect to the exposure-response relation of %HA by aircraft noise can be posed with respect to high road traffic noise annoyance in the full WHO Road dataset: at least, **study limitations** have been taken into account as far as possible. However, the **inconsistency of results** is greater here as compared to aircraft noise annoyance, because the environmental context (valleys vs. flat terrain, air-conditioned homes vs. unconditioned homes, public discussions about infrastructure change vs. no public discussion) differs somewhat between studies. The question of **indirectness of evidence** can be answered in the same manner as in section S4, although the age restriction due to the HYENA studies is less important here, due to the large Hong Kong study, which is a true random sample of the population. Another aspect contributing to indirectness is the difference between studies with respect to the criterion of being highly annoyed. With respect to **imprecision**, it should be noted that: the Hong Kong sample includes 10,077 residents and is rated as a "high quality" study. With respect to **publication bias**, we interpret the small asymmetry of the funnel plot seen with respect to correlations (see 3.2.2.1) as an indication of a slight publication bias. Therefore, the effect of noise levels on percent highly annoyed by road traffic noise may be somewhat overestimated.

With respect to the exposure-response relations reported here, it should be noted that 20 of 21 road traffic noise studies reporting a correlation show a statistically significant correlation between noise levels and raw scores (see 3.2.2), and they all show a clear increase of %HA with increasing noise levels, too. However, the methods used in order to show the relation between L_{den} and %HA varies between studies (e.g., some used binary logistic regression, some used a polynomial regression model), and we aggregated the resulting estimated data. In addition, only a minority of studies reported statistical information about the effect size (e.g., Nagelkerke's R^2). Due to these restrictions, an assessment in terms of GRADE both of the exposure-response relation itself and the size of the effect of noise levels on %HA was not possible.

In sum, we are not very confident in the evidence with respect to the exposure-response relation between road traffic noise levels and % highly annoyed by road traffic noise and like to assign the grade "low quality" (see Table S6).

Table S6. GRADE summary of findings for the quality of evidence related to road traffic noise levels and percent of high annoyance. Health outcome based on exposure-response relations, 20 studies.

Domains	Criterion	Assessment	Grading
Start Level	Study design: cross-sectional = high quality	High quality	High quality
1. Study Limitations	Quality of majority of studies (risk of bias)	High quality of majority of studies	No downgrade
2. Inconsistency	Conflicting results	Large scatter between studies	Downgrade one level
3. Directness	Direct comparison; same PECO	HA criterion differ between studies	Downgrade one level
4. Precision	Small sample sizes OR Low numbers of events (HA) OR Wide confidence intervals	Large study samples	No downgrade
5. Publication Bias	Funnel plot indicates	Not assessable	
Overall Judgment			Low quality
6. Exposure-response	Statistically significant trend %HA vs. L_{den}	Not assessable	
7. Magnitude of effect	Fit of logistic regression	Not assessable	
8. Confounding adjusted	Effect in spite of confounding working towards the nil	No adjustments	No upgrade
Overall Judgment			Low quality

PECO = Population, Exposure, Comparator, Outcome. For explanations, see section S4.

S15. Exploring the heterogeneity between road traffic noise studies with respect to correlations

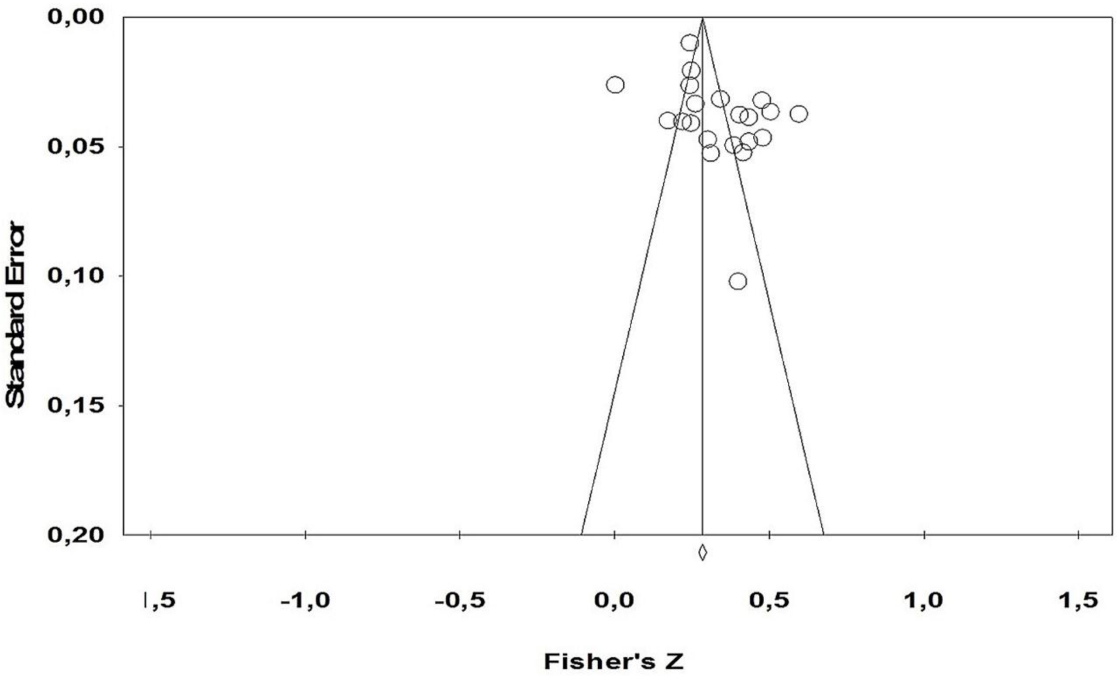

Figure S4. Funnel plot for the meta-analysis of 21 studies using Pearson correlations between L_{den} or L_{dn} and road traffic noise annoyance raw scores. "Fisher's Z" means Fisher's z', and is used as the effect indicator.

The funnel plot of the correlational analysis (Figure S4) shows a very slight asymmetry – at least somewhat more as compared to the respective plot for the correlational aircraft noise analysis. The points in Figure S4 seem to be slightly skewed from above left to down right. In other words, there may be some statistically non-significant low-effect studies missing, which may be due to a publication bias, and the effect of noise levels on road noise annoyance may be somewhat overestimated.

As expected, the test for heterogeneity is statistically highly significant: Q = 358.180; df = 20; p < 0.001. The I^2 = 94.416 which means that about 95 percent of the total variance is due to "true" variance between studies.

We explored the heterogeneity of road traffic noise annoyance studies with respect to correlations by means of subgroup analyses. Given the requirement of at least three studies in each of the comparison groups, overall study quality, survey type, response rate, noise exposure descriptor, noise level range, and response scale type could be used as potential moderators of the correlations between noise levels and individual annoyance judgments.

With respect to **study quality** (which relates mainly to the completeness of information given by the authors), we divided the total group of 21 road traffic noise studies providing correlations in two subgroups according to our study quality rating (see Table 3 in section 3.2 of the main text, rightmost column) into "higher" (quality rating 21) and "lower" (quality rating ≤21), and performed subgroup meta-analyses with correlations as effect size. Seven studies were rated as "higher quality" (Berlin-Tegel, London-Heathrow, Athens, Milano-Malpensa, Amsterdam-Schiphol, Stockholm-Arlanda, and Hong Kong), and 14 studies were rated as "lower quality" (Switzerland 2012-13, Da Nang, Hanoi, Ho Chi Minh City, Hue, Thai Nguyen, France 1997-98, Gothenburg Apartment, Gothenburg Detached, Kumamoto Apartment, Kumamoto Detached, Sapporo Apartment, Sapporo Detached, and France (Pierrette)).

Results with respect to **study quality**:

- "higher quality" (seven studies): summary r = 0.311 (0.233 – 0.386); I^2 = 93.999
- "lower quality" (14 studies): summary r = 0.333 (0.253 – 0.409); I^2 = 94.936
- Q between groups (mixed effect) = 0.322; df = 1; p = 0.695.

The two groups do not differ statistically significantly. We conclude that differences in study quality do not explain much of the between-study variance.

With respect to **survey type**, we divided the total group of 21 studies in two subgroups according to "face-to-face" (14 studies) and "no face-to-face" (seven studies). The "face-to-face" group consisted of Berlin-Tegel, London-Heathrow, Athens, Milano-Malpensa, Amsterdam-Schiphol, Stockholm-Arlanda, Da Nang, Hanoi, Ho Chi Minh City, Hue, Thai Nguyen, France 1997-98, Hong Kong, and France (Pierrette). The "no face-to-face" group consisted of Switzerland 2012-13, Gothenburg Apartment, Gothenburg Detached, Kumamoto Apartment, Kumamoto Detached, Sapporo Apartment, and Sapporo Detached.

Results with respect to **survey type**:

- "face-to-face" (14 studies): summary r = 0.317 (0.245 – 0.386); I^2 = 95.970
- "no face-to-face" (seven studies): summary r = 0.340 (0.281 – 0.397); I^2 = 78.600
- Q between groups (mixed effect) = 0.245; df = 1; p = 0.621.

The two groups do not differ statistically significantly. We conclude that differences in survey type do not explain much of the between-study variance.

With respect to **response rate**, we divided the group of 18 studies for which response rates were available, in two subgroups according to "high response rate" (>50%) and "low response rate" (<50%), and performed a mixed effects meta-analysis. Thirteen studies reported a "high response rate" (Athens, Stockholm-Arlanda, Da Nang. Ho Chi Minh City, Hue, Thai Nguyen, Hong Kong, Gothenburg Apartment, Gothenburg Detached, Kumamoto Apartment, Kumamoto Detached, Sapporo Appartment, and Sapporo Detached). Five studies reported a "lower response rate" (Berlin-Tegel, London-Heathrow, Milano-Malpensa, Amsterdam-Schiphol, and Hanoi).

Results with respect to **response rate**:

- "high response rate" (13 studies): summary r = 0.319 (0.243 – 0.391); I^2 = 95.350

- "low response rate" (five studies): summary r = 0.334 (0.228 – 0.432); I^2 = 93.580
- Q between groups (mixed effect) = 0.056; df = 1; p = 0.813.

The two groups do not differ statistically significantly. We conclude that differences in response rate do not explain much of the between-study variance.

With respect to **noise exposure descriptor**, we divided the total group of 21 studies in two subgroups according to noise exposure descriptor (L_{den} vs. L_{dn}). The L_{den}-group consisted of 14 studies (Berlin-Tegel, London-Heathrow, Athens, Milano-Malpensa, Amsterdam-Schiphol, Stockholm-Arlanda, Hong Kong, France 1997-98, France (Pierrette), Da Nang, Hanoi, Ho Chi Minh City, Hue, and Thai Nguyen). The L_{dn}-group consisted of seven studies (Switzerland 2012-13, Gothenburg Apartment, Gothenburg Detached, Kumamoto Apartment, Kumamoto Detached, Sapporo Apartment, and Sapporo Detached).

Results with respect to noise **exposure descriptor**:

- "L_{den}" (14 studies): summary r = 0.317 (0.245 – 0.386); I^2 = 95.970
- "L_{dn}" (seven studies): summary r = 0.340 (0.281 – 0.397); I^2 = 78.600
- Q between groups (mixed effect) = 0.245; df = 1; p = 0.621.

The two groups do not differ statistically significantly. We conclude that the noise level descriptor does not explain much of the variance between studies.

With respect to **noise level range**, we divided the total group of 21 studies in two subgroups according to noise level range, a "high range" (>30 dB) and a "low range" group (<30dB) and performed a mixed effects meta-analysis. The "high range" group consisted of ten studies (Berlin-Tegel, London-Heathrow, Athens, Milano-Malpensa, Amsterdam-Schiphol, Stockholm-Arlanda, Switzerland, Hong Kong, France 1997-98, and Gothenburg Apartment). The "low range" group consisted of 11 studies (Da Nang, Hanoi, Ho Chi Minh City, Hue, Thai Nguyen, France (Lyon), Gothenburg Detached, Kumamoto Apartment, Kumamoto Detached, Sapporo Apartment, and Sapporo Detached).

Results with respect to **noise level range**:

- "high range" (ten studies): summary r = 0.321 (0.263 – 0.377); I^2 = 93.018
- "low range" (11 studies): summary r = 0.330 (0.221 – 0.431); I^2 = 95.638
- Q between groups (mixed effect) = 0.021; df = 1; p = 0.885.

The two groups are very similar, there is no statistically significant difference. We conclude that differences in level range do not explain much of the between-study variance.

With respect to **response scale type**, we divided the total group of 21 studies in two subgroups according to "numerical scale" (seven studies, 11 scale steps) and "verbal scale" (14 studies, 4-5 scale steps). The "numerical scale" group consisted of France (Lyon), Gothenburg Apartment, Gothenburg Detached, Kumamoto Apartment, Kumamoto Detached, Sapporo Apartment, and Sapporo Detached. The "verbal scale" group consisted of Berlin-Tegel, London-Heathrow, Athens, Milano-Malpensa, Amsterdam-Schiphol 2003-05, Stockholm-Arlanda, Switzerland, Hong Kong, France 1997-98, Da Nang, Hanoi, Ho Chi Minh City, Hue, and Thai Nguyen.

Results with respect to **response scale type**:

- "numerical" (seven studies): summary r = 0.362 (0.328 – 0.396); I^2 = 9.990
- "verbal" (14 studies): summary r = 0.308 (0.242 – 0.372); I^2 = 95.969
- Q between groups (mixed effect) = 2.124; df = 1; p = 0.145.

The two groups do not differ statistically significantly. We conclude that differences in response scale type do not explain much of the between-study variance.

S16. Grading the evidence based on road traffic noise correlations

The arguments posed in section S14 with respect to exposure-response relations between %HA and road traffic noise levels can more or less be posed for the analysis of annoyance correlations: **study limitations** have been taken into account as far as possible, the **inconsistency of results** is somewhat greater here as compared to aircraft noise annoyance, because there is a zero correlation in one study, and the environmental context differs between studies in the full WHO road traffic

dataset. On the other hand, 20 of 21 studies show statistically highly significant positive correlations between road traffic noise level and annoyance scores. With respect to **publication bias**, we interpret the small asymmetry of the funnel plot as an indication of a slight publication bias. Therefore, the effect of noise levels on road noise annoyance may be somewhat overestimated.

In sum, we are moderately confident in the evidence with respect to correlations between road traffic noise levels and road traffic noise annoyance and like to assign the grade "moderate quality" (see Table S7).

Table S7. GRADE summary of findings for the quality of evidence related to road traffic noise and degree of annoyance. Health outcome based on correlations, 21 studies.

Domains	Criterion	Assessment	Grading
Start Level	Study design: cross-sectional = high quality	High quality	High quality
1. Study Limitations	Quality of majority of studies (risk of bias)	High quality of majority of studies	No downgrade
2. Inconsistency	Conflicting results; high I^2	High between study variance	Downgrade one level
3. Directness	Direct comparison; same PECO	Same PECO	No downgrade
4. Precision	Small sample sizes OR Low numbers of events (HA) OR Wide confidence intervals	Large study samples	No downgrade
5. Publication Bias	Funnel plot indicates	Slight publication bias	Downgrade one level
Overall Judgment			Low quality
6. Exposure-response	Statistically significant trend	20 of 21 studies show statistically significant exposure-response relations	Upgrade one level
7. Magnitude of effect	Weighted mean r > .5	Weighted mean r = .325	No upgrade
8. Confounding adjusted	Effect in spite of confounding working towards the nil	No adjustments	No upgrade
Overall Judgment			Moderate quality

PECO = Population, Exposure, Comparator, Outcome. For explanations, see section S4.

S17. Figure S5: Funnel plot of the relation between OR and %HA difference effect for road traffic noise, based on observed data

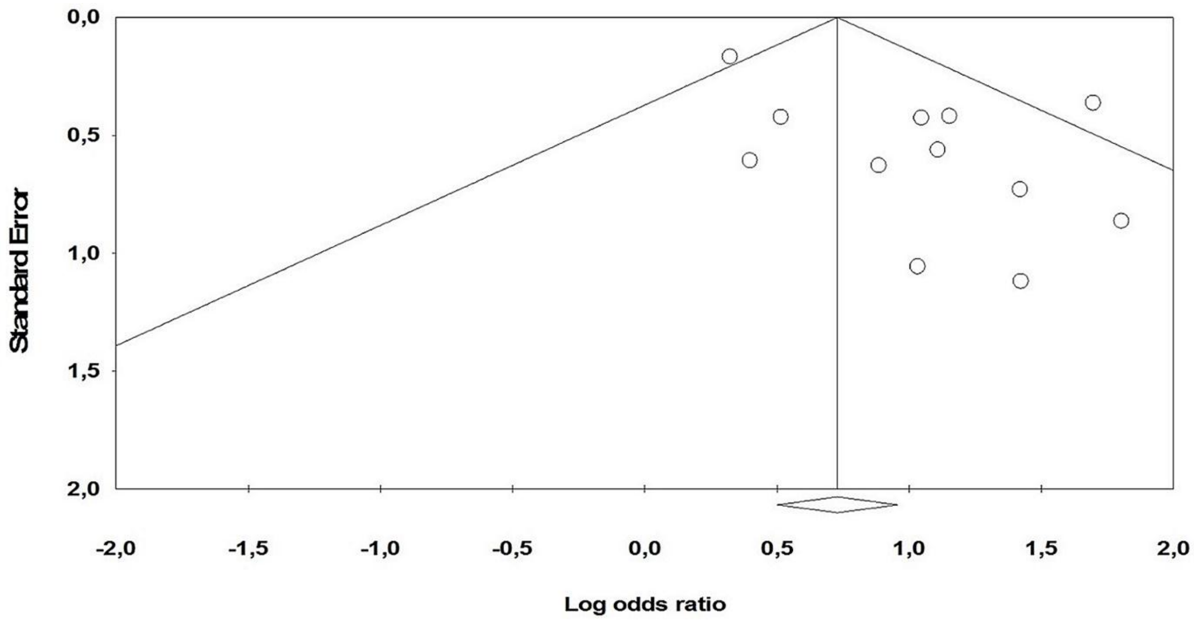

Figure S5. The funnel plot shows a definite asymmetry around the summary OR effect: there seem to be more low-precision studies reporting a high OR (log OR > 1.0) as there are high precision studies.

S18. Exploring the between-study heterogeneity of Odds Ratios in original grouped road traffic noise data

We explored the heterogeneity of road traffic noise annoyance studies with respect to the OR (referring to the increase of %HA by a 50-60 dB level increase) by means of subgroup analyses. Given the requirement of at least three studies in each of the comparison groups, overall study quality, survey type, noise exposure descriptor, response rate, and response scale type could be used as potential moderators of the ORs referring to the observed %HA increase per $L_{Aeq,24h}$ level increase from 50-60 dB.

With respect to study quality, we divided the group of 12 road traffic noise studies providing %HA data at comparable levels in two subgroups according to our study quality rating (see Table 3 in section 3.2 of the main text, rightmost column) into "higher" (quality rating >21) and "lower" (quality rating ≤21), and performed subgroup meta-analyses with correlations as effect size. Seven studies were rated as "higher quality" (Berlin-Tegel, London-Heathrow, Athens, Milano-Malpensa, Amsterdam-Schiphol, Stockholm-Arlanda, and Hong Kong), and five studies were rated as "lower quality" (Switzerland 2012-13, France 1997-98, Gothenburg Apartment, Gothenburg Detached, and Kumamoto Apartment).

Results with respect to study quality:

- "higher quality" (seven studies): summary OR = 2.893 (1.718 – 4.871); I^2 = 71.926
- "lower quality" (five studies): summary OR = 2.540 (1.415 – 4.561); I^2 = 0.000

Q between groups (mixed effect) = 0.106; df = 1; p = 0.745.

The two groups do not differ statistically significantly. We conclude that differences in study quality do not explain much of the between-study variance.

With respect to survey type, we divided the group of 12 studies providing %HA data at comparable levels in two subgroups according to "face-to-face" (eight studies) and "no face-to-face" (four studies). The "face-to-face" group consisted of Berlin-Tegel, London-Heathrow, Athens, Milano-Malpensa, Amsterdam-Schiphol, Stockholm-Arlanda, France 1997-98, and Hong Kong). The "no face-to-face" group consisted of Switzerland 2012-13, Gothenburg Apartment, Gothenburg Detached, and Kumamoto Apartment.

Results with respect to survey type:

- "face-to-face" (eight studies): summary OR = 2.876 (1.754 – 4.715); I2 = 67.348
- "no face-to-face" (four studies): summary OR = 2.518 (1.368 – 4.635); I^2 = 0.000

Q between groups (mixed effect) = 0.110; df = 1; p = 0.740.

The two groups do not differ statistically significantly. We conclude that differences in survey type do not explain much of the between-study variance.

With respect to noise exposure descriptor, we divided the group of 12 studies providing observed %HA differences at different noise exposure descriptors in two subgroups according to noise exposure descriptor (L_{den} vs. L_{dn}). The L_{den}-group consisted of eight studies (Berlin-Tegel, London-Heathrow, Athens, Milano-Malpensa, Amsterdam-Schiphol, Stockholm-Arlanda, Hong Kong, and France 1997-98). The L_{dn}-group consisted of four studies (Switzerland 2012-2013, Gothenburg Apartment, Gothenburg Detached, and Kumamoto Apartment).

Results with respect to noise exposure descriptor:

- "L_{den}" (eight studies): summary OR = 2.876 (1.754 – 4.715); I^2 = 67.348
- "L_{dn}" (four studies): summary OR = 2.518 (1.368 – 4.635); I^2 = 0.000

Q between groups (mixed effect) = 0.110; df = 1; p = 0.740.

The two groups do not differ statistically significantly. We conclude that the noise level descriptor does not explain much of the variance between studies.

With respect to response rate, we divided the group of ten studies for which both response rates and observed %HA data were available, in two subgroups according to "high response rate" (>50%) and "low response rate" (<50%), and performed a mixed effects meta-analysis. Six studies reported a "high response rate" (Athens, Stockholm-Arlanda, Hong Kong, Gothenburg Apartment, Gothenburg Detached, and Kumamoto Apartment). Four studies reported a "lower response rate" (Berlin-Tegel, London-Heathrow, Milano-Malpensa, and Amsterdam-Schiphol).

Results with respect to response rate:

- "high response rate" (six studies): summary OR = 2.430 (1.379 – 4.282); I^2 = 53.155
- "low response rate" (four studies): summary OR = 3.067 (1.846 – 5.095); I^2 = 44.881

Q between groups (mixed effect) = 0.360; df = 1; p = 0.549.

The two groups do not differ statistically significantly. We conclude that differences in response rate do not explain much of the between-study variance.

With respect to response scale type, we divided the total group of 12 studies which provided both original grouped data for %HA at 50 and 60 dB L_{den} and for response scale type in two subgroups according to "verbal scale" (three studies, 4-5 scale steps, cut-off mostly at 60% of the response scale) and "numerical scale" (nine studies, 11-steps response scale, cut-off at 73% of the response scale). The "verbal scale" group consisted of Gothenburg Apartment, Gothenburg Detached, and Kumamoto Apartment. The "numerical" group consisted of Berlin-Tegel, London-Heathrow, Athens, Milano-Malpensa, Amsterdam-Schiphol 2003-05, Stockholm-Arlanda, Switzerland, Hong Kong, and France 1997-98.

Results with respect to response scale type:

- "verbal" (three studies): summary OR = 2.254 (1.117 – 4.426); I^2 = 0.000
- "numerical" (nine studies): summary OR = 2.942 (1.844 – 4.693); I^2 = 64.013

Q between groups (mixed effect) = 0.405; df = 1; p = 0.525.

The two groups do not differ statistically significantly. We conclude that differences in response scale type do not explain much of the between-study variance.

S19. Meta-analysis based on modelled data

The parameters of a logistic regression of the exposure-response relationship (i.e., B, the respective standard error calculated from logistic regressions using "highly annoyed" vs. "not highly annoyed" as dependent variable, and the noise exposure level as independent variable) were available for 19 road traffic noise annoyance studies. We used the slope parameter to estimate the OR for a 10 dB difference of exposure in terms of L_{den} (11 studies), L_{dn} (seven studies) or $L_{Aeq,24h}$ (one study). The results are presented in Figure S6.

Study name	Subgroup within study	Odds ratio	Lower limit	Upper limit	Z-Value	p-Value
Brink 2013	CH (Vers. 2)	2,413	1,946	2,993	8,026	0,000
Shimoyama 2014	Da Nang	67,775	16,690	275,221	5,897	0,000
Shimoyama 2014	Hanoi	3,661	2,450	5,472	6,331	0,000
Shimoyama 2014	Ho Chi Minh City	1,644	0,864	3,128	1,515	0,130
Shimoyama 2014	Hue	4,066	1,964	8,420	3,777	0,000
Shimoyama 2014	Thai Nguyen	40,658	14,952	110,561	7,259	0,000
Champelovier 2003	France	2,460	2,022	2,992	9,000	0,000
Brown 2015	Hongkong	2,203	1,959	2,478	13,167	0,000
Lercher 2008	ALPNAP: Mainroad	2,255	1,943	2,617	10,697	0,000
Lercher 2008	ALPNAP: Motorway	2,654	2,190	3,216	9,959	0,000
Lercher 2008	BBT door-to-door: Mainroad	1,992	1,806	2,197	13,780	0,000
Lercher 2008	BBT door-to-door: Motorway	2,199	1,921	2,517	11,420	0,000
Lercher 2008	BBT phone: Motorway+Mainroad	1,818	1,548	2,136	7,293	0,000
Sato 2002	Gothenburg Appartment	4,166	2,934	5,914	7,981	0,000
Sato 2002	Gothenburg Detached	6,363	3,908	10,359	7,441	0,000
Sato 2002	Kumamoto Appartment	3,152	1,924	5,163	4,558	0,000
Sato 2002	Kumamoto Detached	3,694	2,207	6,183	4,971	0,000
Sato 2002	Sapporo Appartment	7,117	3,212	15,771	4,835	0,000
Sato 2002	Sapporo Detached	5,214	2,765	9,830	5,103	0,000
		3,033	2,592	3,549	13,843	0,000

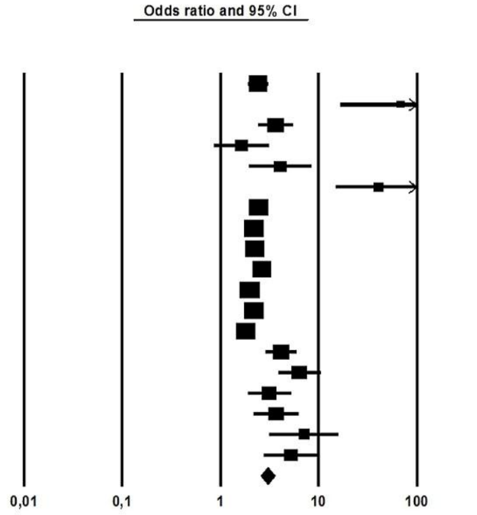

Figure S6. Odds Ratios and 95% confidence intervals for the increase of the rate of modelled "highly annoyed" data per 10 dB L_{den} increase of road traffic noise. The right part of the graph contains a forest plot of the ORs and their respective 95% confidence intervals. The figures of the last row indicate the summary estimates.

The summary effect of the 10 dB level increase from modelled data is somewhat greater (OR = 3.033; 95% CI = 2.592-3.549; $p < 0.001$) than we have seen in the foregoing analysis based on observed data. Except for the Ho Chi Minh study, all ORs are greater than 1 and highly statistically significant. On the other hand, the confidence intervals of the Da Nang and Thai Nguyen studies are very large, for reasons not known at present.

The heterogeneity test shows statistically highly significant differences between studies: Q = 129.605; df = 18; $p < 0.001$. The I^2 = 86.112 indicates that more than 80% of the total variance is due to "true" variance between studies.

S20. Figure S7. Funnel plot of the relation between OR and %HA difference effect for road traffic noise annoyance, based on modelled data

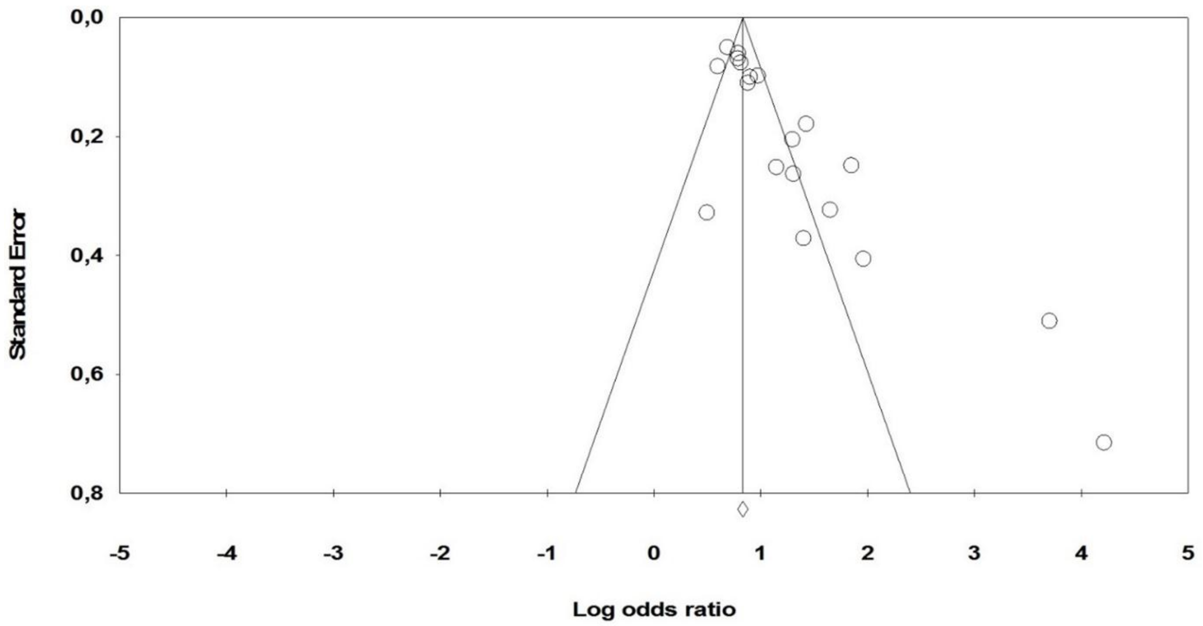

Figure S7. Funnel plot of the relation between the logarithmized Odds Ratios (based on modelled data) and standard errors of the %HA-difference effect in the sample of 19 road traffic noise studies.

The funnel plot of the relation between the logarithmized ORs (based on modelled data) and standard errors of the %HA-difference effect in the sample of 19 road traffic noise studies (Figure S7) is skewed: the two studies reporting the largest effects (Da Nang and Thai Nguyen) are associated with the two largest standard errors, and there is no study reporting lower effect sizes at lower standard errors. This situation indicates a bias towards over-estimation of effects estimated by modelled road traffic noise data.

We explored the effect of the two studies with extreme ORs (Da Nang and Thai Nguyen) by excluding them from an additional meta-analysis (not shown here) based on modelled data: The summary OR decreased from 3.033 to 2.683 (95% CI = 2.365 – 3.044; $p < 0.001$), but the between-study heterogeneity as well as the skewed funnel plot remain very similar. We conclude that even excluding the two studies with extreme ORs, there is a statistically highly significant effect of the 10 dB level increase (OR > 1 based on modelled data), but there still is a bias towards effect-overestimation.

S21. Exploring the between-study heterogeneity of Odds Ratios in modelled road traffic noise data

We explored the heterogeneity of road traffic noise annoyance studies with respect to the OR for a 10 dB level increase based on modelled data by means of subgroup analyses. Given the requirement of at least three studies in each of the comparison groups, overall study quality, noise level range, noise exposure descriptor, response rate, survey type, and response scale type could be used as potential moderators of the correlations between noise levels and individual annoyance judgments.

With respect to study quality, we divided the group of 19 road traffic noise studies providing modelled data in two subgroups according to our study quality rating (see Table 3 in section 3.2 of the main text, rightmost column) into "higher" (quality rating >21) and "lower" (quality rating ≤21), and performed subgroup meta-analyses with ORs effect size. Six studies were rated as "higher quality" (Hong Kong, and the five Alpine studies), and 13 studies were rated as "lower quality" (Switzerland 2012-13, Da Nang, Hanoi, Ho Chi Minh City, Hue, Thai Nguyen, France 1997-98, Gothenburg Apartment, Gothenburg Detached, Kumamoto Apartment, Kumamoto Detached, Sapporo Apartment, and Sapporo Detached).

Results with respect to study quality:

- "higher quality" (six studies): summary OR = 2.151 (1.975 – 2.342); I^2 = 57.064
- "lower quality" (13 studies): summary OR = 4.501 (3.274 – 6.186); I^2 = 84.565
- Q between groups (mixed effect) = 19.303; df = 1; p = 0.000.

The two groups differ statistically significantly. Lower quality studies are associated with larger ORs. We conclude that differences in study quality may explain parts of the between-study variance.

With respect to noise level range, we divided the total group of 14 studies which provided both the L_{den}-range as well as modelled data on %HA in two subgroups according to noise level range, a "high range" (>30 dB) and a "low range" group (<30dB) and performed a mixed effects meta-analysis. The "high range" group consisted of four studies (Switzerland, Hong Kong, France 1997-98, and Gothenburg Apartment). The "low range" group consisted of ten studies (Da Nang, Hanoi, Ho Chi Minh City, Hue, Thai Nguyen, Gothenburg Detached, Kumamoto Apartment, Kumamoto Detached, Sapporo Apartment, and Sapporo Detached).

Results with respect to noise level range:

- "high range" (four studies): summary OR = 2.584 (2.119 – 3.150): I^2 = 74.090
- "low range" (ten studies): summary OR = 5.700 (3.581 – 9.071); I^2 = 81.810
- Q between groups (mixed effect) = 9.424; df = 1; p = 0.002.

The two groups differ statistically significantly: "low level range" studies show higher ORs based on modelled data as compared to "high level range" studies. On the other hand, it should be noted that the "low level range" studies in our analysis all are related to the higher levels, starting at 46 dB, while the "high level range" studies start several decibels lower (e.g., at 30 dB in the Hong Kong study). We conclude that differences in level range do explain parts of the between-study variance, but there may be a confounding effect of the absolute position of the study within the range of noise levels.

With respect to noise exposure descriptor (L_{den} vs. L_{dn}), we divided the group of 19 studies providing modelled data in two subgroups according to "L_{den}" and "L_{dn}". The L_{den}-group consisted of 12 studies (Hong Kong, France 1997-98; five Alpine studies, Da Nang, Hanoi, Ho Chi Minh City, Hue, and Thai Nguyen). The L_{dn}-group consisted of seven studies (Switzerland 2012-13, Gothenburg Apartment, Gothenburg Detached, Kumamoto Apartment, Kumamoto Detached, Sapporo Apartment, and Sapporo Detached).

Results with respect to noise exposure descriptor:

- "L_{den}" (12 studies): summary OR = 2.580 (2.184 – 3.047); I^2 = 86.198
- "L_{dn}" (seven studies): summary OR = 4.063 (2.933 – 5.630); I^2 = 73.422
- Q between groups (mixed effect) = 5.917; df = 1; p = 0.015.

The two groups differ statistically significantly – ignoring the restrictions due to multiple testing etc. The L_{dn}-group shows somewhat higher ORs as compared to the L_{den}-group. It should be noted that a similar effect of the exposure descriptor was not observed in the analysis based on observed data.

With respect to survey type, we divided the group of 19 studies providing modelled data in two subgroups according to "face-to-face" (nine studies) and "no face-to-face" (ten studies). The "face-to-face" group consisted of Hong Kong, France 1997-98, two Alpine studies, Da Nang, Hanoi, Ho Chi Minh City, Hue, and Thai Nguyen. The "no face-to-face" group consisted of Switzerland 2012-13, three Alpine studies, Gothenburg Apartment, Gothenburg Detached, Kumamoto Apartment, Kumamoto Detached, Sapporo Apartment, and Sapporo Detached.

Results with respect to survey type:

- "face-to-face" (nine studies): summary OR = 2.941 (2.317 – 3.732); I^2 = 88.657
- "no face-to-face" (ten studies): summary OR = 3.168 (2.525 – 3.973); I^2 = 83.455
- Q between groups (mixed effect) = 0.197; df = 1; p = 0.667.

The two groups do not differ statistically significantly. We conclude that differences in survey type do not explain much of the between-study variance.

With respect to response rate, we divided the group of 17 studies for which both response rates and modelled %HA data were available, in two subgroups according to "high response rate" (>50%) and "low response rate" (<50%), and performed a mixed effects meta-analysis. Fourteen studies

reported a “high response rate” (Hong Kong, three Alpine studies, Gothenburg Apartment, Gothenburg Detached, Kumamoto Apartment, Kumamoto Detached, Sapporo Apartment, Sapporo Detached, Da Nang, Ho Chi Minh City, Hue, and Thai Nguyen). Three studies reported a “lower response rate” (two Alpine studies, and Hanoi).

Results with respect to response rate:

- “high response rate” (14 studies): summary OR = 3.485 (2.779 – 4.372); I^2 = 89.224
- “low response rate” (three studies): summary OR = 2.628 (2.118 – 3.262); I^2 = 64.665
- Q between groups (mixed effect) = 3.122; df = 1; p = 0.077.

The two groups do not differ statistically significantly, although there is a tendency for higher ORs at low response rates. On the other hand, the group of “low response rate” studies is very small. We conclude that differences in response rate do not explain much of the between-study variance.

With respect to response scale type, we divided the total group of 19 studies which provided both exposure-response functions for %HA and for response scale type in two subgroups according to “verbal scale” (nine studies, 4-5 response scale steps) and “numerical scale” (ten studies, 11 response scale steps). The “verbal scale” group consisted of three Alpine studies, Gothenburg Apartment, Gothenburg Detached, Kumamoto Apartment, Kumamoto Detached, Sapporo Apartment, and Sapporo Detached. The “numerical” group consisted of Switzerland, Hong Kong, France 1997-98, two Alpine studies, Da Nang. Hanoi, Ho Chi Minh City, Hue, and Thai Nguyen.

Results with respect to response scale type:

- “verbal” (nine studies): summary OR = 3.345 (2.570 – 4.354); I^2 = 85.281
- “numerical” (ten studies): summary OR = 2.819 (2.284 – 3.481); I^2 = 87.349
- Q between groups (mixed effect) = 0.985; df = 1; p = 0.321.

The two groups do not differ statistically significantly. We conclude that differences in response scale type do not explain much of the between-study variance.

S22. Grading the evidence of Odds Ratios representing the %HA- increase per 10 dB level increase of road traffic noise.

Similar arguments as posed in section S16 with respect to the road traffic noise annoyance evidence based on correlations can be posed with respect to the evidence of OR representing the %HA increase per 10 dB increase of road traffic noise level: **study limitations** have been taken into account as far as possible, the **inconsistency of results** is similar here as compared to the correlational analyses. However, the reasons differ: all level effects indicate a %HA increase (in terms of OR > 1), but the size of the effect differs between studies – there are even several studies reporting statistically non-significant effects, especially on observed data. On the other hand, 18 of 19 studies show ORs based on modelled data, which are greater than 1 and statistically highly significant. The question of **indirectness of evidence** can be answered in the same manner as in sections S14 and S16, while the question of **imprecision** must be discussed: with observed data on the %HA difference between 50 and 60 dB, we found a large variation in the number of participants within these two level classes, while this problem does not occur with modelled data. On the other hand, it is difficult to decide whether the difference between ORs based on observed data and ORs based on modelled data is due to the fact that the former explicitly uses a well specified level difference (50-60 dB) while the latter uses a mathematical model and a level difference which is not bound to any specific noise level, or the difference between ORs is simply due to the fact that one uses observed data and the other modelled ones. With respect to **publication bias**, we interpret the asymmetry of the funnel plots for the original grouped data as well as for the modeled data as an indication of a bias. The effect of the 10 dB difference in noise levels on %HA by road noise may be overestimated.

The quality of evidence is moderate in the case of original data (see Table S8), and high in the case of modelled data (see Table S9).

Table S8. GRADE summary of findings for the quality of evidence related to road traffic noise and percent of highly annoyed persons. Health outcome: OR referring to the %HA increase per 10 dB level increase (50-60 dB L_{den}), based on original grouped data, 12 studies.

Domains	Criterion	Assessment	Grading
Start Level	Study design: cross-sectional = high quality	High quality	High quality
1. Study Limitations	Quality of majority of studies (risk of bias)	High quality of majority of studies	No downgrade
2. Inconsistency	Conflicting results; high I^2	High between study variance	Downgrade one level
3. Directness	Direct comparison; same PECO	Same PECO	No downgrade
4. Precision	Small sample sizes OR Low numbers of events (HA) OR Wide confidence intervals	Large study samples	No downgrade
5. Publication Bias	Funnel plot indicates	Small publication bias	Downgrade one level
Overall Judgment			Low quality
6. Exposure-response	Statistically significant trend	Half of the studies show statistically significant ORs	No upgrade
7. Magnitude of effect	Weighted mean OR > 2.5	Weighted mean OR = 2.738	Upgrade one level
8. Confounding adjusted	Effect in spite of confounding working towards the nil	No adjustments	No upgrade
Overall Judgment			Moderate quality

PECO = Population, Exposure, Comparator, Outcome. For explanations, see section S4. OR = 2.5 converted to Cohen's [9] d = 0.5 = medium effect.

Table S9. GRADE summary of findings for the quality of evidence related to road traffic noise and percent of highly annoyed persons. Health outcome: OR referring to the %HA increase per 10 dB level increase, based on modelled data, 19 studies.

Domains	Criterion	Assessment	Grading
Start Level	Study design: cross-sectional = high quality	High quality	High quality
1. Study Limitations	Quality of majority of studies (risk of bias)	High quality in the majority of studies	No downgrade
2. Inconsistency	Conflicting results; high I^2	High between study variance	Downgrade one level
3. Directness	Direct comparison; same PECO	Same PECO	No downgrade
4. Precision	Confidence interval contains 25% harm or benefit and no effect OR optimal information size reached	Large study samples	No downgrade
5. Publication Bias	Funnel plot indicates	Small publication bias	Downgrade one level
Overall Judgment			Low quality
6. Exposure-response	Statistically significant trend	18 out of 19 studies show statistically significant ORs	Upgrade one level
7. Magnitude of effect	Weighted mean OR > 2.5	Weighted mean OR = 3.033	Upgrade one level
8. Confounding adjusted	Effect in spite of confounding working towards the nil	No adjustments	No upgrade
Overall Judgment			High quality

PECO = Population, Exposure, Comparator, Outcome. For explanations, see section S4. OR = 2.5 converted to Cohen's [9] d = 0.5 = medium effect.

S23. The influence of co-determinants in road traffic noise studies

As stated in section S13, individual noise annoyance judgments of residents are to a large extent influenced by confounding or moderating personal variables (e.g., noise sensitivity, and coping capacity). We do not discuss these within-study variables here. Instead, we like to mention between-study co-determinants which apply to many residents and should be taken into account when analyzing noise annoyance from road traffic noise:

a) Environmental conditions relating to the sound transmission between source and survey participants: Most of the noise surveys in Europe take place in rather flat terrains, and in homes without air-conditioning. If we compare studies performed in valleys with studies performed in flat terrain, we should take care of the so-called amphitheater effect, i.e., the propagation of sound to the valley slopes, including back-and-forth reflections of sounds produced in the valley. In the past, it has been shown that annoyance responses are usually higher in Alpine areas than in non-Alpine areas at similar levels of continuous sound levels. If we compare studies performed in air-conditioned homes with studies where air-conditioning is rare, we should be aware that the standard ICBEN/ISO annoyance question does not distinguish between inside and outside. However, study participants in air-conditioned homes may mostly relate to the inside of the house, while the responses in non-air-conditioned homes will relate both to the outside and the inside.

b) Access to quietness: Since Öhrström et al. [10] published their paper on the benefits of access to quietness, a series of papers (mostly from Scandinavia) showed data supporting the hypothesis that residential road traffic noise annoyance is partially reduced by means of a "quiet façade" (i.e., a less exposed side of the dwelling) and/or "access to quiet areas" (i.e., recreational areas in the vicinity of the dwelling). For instance, De Kluizenaar et al. [11] report that the availability of a relatively quiet façade at home is associated with less road traffic noise annoyance, compared to noise annoyance levels of Amsterdam residents with similar noise levels at the most exposed façade.

c) Motorway vs. urban roads: Based on a large European survey including more than 5,000 participants, Miedema [12, p. 33] concluded: "At higher levels highways cause more annoyance than other road traffic". In order to explain this difference, one hypothesis could be the difference in quiet moments: Highways usually do not have any quiet period at all, but other roads usually do – at least during the night and oftentimes also during the day. There are other differences between highways and urban main roads, e.g., the percentage of heavy (and loud) trucks is usually larger at highways than at urban main roads (at comparable L_{Aeq}) and higher during the night than during daytime. This day/night difference is much smaller at highways. On the other hand, Lercher et al. [13] asked whether noise from a main road could be more annoying than noise from a highway. By means of traffic modeling and survey information from two studies in alpine valleys, the authors found that under certain conditions of topography, traffic composition, and settlement patterns main roads may be associated with higher annoyance, compared to highways. Today, "fluctuation strength" and "intermittency ratio" [14] in the sound pattern are concepts which may help to increase the power of noise descriptors to predict health effects.

These factors also should be taken into account, if results between different studies are to be compared.

S24. Grading the quality of evidence for the exposure-response relation of %HA by railway traffic noise

To a certain extent, the arguments posed in section S14 with respect to road traffic noise annoyance exposure-response relations can be posed with respect to railway traffic noise annoyance ERRs: **study limitations** have been taken into account as far as possible, the **inconsistency of results** is shown by the large spread of data points at medium and high noise levels, partially due to different environmental conditions between studies (leading to a downgrade). This time, the **directness** of comparisons between studies is reduced, because about one half of the studies use a different definition of "highly annoyed" as compared to the other half, resulting in an additional downgrading. On the other hand, we do not see relevant differences between the population and the sample of participants included in the studies. **Imprecision** is no problem, since we deal with sample sizes between about 500 to 2,000 participants. We do not see any indication of a **publication bias.** All studies show statistically significant exposure-response relations (leading to an upgrade), and most of the studies provide an indication of a noise effect in terms of Pseudo-$R^2 > 0.10$.

In sum, we are moderately confident in the evidence with respect to exposure-response relations between railway noise levels and percentage of high railway traffic noise annoyance, and like to assign the grade "Moderate quality" (see Table S10).

Table S10. GRADE summary of findings for the quality of evidence related to railway noise levels and %HA. Health outcome based on exposure-response relations, nine studies.

**Domains**	**Criterion**	**Assessment**	**Grading**
Start Level	Study design: cross-sectional = high quality	high quality	High quality
1. Study Limitations	Quality of majority of studies (risk of bias)	High quality of majority of studies	No downgrade
2. Inconsistency	Conflicting results	High between study variance	Downgrade one level
3. Directness	Direct comparison; same PECO	The definition of HA differs between studies	Downgrade one level
4. Precision	Small sample sizes OR Low numbers of events (HA) OR Wide confidence intervals	Large study samples	No downgrade
5. Publication Bias	Funnel plot indicates	No indication of publication bias	No downgrade
Overall Judgment			Low quality
6. Exposure-response	Statistically significant trend	All studies show statistically significant exposure-response relations	Upgrade one level
7. Magnitude of effect	Fit of logistic regression	Most of the studies provided R2 > 0.10	No upgrade
8. Confounding adjusted	Effect in spite of confounding working towards the nil	No adjustments	No upgrade
**Overall Judgment**			**Moderate quality**

PECO = Population, Exposure, Comparator, Outcome. For explanations, see section S4.

S25. Figure S8. Funnel plot for the meta-analysis of eight studies using Pearson correlations between $L_{Aeq,24h}$ and railway noise annoyance raw scores

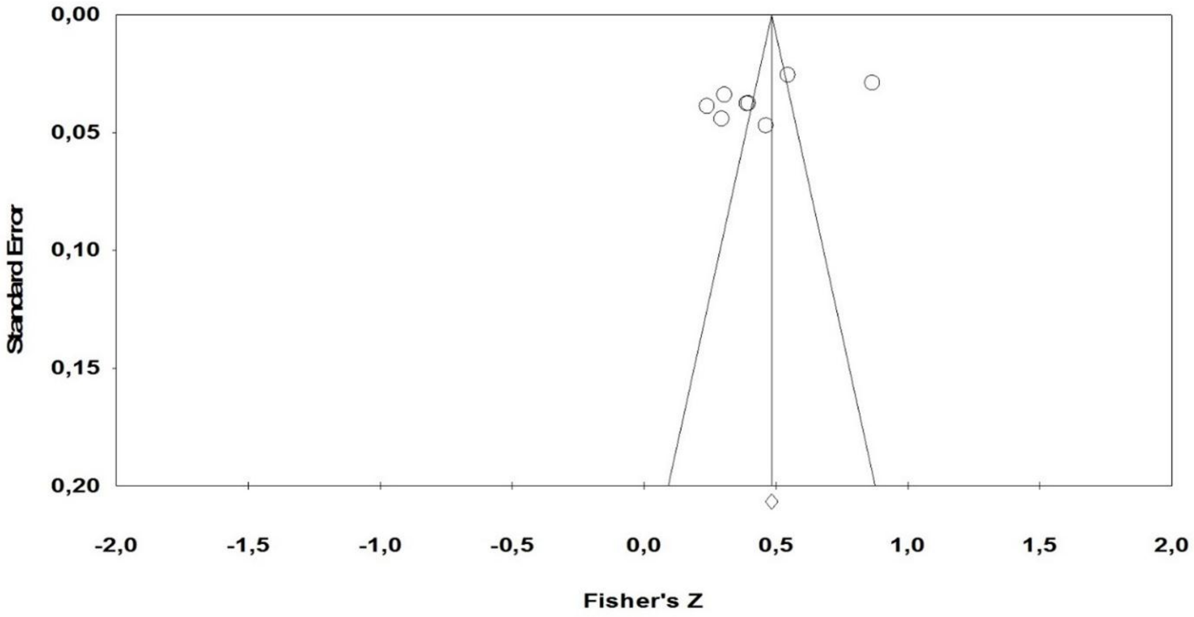

Figure S8. Funnel plot for the meta-analysis of eight studies using Pearson correlations between $L_{Aeq,24h}$ and railway noise annoyance raw scores. "Fisher's Z" = Fisher's z'. Note: two of the circles overlap almost completely.

The funnel plot (Figure S8) of the correlational analysis shows an asymmetric relation to the mean weighted noise effect: six of the eight studies are left of the mean of Fisher's z'. The utmost right point (Rhine valley study) seems to be an "outlier" – at least, there are other studies with similar effects missing. The unusual high correlation observed in this Rhine valley study can neither be easily attributed to any technical irregularity, nor to the long lasting public discussions about effects of railway noise in the study areas, except if we assume that public discussion of noise effects contributes to increased coefficients of correlation between exposure and annoyance, especially at higher noise levels. Another possible cause for the high correlation might be that railway sound calculations were done as close-grained as possible, that is, the loudest façade as well as the floor level of the resident's home was included in the sound level calculations. At present, we can state that the correlational effect of noise levels on railway noise annoyance raw scores seems to be somewhat biased to the right - not in the sense of overestimation associated with high standard errors; the distribution of effect sizes around the summary correlation simply is not symmetric.

The test on heterogeneity between the eight studies was statistically highly significant: $Q = 279.544$; $df = 7$; $p < 0.001$. The $I^2 = 97.496$ - which means that a very large part of the total variance is due to variance between studies.

S26. Exploring the heterogeneity between railway noise studies, based on correlations

Yano et al. [15] found that the vibration levels from Shinkansen trains in their study were statistically significant higher than those from conventional railways, and that railway noise annoyance assessed at this line seemed to be strongly associated with vibrations, as well as with the infrastructure changes in the survey areas. Both aspects may be a cause for the between-study variance, and we performed the same meta-analysis as above excluding the Yano-Shinkansen study (Figure S9).

The summary correlation of meta-analysis on correlations, excluding the Yano-Shinkansen study, ($r = 0.417$, Figure S9) is very similar to the one reported before (including the Yano-Shinkansen study), and the heterogeneity is very similar, too ($Q = 273.366$; $df = 6$; $p < 0.001$; $I^2 = 97.805$). In other words: The exclusion of the Yano-Shinkansen study did not reduce the variance between studies.

Study name	Subgroup within study	Statistics for each study				
		Correlation	Lower limit	Upper limit	Z-Value	p-Value
Gidloef-G. 2012	Survey1: rail, no vibrations	0,286	0,205	0,363	6,670	0,000
Gidloef-G. 2012	Survey2: rail, noise & vibrations	0,432	0,354	0,504	9,851	0,000
Gidloef-G. 2012	Survey3: rail, many trains	0,371	0,306	0,433	10,359	0,000
Yano 2005	conventional rail	0,497	0,458	0,534	21,367	0,000
Schreckenb. 2013	rail: Rheintal	0,699	0,669	0,727	30,051	0,000
Champelovier 2003	rail: France	0,234	0,161	0,305	6,164	0,000
Yokoshima 2008	Shinkansen: Kanagawa	0,296	0,234	0,355	8,995	0,000
		0,417	0,263	0,550	4,989	0,000

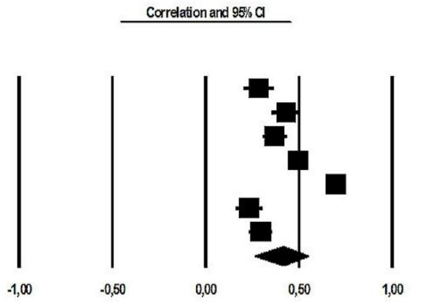

Figure S9. Meta-analysis of seven studies using Pearson correlations between $L_{Aeq,24h}$ and railway noise annoyance raw scores. The right part of the graph contains a forest plot of the correlations and their respective 95% confidence intervals. The figures of the last row indicate the summary estimates.

In order to look for other sources of between-study variance, we performed subgroup analyses with respect to correlations between railway noise levels ($L_{Aeq,24h}$) and individual annoyance judgments. Given the requirement of at least three studies in each of the comparison groups, only overall study quality and noise level range could be used as potential moderators of the correlations.

With respect to study quality, we divided the group of seven railway noise studies (after excluding the Yano-Shinkansen study) in two subgroups according to our study quality rating (see Table 5 in section 3.3 of the main text, rightmost column) into "higher" (quality rating >21) and "lower" (quality rating ≤21), and performed subgroup meta-analyses with correlations as effect size. Three studies were rated as "higher quality" (two Gidlöf studies and the Rhine valley study), four studies were rated as "lower quality" (France 1997-98, a Gidlöf study, a Japanese conventional trains study, and the Kanagawa Shinkansen study).

Results with respect to study quality:

- "higher quality" (three studies): summary r = 0.518 (0.243 – 0.716); I^2 = 98.331
- "lower quality" (four studies): summary r = 0.334 (0.190 – 0.465); I^2 = 95.247
- Q between groups (mixed effect) = 1.509; df = 1; p = 0.219.

Although the higher quality studies seem to be associated with higher correlations, the two groups do not differ statistically significantly. We conclude that differences in study quality do not explain much of the between-study variance.

With respect to noise level range, we divided the group of seven studies in two subgroups according to noise level range (a "high range" (>30 dB $L_{Aeq,24h}$) and a "low range" group (<30dB $L_{Aeq,24h}$)) and performed a mixed effects meta-analysis. The "high range" group consisted of four studies (France 1997-98, Rhine valley, Japan conventional trains, and Shinkansen Kanagawa). The "low range" group consisted of three Gidlöf studies (no vibration, noise + vibration, many trains).

Results with respect to noise level range:

- "high range" (four studies): summary r = 0.454 (0.216 – 0.641); I^2 = 98.729
- "low range" (three studies): summary r = 0.364 (0.283 – 0.439); I^2 = 71.129
- Q between groups (mixed effect) = 0.553; df = 1; p = 0.457.

The two groups are very similar; there is no statistically significant difference. We conclude that differences in level range do not explain much of the between-study variance.

S27. Grading the evidence based on railway noise correlations

To a large extent, the arguments posed in section S15 with respect to road traffic noise annoyance correlations can be posed with respect to railway traffic noise annoyance correlations. **Study limitations** have been taken into account as far as possible, and the **inconsistency of results** is similar to the road traffic noise correlations: the height of the railway correlations mainly varies from r = 0.234 to 0.497 – with one exception (r = 0.699 in the Rhine valley study). All correlations are statistically highly significant and positive. With respect to the **indirectness** of evidence, we do not see relevant differences between the population and the sample of participants included in the studies.

Imprecision is no problem, since we deal with sample sizes from about 500 to 2,000 participants. With respect to **publication bias**, the scatter around the mean summary correlation is not asymmetric in a sense that could be easily interpreted as an indication of a publication bias.

In sum, we are confident in the evidence with respect to correlations between railway noise levels and road traffic noise annoyance, and like to assign the grade "High quality" – see Table S11.

Table S11. GRADE summary of findings for the quality of evidence related to railway noise and degree of annoyance. Health outcome based on correlations, eight studies.

Domains	Criterion	Assessment	Grading
Start Level	Study design: cross-sectional = high quality	High quality	High quality
1. Study Limitations	Quality of majority of studies (risk of bias)	High quality of majority of studies	No downgrade
2. Inconsistency	Conflicting results; high I2	High between study variance	Downgrade one level
3. Directness	Direct comparison; same PECO	Same PECO	No downgrade
4. Precision	Small sample sizes OR Low numbers of events (HA) OR Wide confidence intervals	Large study samples	No downgrade
5. Publication Bias	Funnel plot indicates	No indication of publication bias	No downgrade
Overall Judgment			Moderate quality
6. Exposure-response	Statistically significant trend	All studies show statistically significant exposure-response relations	Upgrade one level
7. Magnitude of effect	Weighted mean r > .5	Weighted mean r = .412 (/ .417 excluding one study)	No upgrade
8. Confounding adjusted	Effect in spite of confounding working towards the nil	No adjustments	No upgrade
**Overall Judgment**			**High quality**

PECO = Population, Exposure, Comparator, Outcome. For explanations, see section S4.

S28. Figure S10 (Funnel plot of noise effects based on the increase of %HA by a 10 dB increase (from 50 to 60 dB $L_{Aeq,24h}$) railway noise in observed data

In order to illustrate the possible bias with respect to OR, Figure S10 shows a funnel plot of the Odds Ratio in relation to the respective standard error, and it can be observed that there is a bias: the distribution of ORs with respect to the standard error is asymmetric and skewed. Studies reporting higher ORs are often associated with high standard errors. It seems that the meta-analysis based on ORs shows an overestimation in the same direction as the comparable analysis based on correlations of raw data.

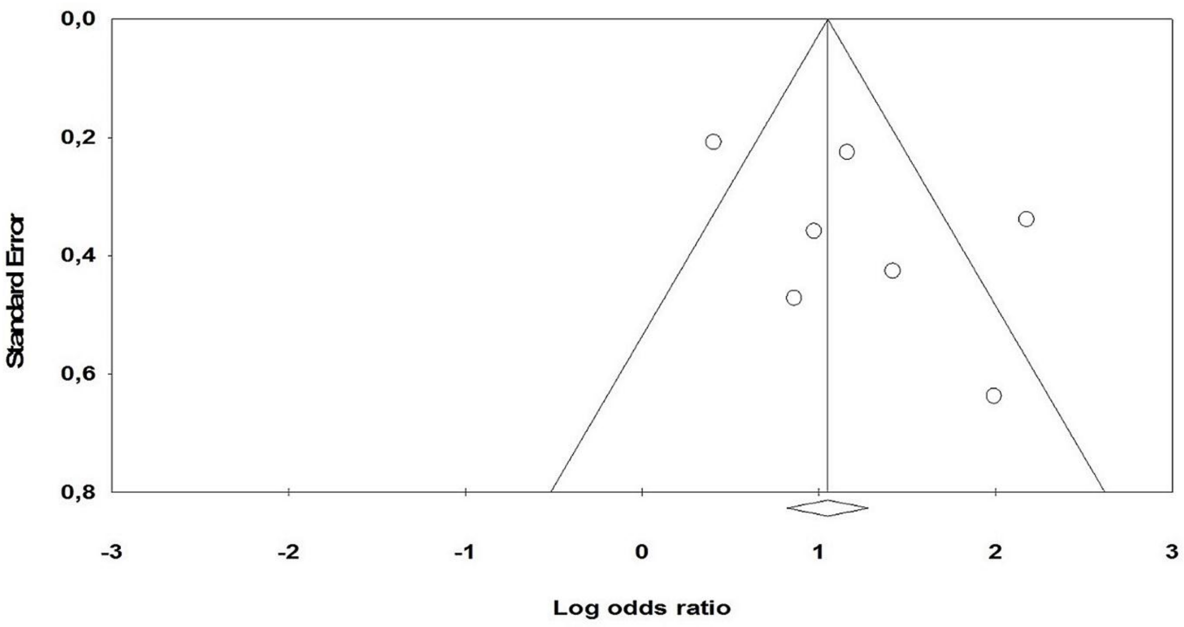

Figure S10. Funnel plot of the meta-analysis of railway noise effects based on the increase of %HA by a 10 dB increase (from 50 to 60 dB $L_{Aeq,24h}$) railway noise in observed data. Odds Ratios are used as effect indicators.

The test on heterogeneity shows statistically significant differences between the seven studies: Q = 24.085; df = 6; p = 0.001; I^2 = 75.088 - which means that a large part of the total variance is due to variance between studies.

S29. Exploring the between-study heterogeneity of Odds Ratios in original grouped data on railway noise annoyance

We explored some study characteristics as potential effect moderators. One of them was the exclusion of the Shinkansen part of the studies by Yano et al. (2005). We performed a meta-analysis of the six railway studies (the original seven, excluding the Yano-Shinkansen study, see Figure S11) and found a small increase of the summary OR (from 3.396 to 4.023) associated with a statistically non-significant heterogeneity test: Q = 9.899; df = 5; p = 0.078, I^2 = 49.489, i.e., the proportion of the total variance, which is due to true variance between studies, has been reduced by excluding the Yano-Shinkansen study. This is in contrast to the meta-analysis of correlations, where no statistically significant reduction of heterogeneity has been observed, when the Yano-Shinkansen study was removed from the dataset.

Study name	Subgroup within study	Statistics for each study				
		Odds ratio	Lower limit	Upper limit	Z-Value	p-Value
Gidloef-G. 2012	Survey1: rail, no vibrations	7,322	2,100	25,528	3,124	0,002
Gidloef-G. 2012	Survey2: rail, noise & vibrations	4,130	1,794	9,509	3,333	0,001
Gidloef-G. 2012	Survey3: rail, many trains	8,813	4,540	17,105	6,431	0,000
Yano 2005	conventional rail	3,191	2,055	4,957	5,165	0,000
Champelovier 2003	rail: France	2,363	0,938	5,953	1,825	0,068
Yokoshima 2008	Shinkansen: Kanagawa	2,645	1,311	5,337	2,716	0,007
		4,023	2,627	6,159	6,404	0,000

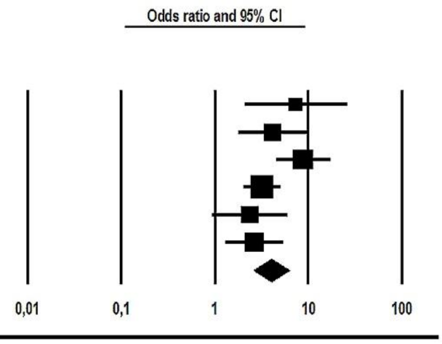

Figure S11. Odds Ratios and 95% confidence intervals from six studies, (based on observed data) for the increase of the rate of “highly annoyed” persons from 50 to 60 dB $L_{Aeq,24h}$ railway noise. The right part of the graph contains a forest plot of the ORs and their respective 95% confidence intervals. The figures of the last row indicate the summary estimates.

We further explored the heterogeneity of railway noise annoyance studies with respect to the ORs referring to the %HA increase at a 50-60 dB level increase by means of a subgroup analysis. Given the requirement of at least three studies in each of the comparison groups, only the noise level range could be used as a potential moderator of the annoyance ORs of %HA due to the 10 dB increase from 50 to 60 dB $L_{Aeq,24h}$.

We divided the group of six studies providing both %HA data at comparable levels as well as noise level range data in two subgroups according to noise level range, a "higher range" (>30 dB) and a "lower range" group (<30dB) and performed a mixed effects meta-analysis. The "high range" group consisted of three studies (France 1997-98, Japanese conventional trains, and Shinkansen Kanagawa). The "low range" group consisted of three Gidlöf studies (no vibration, noise + vibration, and many trains).

Results with respect to noise level range:

- "higher range" (three studies): summary OR = 2.923 (2.069 – 4.132), I^2 = 0.000
- "lower range" (three studies): summary OR = 6.676 (4.134 – 10.781), I^2 0.000
- Q between groups (mixed effect) = 7.497; df = 1; p = 0.006.

The two groups do differ statistically significantly: the ORs for the "lower range" group are considerably higher than for the "higher range" group. We conclude that differences in noise level range explain part of the between-study variance. On the other hand, we should remember that the lower range studies in our analysis all are related to the higher levels, starting at 41 dB, while the "high level range" studies start several decibels lower (e.g., at 24 dB in Japanese conventional trains study). We conclude that differences in level range do explain parts of the between-study variance, but there may be a confounding effect of the absolute position of the study within the range of noise levels.

S30. Meta-analysis of railway noise ORs based on modelled data

Ten of 11 railway noise annoyance studies provided complete modelled data, (i.e., B, the respective standard error calculated from logistic regressions using "highly annoyed" vs. "not highly annoyed" as dependent variable, and the noise exposure level as independent variable: in nine studies L_{den} and in one study $L_{Aeq,24h}$). These data were used in order to calculate ORs referring to the %HA increase per 10 dB level increase. The next meta-analysis is based on these OR estimates (Figure S12).

Study name	Subgroup within study	Statistics for each study				
		Odds ratio	Lower limit	Upper limit	Z-Value	p-Value
Gidloef-G. 2012	Survey1: rail, no vibrations	7,614	2,972	19,507	4,229	0,000
Gidloef-G. 2012	Survey2: rail, noise & vibrations	4,711	2,617	8,482	5,167	0,000
Gidloef-G. 2012	Survey3: rail, many trains	7,614	4,574	12,674	7,808	0,000
Yano 2005	conventional rail	2,841	2,442	3,305	13,529	0,000
Yano 2005	Shinkansen	14,202	7,076	28,502	7,466	0,000
Schreckenb. 2013	rail: Rheintal	3,975	3,405	4,641	17,468	0,000
Champelovier 2003	rail: France	2,014	1,655	2,450	7,000	0,000
Lercher 2008	rail: Tirol, ALPNAP	2,552	2,119	3,075	9,863	0,000
Lercher 2008	rail: Tirol, BBT face-to-face	2,411	2,127	2,733	13,750	0,000
Lercher 2008	rail: Tirol, BBT phone	2,998	2,474	3,633	11,204	0,000
		3,526	2,830	4,393	11,231	0,000

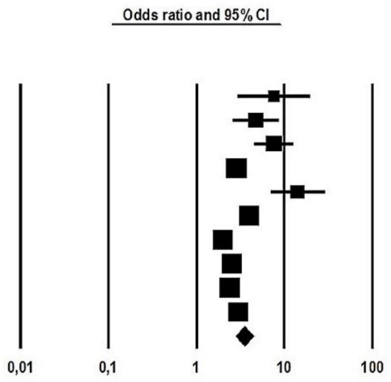

Figure S12. Odds Ratios and 95% confidence intervals (based on modelled data) referring to the %HA increase per 10 dB (L_{den}) increase of railway noise in ten studies. The right part of the graph contains a forest plot of the ORs and their respective 95% confidence intervals. The figures of the last row indicate the summary estimates.

All of the ten studies included show ORs > 1 and are statistically highly significant. The summary OR is 3.526, which is comparable to the summary OR based on observed data. The summary confidence interval ranges from 2.8 to 4.4, which is somewhat smaller than those of the analysis based

on observed data. The first Gidlöf study and the Shinkansen study show the largest confidence intervals. The reasons for large confidence interval in the Gidlöf study are unknown at present; a possible reason in case of the Shinkansen study may be connected to the effect of vibrations on noise annoyance (see S26) and to the large slope of the exposure-response relation found in this study – see section 3.3.1 of this report.

The heterogeneity test is statistically highly statistically significant: Q = 79.894; df = 9; $p < 0.001$. The $I^2 = 88.735$ - which means that about 90 percent of the total variance is due to the variance between studies. We explored the heterogeneity (see S31) and found the range of noise levels to be a candidate for explaining parts of the variance between studies. On the other hand, lower noise level ranges are associated with high noise levels in our sample of studies – this can be seen as a confounding factor. In addition, S31 shows that a part of the heterogeneity between studies decreases slightly, when the Yano/Shinkansen study is excluded from the analysis; the OR decreases, too (from 3.526 to 3.181).

S31. Exploring the between study heterogeneity of Odds Ratios based on modelled data on railway noise annoyance

We explored several study characteristics as potential effect moderators. One of them was the Shinkansen part of the studies by Yano et al., others are shown below. We first performed a meta-analysis of the data set from figure S12 in section S30; excluding the Yano-Shinkansen study (Figure S13).

Study name	Subgroup within study	Statistics for each study				
		Odds ratio	Lower limit	Upper limit	Z-Value	p-Value
Gidloef-G. 2012	Survey1: rail, no vibrations	7,614	2,972	19,507	4,229	0,000
Gidloef-G. 2012	Survey2: rail, noise & vibrations	4,711	2,617	8,482	5,167	0,000
Gidloef-G. 2012	Survey3: rail, many trains	7,614	4,574	12,674	7,808	0,000
Yano 2005	conventional rail	2,841	2,442	3,305	13,529	0,000
Schreckenb. 2013	rail: Rheintal	3,975	3,405	4,641	17,468	0,000
Champelovier 2003	rail: France	2,014	1,655	2,450	7,000	0,000
Lercher 2008	rail: Tirol, ALPNAP	2,552	2,119	3,075	9,863	0,000
Lercher 2008	rail: Tirol, BBT face-to-face	2,411	2,127	2,733	13,750	0,000
Lercher 2008	rail: Tirol, BBT phone	2,998	2,474	3,633	11,204	0,000
		3,181	2,606	3,883	11,372	0,000

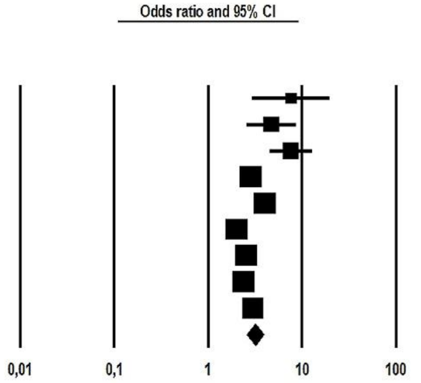

Figure S13. Odds Ratios and 95% confidence intervals (based on modelled data) referring to the %HA increase per 10 dB (L_{den}) increase of railway noise in 9 studies (excluding the Yano-Shinkansen study). The right part of the graph contains a forest plot of the ORs and their respective 95 % confidence intervals. The figures of the last row indicate the summary estimates.

It turned out that both the summary OR (from 3.526 to 3.181) and the heterogeneity decreased. However, there is still a statistically highly significant heterogeneity (Q = 59.502, df = 8; $p < 0.001$; $I^2 = 86.555$).

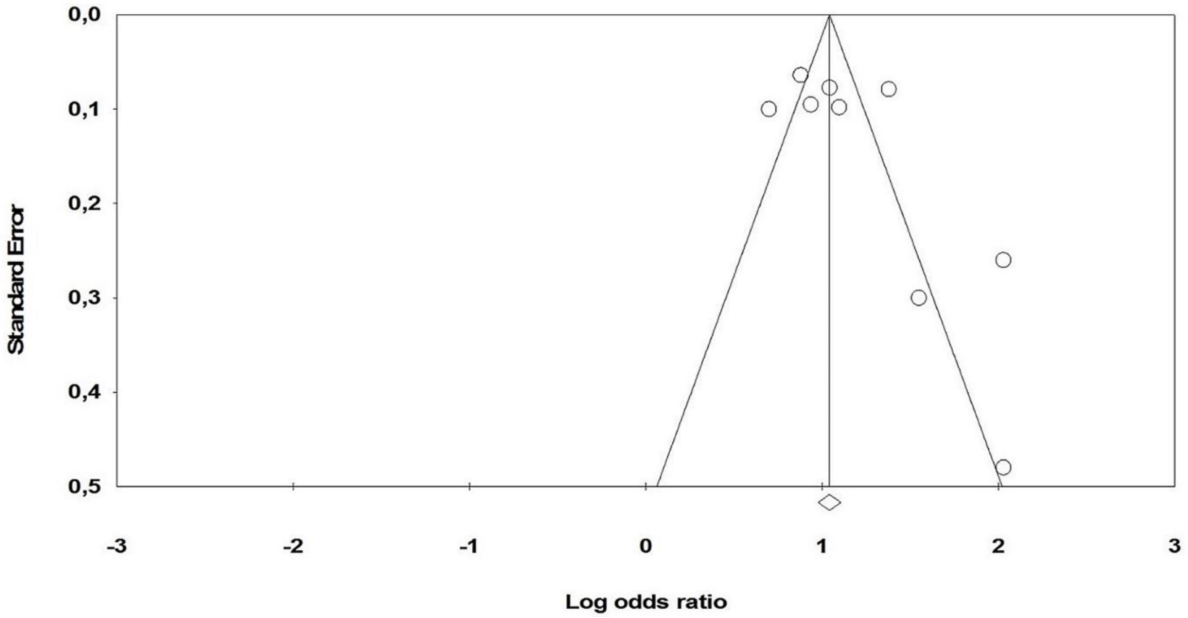

Figure S14. Funnel plot of the meta-analysis of railway noise effects (excluding the Yano-Shinkansen study) based on the increase of %HA by a 10 dB ($L_{Aeq,24h}$) increase of railway noise in modelled data. Odds Ratios are used as effect indicators**.**

The funnel plot (Figure S14) of the meta-analysis shown in Figure S13 shows a definite asymmetry: the three Gidlöf studies show the largest effects and the highest standard errors. This may be due to study characteristics, which could not be analyzed here, but it may also be due to a publication bias.

We further explored the heterogeneity of railway noise annoyance studies with respect to OR representing the %HA increase per 10 dB level increase using modelled data by means of subgroup analyses. Given the requirement of at least three studies in each of the comparison groups and no missing data within a group, study quality, noise level range, response rate, and response scale type could be used as potential effect moderators.

With respect to the study quality, nine studies were available providing modelled data without missings. They were divided into two groups: "higher quality" (six studies, consisting of two Gidlöf studies, the Rhine valley study, and three Alpine studies), and "lower quality" (three studies, consisting of the Gidlöf study without vibrations, the Japanese conventional trains study, and France 1997-98).

Results with respect to study quality:

- "higher quality" (six studies): summary OR = 3.424 (2.657 – 4.413); I^2 = 88.168
- "lower quality" (three studies): summary OR = 2.775 (1.884 – 4.087); I^2 = 84.283
- Q between groups (mixed effect) = 0.793; df = 1; p = 0.373.

The two groups do not differ statistically significantly: the ORs of the "higher quality" group are similar to those of the "lower quality" group.

With respect to the noise level range, we divided the group of six studies providing both modelled data without missings as well as noise level range data in two subgroups according to noise level range (a "higher range" (>30 dB L_{den}) and a "lower range" group (<30dB L_{den})) and performed a mixed effects meta-analysis. The "high range" group consisted of three studies (France 1997-98, the Rhine valley study, and Japanese conventional trains). The "low range" group consisted of three Gidlöf studies (no vibration, noise and vibration, and many trains).

Results with respect to noise level range:

- "higher range" (three studies): summary OR = 2.845 (1.974 – 4.100); I^2 = 93.113
- "lower range" (three studies): summary OR = 6.383 (4.469 – 9.116), I^2 = 0.000
- Q between groups (mixed effect) = 9.631; df = 1; p = 0.002.

Similar to the results using observed data (sections 3.3.3.1 and S30), the two groups differ statistically significantly: the ORs for the "lower range" group are considerably higher than for the "higher range" group. We conclude that differences in noise level range explain part of the between-study variance with respect to ORs from modelled data. On the other hand, the same caution should be taken as in the former section with respect to the interpretation: the "lower range" studies in our analysis are all related to the higher levels, and the "higher level range" studies start several decibels lower. We conclude that differences in level range do explain parts of the between-study variance, but there may be a confounding effect of the absolute position of the study within the range of noise levels.

With respect to response rate, we divided the set of eight studies providing both the response rate as well as the %HA difference from modelled data in two subgroups according to "high response rate" (>50%) and "low response rate" (<50%), and performed a mixed effects meta-analysis. Five studies reported a "high response rate" (three of the Gidlöf studies, one Alpine study, and the Japanese conventional trains study). Three studies reported a "lower response rate" (two Alpine studies, and the Rhine valley study).

Results with respect to response rate:

- "high response rate" (five studies): summary OR = 3.817 (2.730 – 5.337); I^2 = 85.437
- "low response rate" (three studies): summary OR = 3.135 (2.400 – 4.094), I^2 = 85.317
- Q between groups (mixed effect) = 0.811; df = 1; p = 0.368.

The two groups do not differ statistically significantly, although there is a tendency for higher ORs at low response rates. On the other hand, the group of "low response rate" studies is very small. We conclude that differences in response rate do not explain much of the between-study variance.

The last dimension which can be used as an effect moderator is the response scale type: We divided the set of nine studies providing the necessary data into two subgroups according to "numerical scale" (six studies, 11 scale steps) and "verbal scale" (three studies, 5 scale steps). The "numerical scale" group consisted of France 1997-98, three Gidlöf studies, one Alpine study, and the Japanese conventional trains study. The "verbal" group consisted of two Alpine studies, and the Rhine valley study.

Results with respect to response scale type:

- "numerical" (six studies): summary OR = 3.281 (2.478 – 4.345); I^2 = 85.815
- "verbal" (three studies): summary OR = 3.135 (2.400 – 4.094); I^2 = 85.317
- Q between groups (mixed effect) = 0.053; df = 1; p = 0.817.

The two groups do not differ statistically significantly. We conclude that differences in response scale type do not explain much of the between-study variance.

S32. Grading the evidence of Odds Ratios representing the %HA increase per 10 dB level increase of railway noise

Similar arguments as posed in section S16 (with respect to the road traffic noise annoyance evidence based on correlations) can be posed with respect to the evidence based on ORs referring to the %HA increase at 10 dB increase of railway noise level: **study limitations** have been taken into account as far as possible, and the confounding of level range restriction with the mean height of the levels have been discussed. The **inconsistency of results** is restricted to the height of ORs. All ORs are > 1 and those based on modelled data are statistically highly significant; most of the ORs based on observed data are statistically significant, too. With respect to the **indirectness of evidence**, we do not see relevant differences between the population and the sample of participants included in the studies. **Imprecision** is no problem, since we deal with sample sizes from about 500 to 2,000 participants. With respect to **publication bias**, we observed an asymmetry of ORs based on modelled data, which might be due to a publication bias.

In sum, we are confident in the evidence of a statistically significant increase of %HA with a 10 dB increase of railway noise levels, but there might be a certain overestimation of the effect, especially with modelled data. In terms of the GRADE system, we assigned "moderate quality" to the effects

based on original grouped data (Table S12), but "high quality" to the effects based on modelled data (Table S13).

Table S12. GRADE summary of findings for the quality of evidence related to railway noise and percent of highly annoyed persons. Health outcome: OR referring to the %HA increase per 10 dB level increase (50-60 dB L_{den}), based on original grouped data, seven studies.

Domains	Criterion	Assessment	Grading
Start Level	Study design: cross-sectional = high quality	High quality	High quality
1. Study Limitations	Quality of majority of studies (risk of bias)	High quality of majority of studies	No downgrade
2. Inconsistency	Conflicting results; high I2	High between study variance	Downgrade one level
3. Directness	Direct comparison; same PECO	Same PECO	No downgrade
4. Precision	Small sample sizes OR Low numbers of events (HA) OR Wide confidence intervals	Large study samples	No downgrade
5. Publication Bias	Funnel plot indicates	Small publication bias	Downgrade one level
Overall Judgment			Low quality
6. Exposure-response	Statistically significant trend	5 of the 7 studies show statistically significant ORs	No upgrade
7. Magnitude of effect	Weighted mean OR > 2.5	Weighted mean OR = 3.396 (4.023 when one study is excluded)	Upgrade one level
8. Confounding adjusted	Effect in spite of confounding working towards the nil	No adjustments	No upgrade
**Overall Judgment**			**Moderate quality**

PECO = Population, Exposure, Comparator, Outcome.
OR = 2.5 converted to Cohen's [9] d = 0.5 = medium effect.

Table S13. GRADE summary of findings for the quality of evidence related to railway noise and percent of highly annoyed persons. Health outcome: OR referring to the %HA increase per 10 dB level increase, based on modelled data, ten studie**s.**

**Domains**	**Criterion**	**Assessment**	**Grading**
Start Level	Study design: cross-sectional = high quality	High quality	High quality
1. Study Limitations	Quality of majority of studies (risk of bias)	High quality of majority of studies	No downgrade
2. Inconsistency	Conflicting results; high I2	High between study variance	Downgrade one level
3. Directness	Direct comparison; same PECO	Same PECO	No downgrade
4. Precision	Confidence interval contains 25% harm or benefit and no effect OR optimal information size reached	Large study samples	No downgrade
5. Publication Bias	Funnel plot indicates	Probable publication bias	Downgrade one level
Overall Judgment			Low quality
6. Exposure-response	Statistically significant trend	All of the studies show statistically significant ORs	Upgrade one level
7. Magnitude of effect	Weighted mean OR > 2.5	Weighted mean OR = 3.526 (3.181 when one study is excluded)	Upgrade one level
8. Confounding adjusted	Effect in spite of confounding working towards the nil	No adjustments	No upgrade
**Overall Judgment**			**High quality**

PECO = Population, Exposure, Comparator, Outcome.
OR = 2.5 converted to Cohen's [9] d = 0.5 = medium effect.

S33. The influence of co-determinants in railway noise annoyance studies

As stated in section 3.1.7 in the main paper, individual noise annoyance judgments of residents are to a large extent influenced by personal variables (e.g., noise sensitivity and coping capacity). These individual within-study variables are not discussed here. Instead, we like to mention between-study co-determinants which apply to many residents and should be taken into account when analyzing noise annoyance from railway noise:

a) Ground-borne vibrations are sometimes an additional exposure variable in railway noise situations, which may be difficult to separate from noise effects. Gidlöf-Gunnarsson et al. [16, p.191] report that "In Sweden, about 141 km railway lines with approximately 6, 560 dwellings are estimated to be exposed to ground-borne vibrations induced by trains that exceed 0.35 mm/s and about 920 dwellings with a vibration velocity that exceeds 1.4 mm/s inside the dwelling." Schomer et al. [17] suggest that in six railway studies subject to vibration, the %HA at L_{dn} levels >65 dB was about 20% higher as compared to seven studies where vibration seemingly did not play a role. Vibrations have been reported to cause a number of effects, such as fear of damage to the house and irritations due to household items rattling. In the set of 11 studies included in our review, two studies explicitly mention ground-borne vibrations as an additional source of annoyance. One of these studies was excluded from the estimation of a

new railway noise annoyance exposure-response relation due to reasons explained in 3.3.1.1 of the main text.

b) The distance between residential buildings and railway tracks may differ between studies and locations. For instance, the distance between railway tracks and residential buildings in Europe is usually larger than in Asia. Sato, Yano and Morihara [18] found the noise annoyance ratings of residents living close to the railway tracks (< 80 m) to be higher than the ratings from residents living somewhat farther away (> 80 m), even at comparable noise levels. Lim, Kim, Hong and Lee [19] report a similar observation from Vietnam. The distance effect on noise annoyance may be due to different reasons: (a) an interaction with vibrations (buildings closer to the tracks are more prone to vibrations), (b) the increasing risk of an accidental damage to the house with decreasing distance to the tracks, and (c) the acoustic effect of higher rise-times for noise levels due to different shielding effects of neighbor houses: at close distance to the tracks, close neighbor houses may shield train noise quite effectively during approach and receding, but close neighbor houses do not shield the noise when the train runs directly in front of the own house, causing an annoying surprise effect when the train leaves the acoustic shield.

c) The main construction type of residential buildings may differ between studies and locations. As noted by Sato, Yano, Björkman and Rylander [20], different house structures (apartment vs. detached houses) had an influence on road traffic noise annoyance, with people living in detached houses being more often subject to exhaust fumes and vibrations as compared to people living in apartments. The same might be true with respect to noise from freight trains using diesel engines. In addition, it has been noted that traditional Japanese houses are made from wood and are more subject to vibrations than are concrete buildings.

d) The relation between passenger trains and freight trains may differ between studies (and study areas) and have an influence on noise annoyance. In Europe, passenger and freight trains share some railway routes – sometimes even on the same tracks. Generally, there are more passenger trains at daytime and more freight trains at night. In such cases, residents living close to the tracks can rarely enjoy a quiet period during the 24-hours of a day, and often, nighttime noise from freight trains is louder than daytime noise from passenger trains. Residents show higher noise annoyance ratings to freight trains as compared to passenger trains (e.g., Zeichart, Sinz, Schweiger, Kilcher and Herrmann [21]), and one of the reasons for this difference is attributed to the long duration of freight train sounds as well as the composition of the sound (e.g., more rattles with freight trains).

e) The relation between conventional passenger trains and high-speed passenger trains may influence noise annoyance judgments of residents. This seems to be especially true for the Shinkansen trains in Japan, as reported by Yano et al. [15].

f) Availability of a quiet façade: Similar to the results often reported with respect to road traffic noise (e.g., De Kluizenaar et al. [11]), the availability of a quiet façade at home is associated with less railway noise annoyance (Schreckenberg, [22]).

g) Environmental conditions: Similar to the study moderators discussed in S23 with respect to road traffic noise, environmental conditions, like flat terrain vs, valleys, and high vs. low prevalence of air-conditioning at homes, should be taken into account with respect to railway noise annoyance, too.

S34. Wind turbine noise effects on annoyance

As stationary industrial sound sources, wind turbine noise can be handled in section 3.6 (S36) of this report. However, wind turbines noise is given an own section here, because it is "special" in several respects: wind turbines often emit a repetitive "swooshing" sound of the blades, which attracts much attention; wind turbines often generate lower frequencies of sound than traffic, and wind turbines are usually located in less densely populated areas, where residents may expect quietness. In addition, the aggregation of wind turbines into "wind parks" is a relatively recent innovation, which has two consequences: (a) residents often experience a "change" situation – which

usually is associated with an increase of annoyance, and (b) the body of peer-reviewed research addressing the potential impacts of wind turbine noise is sparse.

The two publications included in the wind turbine noise annoyance analysis contain descriptions of a total of four individual studies, performed between 2000 and 2012, and with sample sizes from about 350 to 754 (a total of 2,481 respondents). The first publication (Janssen et al., [26]) includes two studies from Sweden and another one from The Netherlands and is backed up by several original publications, which were used in order to extract additional information for the review. The second publication (Kuwano et al. [23]) reports on a single study in Japan. All four studies used measurements in the vicinity of the respondents' addresses and estimated the respondents' annual L_{den} according to national standards and a procedure described by Van den Berg et al. [24]. The three European studies used special annoyance questions (without reference to a time frame) and distinguish between indoor and outdoor, while the Japanese study used the regular ICBEN question (12 months, with reference to "here at home"). Based on the observation by Wirth, Brink & Schierz [27] that annoyance responses based on the unspecified situation ("here at home") are more or less the same as those based explicitly on the outdoor situation ("outside of the house"), we decided that the outdoor and ICBEN questions are roughly comparable. The 5-point verbal response scales in the European studies uses a filter in the first step (1 = "not noticed") and proceeds with 2 = "noticed, but not annoyed", 3 = "slightly annoyed," 4 = "rather annoyed," and 5 = "very annoyed." The 5-point verbal scale in the Japanese study (Kuwano et al. [23]) completely follows the ICBEN proposal (1 = "not at all ", 2 = "slightly", 3 = "moderately", 4 = "very", 5 = "extremely" annoyed), but includes an additional option 9 = "inaudible". We decided to take the four annoyance steps in the European scale and the five annoyance steps in the Japanese study to represent the available range of potential annoyance. Both scales may be used for the comparison of raw score correlations between noise levels and annoyance judgments. Table S14 shows an abbreviated list of study data on wind turbine noise annoyance.

Table S14. Wind turbine noise studies included

**Publication** (see S3 for references)	**Location**	**Year data**	**Sample type**	**Type of survey**	**Sample size**	**Noise level descriptors**	**Noise level range L_{den}**	**Annoyance Scale**	**Remarks**	**Study Quality Rating**
Janssen et al. 2011 / Pedersen & Persson-Waye 2004	Sweden	2000	Stratified (distance)	postal	351	L_{den} *	29 - 50	Notice filter & 4-p verbal scale (inside & outside)	Flat terrain	23
Janssen et al. 2011 / Pedersen & Persson-Waye 2007	Sweden	2005	Stratified (distance)	postal	754	L_{den} *	29 - 50	Notice filter & 4-p verbal scale (inside & outside)	Mixed terrain	23
Janssen et al. 2011 / Van den Berg et al. 2008	The Netherlands	2007	Stratified (noise level)	postal	725	L_{den}	29 - 50	Notice filter & 4-p verbal scale (inside & outside)	Flat terrain (rural vs. built-up	23
Kuwano et al. 2014	Japan	2010 - 2012	Stratified	Face-to-face	651	L_{night} L_{dn}	31-56	5-point ICBEN scale	Rural areas	18

* The two Swedish studies were initially published including L_{dn} values. These values were recalculated and converted to L_{den} by Janssen et al. [26].

S34.1. Wind turbine noise effects 1: exposure-response relations

Although exposure-response functions for L_{den} were provided by the authors of all four wind turbine noise studies, we decided not to combine them, because the construction of the Japanese function differed from the one related to the "Nordic" studies. There is already a common exposure-response curve published for the three "Nordic" studies (Janssen et al. [26] – by means of Multilevel Grouped Regression), and Kuwano et al. [23] published a separate curve for the Japanese study – estimated by means of logistic regression). We include both curves here with small graphic amendments (Figure S15), show an overlay of the two (Figure S16), and provide a narrative comparison.

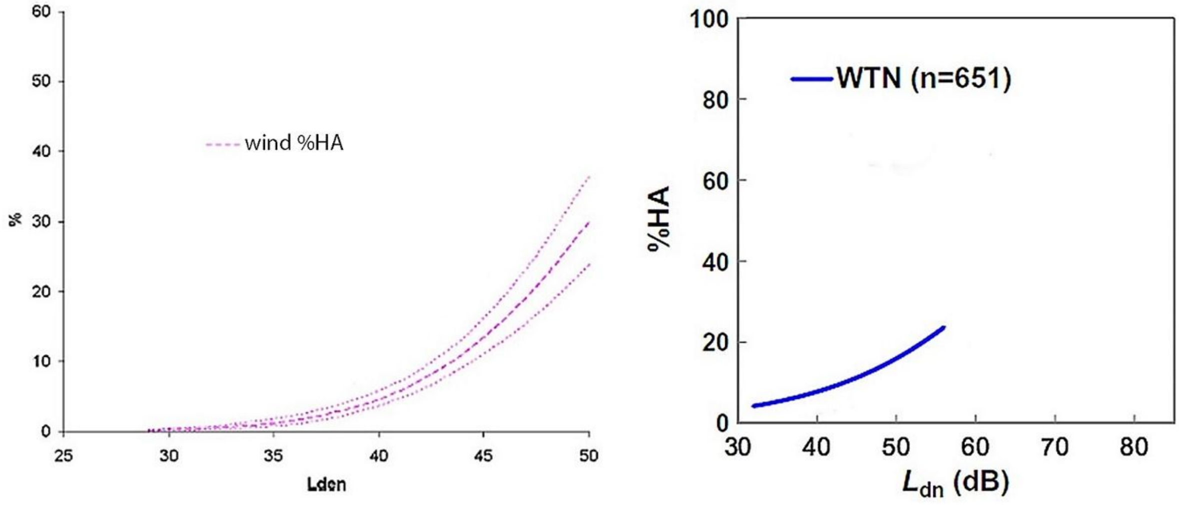

Figure S15. Exposure-response graphs for wind turbine noise annoyance, adapted from Janssen et al. [26] (left, outdoors only) and Kuwano et al. [23] (right). The original graphs contain additional curves which are deleted here. "WTN" = Wind Turbine Noise.

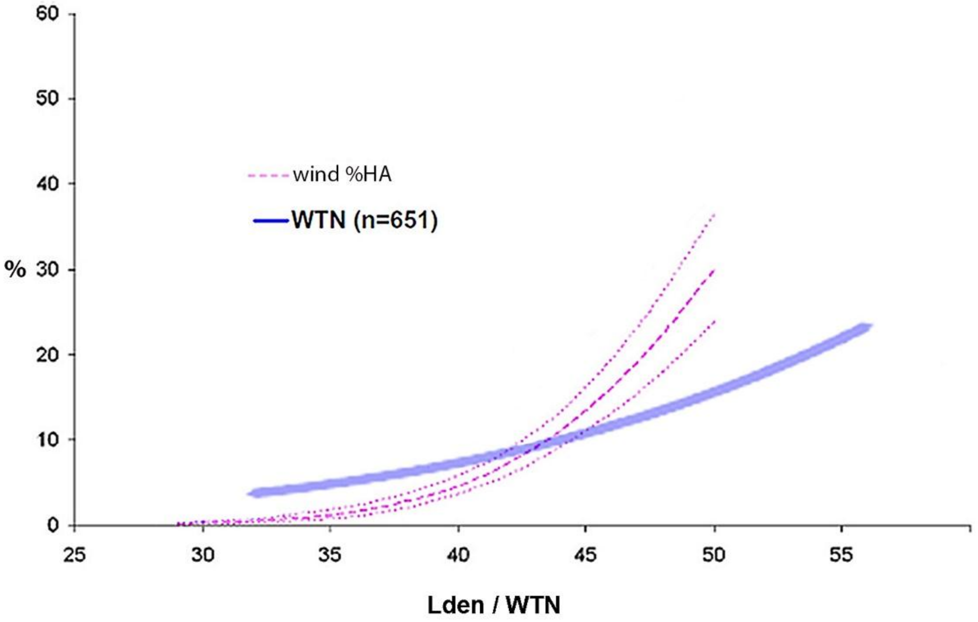

Figure S16. Overlay of the two wind turbine annoyance graphs adapted from Janssen et al. [26] (red lines) and Kuwano et al. [23] (blue line). The Kuwano et al. curve is based on L_{dn}, no correction for Lden has been applied. "WTN" = Wind Turbine Noise.

It is obvious that the Japanese exposure-response curve has somewhat higher percentages of HA at lower noise levels as well as lower percentages at higher noise levels. Unfortunately, the publications of the Japanese wind turbine study do not contain any confidence intervals – which hinders drawing conclusions about the overlap between the Japanese and the European studies. However, it seems likely that a combination of both curves including a proper weighting according to sample size will at least reduce the steep increase of the %HA at higher noise levels evident in the European studies. However, the fact that both curves are based on different regression models complicates such a combination of the curves.

Although our data base for a exposure-response relation between wind turbine noise levels and wind turbine noise annoyance is very small (two publications including 4 studies) and may not be representative for all residential areas exposed to wind turbines, it is remarkable that the percentage of highly annoyed residents in our sample is rather high, given the relatively low noise levels. There are almost 14 %HA at 47.5 dB L_{den}/L_{dn}. Some explanations for these results are presented in section S34.6.

S34.2. Grading the evidence for wind turbine noise annoyance with respect to exposure-response curves

Given the small number of wind turbine noise annoyance studies included here, it may be misleading to grade the quality of evidence in full detail, but we feel almost confident with the general observation of a monotone relationship between wind turbine noise levels and the percentage of respondents highly annoyed by wind turbine noise. On the other hand, the two publications used here provide exposure-response curves which differ with respect to form and slope. The confidence in the quality of evidence with respect to the effects of wind turbine noise on the percentage of highly annoyed residents may be decreased for several reasons, including

- **Study limitations**: Research on the effects of wind turbine noise on residents in the vicinity of wind turbines is confined to observational studies. These have been done by means of two different methods of participant selection and two different survey types, but similar noise exposure assessments. We have taken the study limitations into account by grading the quality of each study selected.
- **Inconsistency of results**: As observed above, the two exposure-response curves differ in form and slope, and it seems impossible to aggregate the two into one common curve.
- **Indirectness of evidence**: Differences between the population and the samples included in the studies were not reported, and we do not see relevant differences between the population and the samples, except with respect to the age range: none of the studies includes children – this is a characteristic shared by all surveys presented in this review.
- **Imprecision**: In view of the total sample sizes of the studies reported here (from 351 to 754 participants), imprecision should not be a serious general problem, but the number of respondents at certain noise levels is rather small. For instance, the majority of respondents in the two Swedish studies were exposed to 35-40 dB, and levels <35 and >45 dB were rarely filled in these two studies. The Japanese and Dutch studies both report sufficient respondents at levels from 31 to 45 dB, but levels outside this range were rare.
- **Publication bias**: All of the studies selected are journal publications. This may be prone to publication bias, because authors and journals may find it easier to publish large effects as compared to small effects.

The summary of our evidence grading is shown in Table S15.

Table S15. GRADE summary of findings for the quality of evidence related to wind turbine noise and degree of annoyance. Health outcome based on published exposure-response curves, two publications.

Domains	Criterion	Assessment	Grading
Start Level	Study design: cross-sectional = high quality	High quality	High quality
1. Study Limitations	Quality of majority of studies (risk of bias)	High quality of majority of studies	No downgrade
2. Inconsistency	Conflicting results; high conflict	High between study variance	Downgrade one level
3. Directness	Direct comparison; same PECO	Same PECO	No downgrade
4. Precision	Small sample sizes OR Low numbers of events (HA) OR Wide confidence intervals	Low number of respondents at upper noise levels	Downgrade one level
5. Publication Bias	Funnel plot	Not applicable	No downgrade
Overall Judgment			Low quality
6. Exposure-response	Monotone trends	Form and slope of curves differ between the two publications	No upgrade
7. Magnitude of effect	Weighted mean OR > 2.5 OR Weighted mean r > .5	Not applicable	No upgrade
8. Confounding adjusted	Effect in spite of confounding working towards the nil	No adjustments	No upgrade
Overall Judgment			Low quality

PECO = Population, Exposure, Comparator, Outcome. For explanations, see section S4.

S34.3. Wind turbine noise effects 2: Correlations with annoyance raw data

All four studies provided correlations between L_{den} or L_{dn} and wind turbine noise annoyance scores. Unfortunately, only point-biserial correlations between L_{den} and HA were available for the three European studies at the time of our inquiry, i.e., L_{den} was used as a continuous variable and HA as a dichotomous variable ("very annoyed"). The Japanese study provided a Pearson correlation using noise levels and annoyance as continuous variables. The point-biserial correlation coefficients were transformed to biserial correlations according to the formula given by Terrel [28], and all four correlations were (together with the respective n) subjected to a meta-analysis. It should be noted that biserial and point-biserial correlations are highly sensitive to the frequency distribution of the two groups in the dichotomous variable, leading to decreasing correlations with increasing disproportionality between the number of cases belonging to one or the other group.

Study name	Subgroup within study	Correlation	Lower limit	Upper limit	Z-Value	p-Value
Yano et al 2013/ Kuwano et al 2014	Japan	0,130	0,054	0,205	3,328	0,001
Janssen 2011/Van den Berg et al 2008	NL2007	0,354	0,178	0,508	3,819	0,000
Janssen 2011/Pedersen ...2007	Sweden 2005	0,211	-0,008	0,410	1,891	0,059
Janssen 2011/Pedersen ...2004	Sweden 2000	0,464	0,259	0,629	4,142	0,000
		0,278	0,111	0,430	3,214	0,001

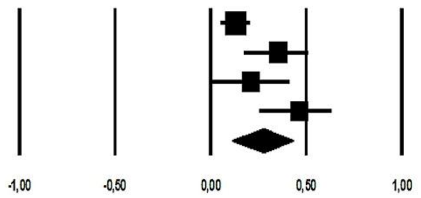

Figure S17. Meta-analysis of four studies using correlations between Lden or Ldn levels and high wind turbine noise annoyance. The right part of the graph contains a forest plot of the correlations and their respective 95% confidence intervals. The figures of the last row indicate the summary estimates.

The four correlations may be split into two virtual groups, according to their size: the Japanese and the Swedish study from 2005 show correlations from 0.13 to 0.21, while both of the other two studies (S 2007 and NL 2007) show correlations above 0.35. The summary correlation of the meta-analysis (which attributes some weight according to the sample size of the studies) is somewhat lower than the corresponding correlations from transportation noise annoyance studies. The summary correlation (Figure S17) is r = 0.278; p = 0.001; 95% CI = 0.11 – 0.430. The test of heterogeneity is statistically highly significant: Q = 12.393; df = 3; p = 0.006, and I^2 = 75.792 – which means that about 76 percent of the total variance is due to the variance between studies.

The relatively low summary correlation is an indication of rather low common variance, which might be interpreted in four different ways:

- The acoustical description of wind turbine noise by means of L_{den} or L_{dn} is a poor predictor of (high) wind turbine noise annoyance in the four studies used here,
- The assessment of (high) wind turbine noise annoyance by means of the questions and response options used here has a poor relation to the noisy properties of the wind turbine sounds,
- The causal relation between wind turbine noise levels and (high) wind turbine noise annoyance is rather weak in the four studies used here, or
- The three studies which reported point-biserial correlation coefficients had rather low proportions of highly annoyed participants. This might have contributed to low correlation coefficients.

At present, there is neither a means to decide whether these four options are exhaustive nor to decide between them or attribute weights to each of them. In any case, the different potential interpretations together with the results of section S34.1 show that there is a need to establish common protocols for future wind turbine noise annoyance studies.

S34.4. Grading the evidence for wind turbine noise annoyance with respect to correlations

Given the small number of wind turbine noise annoyance studies included here, the precautions presented in section S34.2 apply here as well. The confidence in the quality of evidence with respect to the effects of wind turbine noise on the level of residential annoyance may be decreased for several reasons, including

- **Study limitations**: We have taken the study limitations into account by grading the quality of each study selected.
- **Inconsistency of results**: The meta-analysis of the four studies based on correlations reveals a somewhat lower summary correlation as compared to those in transportation noise annoyance studies, even though most studies show statistically highly significant positive correlations between noise level and annoyance.
- **Indirectness of evidence**: We do not see relevant differences between the population and the samples, except with respect to the age range.

- **Imprecision**: Imprecision should not be a serious problem, given the total sample sizes of the respective studies.
- **Publication bias**: There may be a publication bias due to the typical journal policy to prefer publishing large effects.

In sum, we are moderately confident in the evidence with respect to correlations between wind turbine noise levels and wind turbine noise annoyance, and we like to assign the grade "moderate quality" (Table S16).

Table S16. GRADE summary of findings for the quality of evidence related to wind turbine noise and degree of annoyance. Health outcome based on correlations, four studies.

Domains	Criterion	Assessment	Grading
Start Level	Study design: cross-sectional = high quality	High quality	High quality
1. Study Limitations	Quality of majority of studies (risk of bias)	High quality of majority of studies	No downgrade
2. Inconsistency	Conflicting results; high I2	High between study variance	Downgrade one level
3. Directness	Direct comparison; same PECO	Same PECO	No downgrade
4. Precision	Small sample sizes OR Low numbers of events (HA) OR Wide confidence intervals	Large study samples	No downgrade
5. Publication Bias	Funnel plot indicates	Not applicable	No downgrade
Overall Judgment			Moderate quality
6. Exposure-response	Statistically significant trend	3 of 4 studies show statistically significant exposure-response relations	No upgrade
7. Magnitude of effect	Weighted mean r > .5	Weighted mean r = .278	No upgrade
8. Confounding adjusted	Effect in spite of confounding working towards the nil	No adjustments	No upgrade
**Overall Judgment**			**Moderate quality**

PECO = Population, Exposure, Comparator, Outcome. For explanations, see section S4.

S34.5. Wind turbine noise effects 3: Increase of %HA per 5 dB noise level increase

In contrast to the preceding sections on the effects of a 10 dB (50-60 dB) increase of noise levels, we asked the authors of wind turbine noise studies to provide %HA data for a 5 dB increase from 42.5 to 47.5 dB L_{den} or L_{dn}. The change to smaller differences and smaller levels is due to the fact that residential areas exposed to more than 50 dB L_{den} wind turbine noise are very rare. Accordingly, the

authors of the four wind turbine noise studies provided data for the increase of %HA with an increase of 5 dB L_{den} (42.5 – 47.5). It should be noted that the definition of "highly annoyed" is slightly different between the three European studies and the study from Japan: while the European studies use the top category ("very annoyed", i.e., the top 25% of the 4-point annoyance scale), the Japanese study uses the average of the two top categories of the 5-point ICBEN scale ("very annoyed" and "extremely annoyed"). This procedure was found to represent the top 27% of the annoyance scale (Nguyen et al. [29]).

Table S17 shows the number of participants exposed to the two noise level categories, the %HA at each level as well as the difference in %HA between the two levels. In case of the three European studies the percentages are given both for indoor and outdoor annoyance; in case of the Japanese study, the percentages are given for "here at home", which is seen to be related more to outdoor than to indoor situations (see section S34.1). It is evident that the variance in %HA at comparable noise levels is greater for outdoors than for indoors, especially for the (rather small) first Swedish study.

Table S17. Percent "Highly Annoyed" at 42.5 and 47.5 dB noise levels in four different wind turbine noise studies.

**Study** (see S3 for references)	**Noise level category (dB L_{den} / L_{dn})**	**N per noise level category**	**% Highly Annoyed Indoors**	**% Highly Annoyed Outdoors**	**%HA-Difference at 5 dB Level Difference Indoors**	**%HA-Difference at 5 dB Level Difference Outdoors**
Sweden 2000	42.5	100	5	13	11	19
	47.5	19	16	32		
Sweden 2005	42.5	156	0	3	0	-3
	47.5	12	0	0		
Netherlands	42.5	160	3	6	7	7
2007	47.5	94	10	13		
Japan	42.5	212	--	8.4		2.5
2010-2011	47.5	193	--	10.9		
Mean %HA difference between 47.5 and 42.5 dB L_{den}/L_{dn}					4.50	6.375

Using the "outdoor" data for the European studies, the %HA increase varies from -3% (Sweden 2005) to 19% (Sweden 2000). The average increase is 6.375%. Since no participant in the Sweden 2005 study was highly annoyed at 47.5 dB, a formal meta-analysis of the ORs in the four studies including one with a zero entry would require a correction for the zero rate. In this case, the results of the analyses would heavily depend on the choice of the correction procedure. Different procedures (see e.g., Fleiss & Berlin [30]; Sweeting et al. [31]) produce divergent results. Furthermore, independent of the choice of the correction procedure, the results of the correction in meta-analyses are questionable, if the level of the compared rates is low – especially if the rates are calculated from a small data base (e.g., n ≈ 10). In such cases, the direction of an effect may be changed as a consequence of the correction. In view of such problems, we resigned the analysis of four studies, and we did not expect reliable results from a formal analysis of three studies either. Therefore, no formal meta-analysis of the %HA increase with 5 dB L_{den} increase is presented here.

S34.6. Grading the evidence for wind turbine noise annoyance with respect to an increase of %HA per 5 dB noise level increase

Given the small number of wind turbine noise annoyance studies included here, the precautions presented in section S34.2 apply here, too. The degree of between-study inconsistency is high with respect to the reported increase of %HA with an increase of noise levels: there is a large variation of

effects size, and in addition, the direction of the change in %HA is not consistent in all of the studies. The quality of evidence with respect to the effects of wind turbine noise on the level of residential annoyance is low due to several reasons, including

- **Study limitations**: We have taken the study limitations into account by grading the quality of each study selected.
- **Inconsistency of results**: The increase of %HA with an increase of 5 dB shows a large variation between studies (-3 to 19%), and there is no common systematic trend: three of four studies show an increase of %HA with higher exposure, while one study shows a decrease.
- **Indirectness of evidence**: We do not see relevant differences between the population and the samples, except with respect to the age range.
- **Imprecision**: While the overall sample sizes are sufficiently large, the number of participants exposed to 47.5 dB is low in two of the four studies.
- **Publication bias**: There may be a publication bias due to the typical journal policy to prefer publishing large effects.

In sum, we are not confident with the evidence for the increase of %HA with increasing noise levels. Taken together, we like to assign the grade "low quality" (Table S18).

Table S18. GRADE summary of findings for the quality of evidence related to wind turbine noise and degree of annoyance. Health outcome based on increase of %HA with increase of noise levels studies.

Domains	Criterion	Assessment	Grading
Start Level	Study design: cross-sectional = high quality	High quality	High quality
1. Study Limitations	Quality of majority of studies (risk of bias)	High quality of majority of studies	No downgrade
2. Inconsistency	Conflicting results; high I2	High between study variance	Downgrade one level
3. Directness	Direct comparison; same PECO	Same PECO	No downgrade
4. Precision	Small sample sizes OR Low numbers of events (HA) OR Wide confidence intervals	Small study samples for each of the groups compared	Downgrade one level
5. Publication Bias	Funnel plot indicates	Not applicable	No downgrade
Overall Judgment			Low quality
6. Exposure-response	Statistically significant trend	No systematic trend: 3 of 4 studies show an increase of %HA with higher exposure, 1 study shows a decrease	No upgrade
7. Magnitude of effect	Differences between different exposure groups	Small differences between the groups of low and high exposure	No downgrade
8. Confounding adjusted	Effect in spite of confounding working towards the nil	No adjustments	No downgrade
Overall Judgment			Low quality

PECO = Population, Exposure, Comparator, Outcome. For explanations, see section S4.

S34.7. The influence of co-determinants in wind turbine studies

Similarly to the situation with other noise sources, individual personal within-study factors, like noise sensitivity, age, and coping capacity, influence the annoyance due to wind turbine noise.

Beside these personal factors we like to mention some co-determinant variables, which may differ between wind turbine studies:

- Visibility of the source: being able to see one or more wind turbines from within the home (e.g., from the living room) is associated with increased wind turbine noise annoyance [23-24]. A similar effect has been reported earlier with respect to annoyance from stationary sources (Miedema and Vos [25]),
- Economic benefits due to wind turbines: having economic benefit from the use of wind turbines is associated with decreased wind turbine noise annoyance (Van den Berg et al. [24]),
- Rural living area: Many wind turbines are installed in less densely populated areas, often in flat terrains. It is hypothesized that residents in such areas are used to and prefer quietness more than residents of densely populated areas (Pedersen [26]). On the other hand, in the Dutch study (2007), the distinction between rural and built-up area was used as a sample-stratification variable, and it turned out that respondents living in a rural area with a main road within 500 m from the wind turbine(s) were less annoyed than respondents living in a built-up area. This unexpected effect was not replicated in the other European studies.

A general factor should be mentioned: The present wind turbine noise studies refer to situations where the turbines have been built not very long ago. Establishing a wind turbine or a wind turbine park usually means a considerable change in the environment for the inhabitants of the affected area. The change itself may be an annoying factor, and this may be one of the reasons why wind turbine noise annoyance generally is higher than transportation noise annoyance at comparable noise levels.

S34.8. Summary of the wind turbine analyses

The two publications included in the wind turbine noise annoyance analysis contain descriptions of a total of four individual studies (a total of 2,481 respondents). Although there are differences between studies with respect to the annoyance rating (e.g., spatial frame of reference and response scale) and noise descriptor, we performed comparisons between reported exposure/response functions for % Highly Annoyed, increase of %HA with 5 dB level increase, and exposure/response correlations. The two comparisons based on % Highly Annoyed (ERFs and increase of %HA with level increase) led to inconsistent results and a low quality of evidence. In contrast, the formal meta-analysis based on raw score correlations showed a moderate quality of evidence (summary correlation r = 0.278; p = 0.001; 95% CI = 0.11 – 0.430). It is evident that the level of wind turbine sounds is systematically related to noise annoyance, even at levels below 40 dB L_{den}, but the exposure-response relations between noise levels and %HA is subject to inconsistency between studies.

S35. Combined noise effects

As stated by Taylor ([33], p. 123), "many residential communities are exposed to environmental noise from a mixture of sources." According to a representative survey in Germany, about 22% of the residents are annoyed by two noise sources in the vicinity of their homes, 11% by three sources, 6% by four sources, and 5% by five sources [34]. The major source component of the combinations is road traffic noise. The two major research questions with respect to annoyance in combined noise situations are:

1. How is the "total annoyance" judgment in situations involving at least two different noise sources related to the acoustic properties of the source combination?
2. How are the "separate annoyance" judgments with respect to each involved source modified by the acoustic properties of the source combination?

In this review, we will consider the "total annoyance" (TA) question only, because (a) it seems to be somewhat more relevant for health protection issues, and because (b) there are at least five studies available which can be compared, while a comparison between studies with respect to the second question lacks comparable data.

On the exposure side, the acoustic description of noise source combinations usually follows the energy summation principle – at least for administrative purposes, i.e., the estimated annual sound energy of each source is summed up and transformed to annual noise levels (e.g., $L_{Aeq,24h}$ or L_{den}). The Dutch Noise Annoyance Law weights the individual noise levels of source combinations by means of "annoyance equivalents" (e.g., Miedema [35]; see also [36]). This seems to be a plausible approach; however, it has not been tested in field situations, yet, and Miedema [35] questions whether this would ever be possible.

On the response side, study participants are asked to rate their "total annoyance" with respect to the "combined noise". Guski [37] listed five assumptions made in order to interpret the "total annoyance" judgment as a combination of the separate annoyance judgments. For instance, authors assume that the frame of reference for the total as well as for the separate annoyance judgments is the same. This may be questionable in cases when one source operates mainly at night and the other during the day – as sometimes is the case with road traffic (mostly daytime) and freight railway traffic (mostly nighttime). In this case, authors assume that respondents are able to combine and evaluate daytime and nighttime effects from different sources into a combined "total annoyance" judgment. Berglund and Nilsson [38] and Lercher [39] discuss similar theoretical aspects.

In addition, in a methodological study including psychology students as participants, Hatfield et al. [40, p. 922] observed that the question format may have an influence on responses with respect to "total annoyance": "The difference between self-reported reaction to noise pairs and the summed self-reported reaction to the single component noises was lower when participants were instructed to consider combined noises 'when they occur together', compared to when they were given no instruction (which did not differ from when participants were instructed to consider combined noises 'whether or not they occur at the same time')".

We included five field studies on noise source combinations, contained in four publications. All studies include road traffic noise; two of the studies combine road and railway noise, two combine road and aircraft noise, and one combines road and industrial noise. The total dataset includes 1,949 respondents (Table S19).

The exact annoyance question is only partially included in the publications. However, all studies included asked for ratings of "global", "total" or "combined" annoyance. Except for the French study, all studies used the ICBEN/ISO format of response scale (5-point verbal scale, partially augmented by the 11-point numeric scale). In three of the studies, "highly annoyed" is defined as ≥ 73% of the length of annoyance scale, in two others as ≥ 60% of the scale.

Table S19. Noise combination studies included

**Publication** (see S3 for references)	**Location**	**Year data**	**Sample type**	**Sample size**	**Noise level descriptors combined**	**Noise level range combined**	**Annoyance Scale**	**Remarks**	**Study Quality Rating**
Champelovier et al. 2003 (road + rail)	France	1997-1998	61 sites all over France	673	$L_{Aeq,24h}$	49-79	11-point ICBEN + 4-p verbal scale (inside) HA≥73 - 7%	Combined road + rail data used	19
Lercher et al. 2007 (road + rail)	Inn valley, Austria	2006	Stratified	49	L_{den}	35-80	5-point ICBEN scale HA≥60%	Combined road + rail data used	19
Nguyen et al. 2012 (road + aircraft)	Ho Chi Minh, Vietnam	2008	8 sites under flight path + 2 control sites	599	$L_{Aeq,24h}$ L_{den}	69-77	5-point + 11-point ICBEN scale HA≥73%	Combined road + aircraft data used	17
Nguyen et al. 2012 (road + aircraft)	Hanoi, Vietnam	2009	7 sites under flight path + 2 control sites	529	$L_{Aeq,24h}$ L_{den}	69-78	5-point + 11-point ICBEN scale HA≥73%	Combined road + aircraft data used	17
Pierrette et al. 2012 (road + industry)	small town near Lyon, France	2008 (?)	stratified	99	L_{den} L_{day} $L_{evening}$ L_{night}	43-70	5-point verbal scale + 11-point numerical scale (HA≥60%, not used here)	Combined road + industry data used	20

S35.1. Combined noise effects 1: Exposure-response relation

Since there are three different source combinations represented in our sample of five studies, and the maximum number of studies using the same combination is two, there is no chance to construct a reliable exposure-response relation for any of the three combinations. In addition, studies in the history of combined noise annoyance from Bottom [41] and Nguyen et al. [42] have shown that the relation between total annoyance and combined noise levels may be quite different, depending on the relations between noise levels of the two combined sources. This means that a single exposure-response relation that fits to all possible combinations of noise level relations between two sources cannot be established. Therefore, we refrained from combining any data in order to estimate a new global exposure-response relation. Instead, as an example, we reproduce a figure by Champelovier et al. ([43], p.103), depicting the relation between "total annoyance" and noise levels from road, rail, and combined noise (Figure S18). This example used the case of non-dominance, i.e., this graph uses only data from residents exposed to equal levels of road and rail traffic noise.

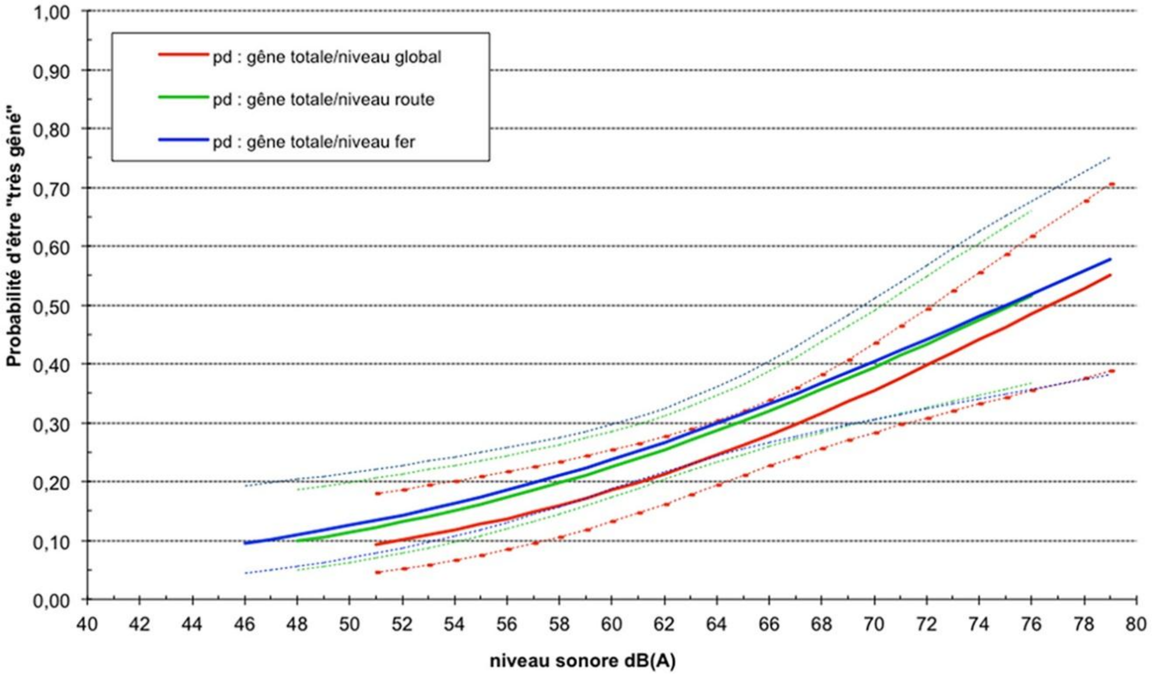

Figure S18. Relation between noise levels and "total annoyance" in case of equal noise levels from road and rail traffic noise. Reproduction of Fig. 39 from Champelovier et al. ([43], p 103). Legend: X-axis: $L_{Aeq,24h}$; Y-axis: probability of being "highly annoyed"; red curve: "total annoyance" vs. combined noise level; green curve: "total annoyance" vs. road traffic noise level; blue curve: "total annoyance" vs. railway noise level.

It has often been shown that the "total annoyance judgment" (TAJ) is somewhat lower than the maximum "specific annoyance judgments" (SAJ) in case of equal noise levels. This has raised many questions and different perceptual models of noise combinations (for an overview, see e.g., Pierrette et al. [44] or Nguyen [42]). Some of the mentioned models propose a masking effect of one source with respect to the other, and a general explanation is the assumption of different reference frames for the TAJ vs. SAJ.

In case of unequal noise levels of the source combination, a "dominance effect" is often observed, i.e., the TAJ is more in line with the SAJ from the louder source. It still may be that the TAJ is lower than the SAJ, but TAJ is closer to the SAJ of the louder source. This is true for the Champelovier-study [43] as well as for the Pierrette-study [44] on road plus industrial noise. With respect to the combination of dominant road traffic noise and non-dominant aircraft noise, Ngyuen et al. [42] observe that the "dominant source model" fits best to their TAJ data.

S35.2. Combined noise effects 2: Correlations with total annoyance judgments

Four of the five studies included provided Pearson correlations between $L_{Aeq,24h}$ or L_{den} levels of the combined sources, and for the remaining study, the ln(OR) from a logistic regression, estimated for a 10 dB difference, was converted to a correlation coefficient. In contrast to Pearson r, such a converted r depends on the absolute difference on the exposure scale (among other things), and it might be that the converted coefficient is a very coarse estimation of the true relation. A conversion of ln(OR) into r would produce a lower correlation, if – for example - the ln(OR) would have been estimated for a 5 dB difference instead for a 10 dB-difference.

The five (partially converted) correlations were (together with the respective n) subjected to a formal meta-analysis. The results are shown in Figure S19. All correlations are statistically significant ($p < 0.05$), and the summary correlation coefficient is 0.442 with a confidence interval from 0.111 to 0.684. This is a very large interval, and it seems evident that the interval is due to the difference between a group of moderate correlations (the two Road + Rail studies plus the Road + Industry study) and the group of high correlations (both Road + Aircraft studies from Vietnam). Accordingly, the heterogeneity test is statistically highly significant: Q = 462.591; df = 4; $p < 0.001$; $I^2 = 99.135$ – which means that 99 percent of the total variance is due to true variance between studies.

Study name	Subgroup within study	Statistics for each study				
		Correlation	Lower limit	Upper limit	Z-Value	p-Value
Lercher 2008	Tirol: Motorway, Road, Rail	0,116	0,078	0,154	5,916	0,000
Champelovier 2003	France: Road+Rail	0,249	0,176	0,319	6,554	0,000
Nguyen 2012	Hanoi: Aircraft+Road	0,728	0,685	0,766	21,203	0,000
Nguyen 2012	HoChiMinhCity: Aircraft+Road	0,686	0,641	0,726	20,496	0,000
Pierrette 2012	France: Road+Industry	0,224	0,027	0,403	2,229	0,026
		0,442	0,111	0,684	2,562	0,010

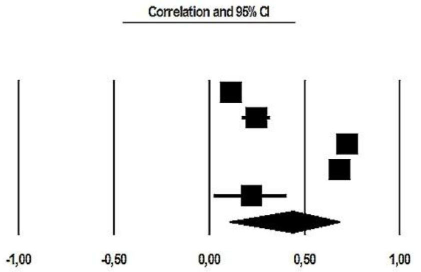

Figure S19. Meta-analysis of correlations between "total annoyance" and $L_{Aeq,24h}$ combined noise (the noise level of the two-sources combination). The right part of the graph contains a forest plot of the correlations and their respective 95% confidence intervals. The figures of the last row indicate the summary estimates.

The causes of the difference between the two groups with respect to correlations are unclear; they might relate to the special noise source-combination Road + Rail, to the considerable difference in noise levels between aircraft (high) and road traffic noise (low) reported by Nguyen et al. [42], or to other aspects unknown to us. We took this difference as an indication of the risks involved in comparing different studies on noise source combinations, and repeated the correlational analysis with three noise-combination studies, excluding the Road + Air studies. It turned out that both the summary correlation (r = 0.1, 95% CI = 0.077-0.293) and the heterogeneity (Q = 10.472; df = 2; p = 0.005) are considerably lower. However, the heterogeneity between studies is still statistically significant, and about 81% of the total variance is due to true variance between studies ($I^2 = 80.901$).

S35.3. Combined noise effects 3: Odds Ratios referring to the %HA increase per 10 dB level increase

In the meta-analysis of the preceding section (S35.2), the effect size ln(OR) for one of the studies was converted to r, and the meta-analysis was based on r as effect size for all five studies. Instead of converting ln(OR) into r, it is possible to convert the effect size r into OR. The analysis presented in Figure S20 is based on the OR estimate from the Lercher study [39] and on the ORs from the other four studies, which were converted from r into OR. In other words: Figure S19 and Figure S20 both are based on the same data, but the results are presented in different effect size formats -I in Figure S19 in terms of effect size r and in Figure S20 in terms of effect size OR.

All studies in Figure S20 show ORs greater than 1; the OR for one study is statistically significant ($p < 0.05$), and the ORs for the four other studies are statistically highly significant ($p < 0.01$). The summary OR is 6.633 (95% CI = 1.706-25.785; $p = 0.006$). However, the two studies with Road + Aircraft noise combinations have considerably higher ORs as compared to the other tree combination studies, and the heterogeneity test is again statistically highly significant: $Q = 357.309$; $df = 4$; $p < 0.001$. $I^2 = 98.881$ – which means that almost 99 percent of the total variance is due to true variance between studies.

Study name	Subgroup within study	Statistics for each study				
		Odds ratio	Lower limit	Upper limit	Z-Value	p-Value
Lercher 2008	Tirol: Motorway, Road, Rail	1,528	1,327	1,760	5,889	0,000
Champelovier 2003	France: Road+Rail	2,541	1,911	3,379	6,416	0,000
Nguyen 2012	Hanoi: Aircraft+Road	47,096	29,964	74,023	16,697	0,000
Nguyen 2012	HoChiMinhCity: Aircraft+Road	30,450	20,411	45,428	16,737	0,000
Pierrette 2012	France: Road+Industry	2,298	1,092	4,839	2,191	0,028
		6,633	1,706	25,785	2,731	0,006

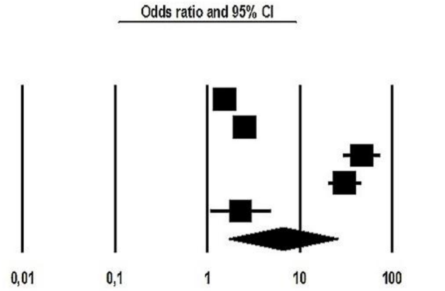

Figure S20. Odds Ratios and 95% confidence intervals from five studies, (based on modelled data) for the increase of the rate of "highly annoyed" persons with a 10 dB increase of $L_{Aeq,24h}$ combined noise. The right part of the graph contains a forest plot of the ORs and their respective 95% confidence intervals. The figures of the last row indicate the summary estimates.

With respect to the ORs, we observe a similar situation as in the case of the analyses based on correlations: the Road + Aircraft combination studies show considerably larger effects than the other combination studies, and the reasons for this difference are unclear in a similar way as discussed in section S35.2 above.

S35.4. Grading the evidence with respect to noise combinations

Study limitations of the observational studies reported here have been taken into account as far as possible. The **inconsistency of results** is remarkable, and may be attributed to the different noise source combinations (road and aircraft noise vs. road and railway noise vs. road and industry noise). On the other hand, all correlations are statistically highly significant greater than zero and all ORs are greater than one and statistically highly significant. With respect to the **indirectness of evidence**, we do not see relevant differences between the population and the sample of participants included in the studies**.** **Imprecision** may be seen as a problem, since there are two studies including less than 100 participants. With respect to **publication bias**, the small amount of studies does not allow for any conclusions.

In sum, we are confident in the evidence with respect to the direction of effects: all noise combinations show positive correlations with total annoyance judgments as well as ORs, which are greater than one and statistically highly significant. The quality of evidence with respect to correlations is seen as "high" (Table S20), and since the OR analysis presented here in Figure S19 is based on correlations (converted to OR), the same grading can be applied to ORs for an increase of %HA per 10 dB level increase. On the other hand, it should be noted that the respective effect size measures seem to be highly dependent on the type of noise source combination.

Table S20. GRADE summary of findings for the quality of evidence related to combined noise and degree of total annoyance. Health outcome based on correlations, five studies.

Domains	Criterion	Assessment	Grading
Start Level	Study design: cross-sectional = high qual.	High quality	High quality
1. Study Limitations	Quality of majority of studies (risk of bias)	High quality of majority of studies	No downgrade
2. Inconsistency	Conflicting results; high I2	High between study variance	Downgrade one level
3. Directness	Direct comparison; same PECO	Same PECO	No downgrade
4. Precision	Small sample sizes OR Low numbers of events (HA) OR Wide confidence intervals	Large study samples	No downgrade
5. Publication Bias	Funnel plot indicates	Not applicable	No downgrade
Overall Judgment			Moderate quality
6. Exposure-response	Statistically significant trend	All 5 studies show statistically significant exposure-response relations	Upgrade one level
7. Magnitude of effect	Weighted mean r > .5	Weighted mean r = .442	No upgrade
8. Confounding adjusted	Effect in spite of confounding working towards the nil	No adjustments	No upgrade
Overall Judgment			High quality

PECO = Population, Exposure, Comparator, Outcome. For explanations, see section S4.

S35.5. The influence of co-determinants in noise-combination studies

Besides the personal within-study factors mentioned earlier (e.g., noise sensitivity and coping capacity), there are several co-determinants within- and between-study factors which should be taken into account when analyzing noise annoyance from combined noise: Champelovier et al. [43] note as an important situational factor contributing to increased annoyance the short distance to the noise source (4-50m).

In contrast to the factors just mentioned, Pierrette et al. [44] underline in their Road + Industry combination study the contribution of fear of danger from industrial sites to annoyance. "Fear of danger" is often reported with respect to aircraft operations and is usually seen as a personal factor, but when it is shared by many residents in the vicinity of the same source, it may become a social factor, too [45]. According to Pierrette at al. [44], residents in the vicinity of industrial sites often mention the risk of accidents from the site (e.g., chemical poison in the air).

S35.6. Summary of the noise combination analyses

We considered the question how the (long-term) "total annoyance" judgment in situations involving at least two different noise sources is related to the long-term energetically summated noise levels of the combination of two noise sources. Five studies were available for comparison, all of which include road traffic noise; two of the studies combine road and railway noise, two studies combine road and aircraft noise, and one combines road and industrial noise. The total dataset consists of 1,949 respondents. Although the summary correlation between summated noise level and the judgment of "total annoyance" is statistically highly significant ($r = 0.442$, $p < 0.001$), and the 10-dB-increase of the

summated noise level shows a summary OR of 6.633 (95% CI = 1.706-25.785; p = 0.006), the variance between studies is largely unexplained. One plausible cause of between-study variance is the type of noise source combination: The Road + Aircraft combinations show larger effects on annoyance than any of the Road + Rail od Road + Industry combinations with respect to the two effect size measures used here: correlations between annoyance raw scores and noise level as well as the ORs referring to the %HA increase by a noise level increase of 10 dB.

It seems unwise to integrate different noise source combinations in a single analysis. However, there were not enough studies available for the meta-analysis of a single source combination. With respect to the weights given for the separate noise levels in future combination studies, our results point to the importance of the dominant source in terms of annoyance: since aircraft noise annoyance is generally higher than road and rail traffic annoyance at comparable noise levels, any combination of aircraft noise with another noise source will produce higher annoyance effects than any other combination examined here.

S36. Effects of noise from stationary sources

This section handles just one empirical study from one country as no other study showed up in the systematic literature search related to stationary noise sources. We did not have any data to test the assumption that the results may be similar in other countries.

Miedema and Vos [25] present data and exposure-response relations based on a field study (n=1,875) at 11 locations (two shunting yards, one seasonal industry, and eight other industries) in The Netherlands. Shunting yards are railway areas where trains or wagons are switched from one track to another. This activity is associated with loud impulsive sounds. The seasonal industry emits several times per day peak sounds from unloading trucks, lasting about 90 days per year, and the eight other industries are characterized by a mixture of continuous production or ventilation sounds and peak sounds by alarm bells, steam outlets, and other process sounds. Locations were selected with one or a few dominant noise sources and sufficient dwellings in the L_{den} level range 45–65 dB. The noise data of all locations were assessed and updated by various acoustical engineering companies. The respondents were selected according to a design stratified by level classes of 5 dB in the range of 45-65 dB L_{den}. The 10-minute telephone interviews were held in April and May 2001 and included the following topics: dwelling and surroundings; noise annoyance from various sources; noise annoyance from specific activities of the industry/shunting yard or from specific characteristics of the noises; changes in exposure; visibility of industry/shunting yard; annoyance from odor, vibration, concern about safety; demographical characteristics; relation with or use of the source; and noise sensitivity. The wording of the noise annoyance question was as follows: "If you are thinking of the past year, to which extent do you find the noise of (industrial area/shunting yard) annoying or not annoying?". The 11-point numerical response scale was labeled at the endpoints only (00 = "not at all annoying" … 10 = "very much annoying").

In the course of initial statistical analyses, it turned out that noise annoyance in the vicinity of the two shunting yards is higher than the annoyance found at the same L_{den} at other locations. At the location where most activities are restricted to one season, the annoyance is lowest. In the following analyses, three types of noise sources were distinguished: shunting yards, seasonal industry, and other industries. Different multivariate models were tested, and estimations of %A and %HA (among others) in the range of 45-65 dB L_{den} were presented for each of the three sources. %HA is defined in this study at a cutoff at 72% of the scale length, and %A is defined at a cutoff at 50% of the length. Some of the results are given here as a copy of Fig. 2 from the Miedema and Vos [25] paper – see Figure S21 below.

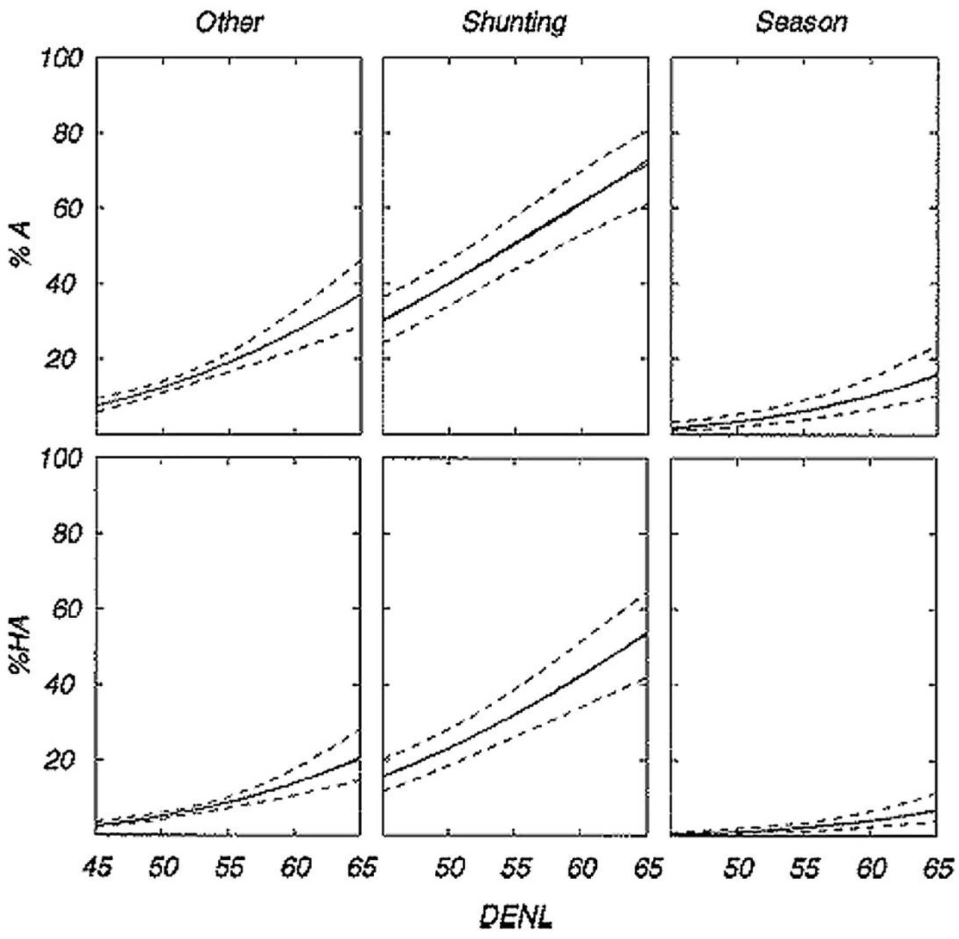

Figure S21. Exposure-response relations for %A (percent annoyed) and %HA (percent highly annoyed) and three types of noise from stationary sources. The noise level type DENL is equivalent to L_{den}. The graph is a partial reproduction of Fig. 2 in Miedema & Vos [25].

At the same (annual) L_{den}, the seasonal industry causes less annoyance than the other industries, while the other industries cause less annoyance than the shunting yards. It appears that the noise annoyance caused by shunting yards is influenced by shunting vibrations and noise from trains. The relatively low annoyance from the seasonal industry presumably is related to the presence of a relatively short noisy time period and a long quiet period. It should be noted that the results for shunting yards and seasonal industry are based on fewer data than the other industrial sources and may not be reproduced in other locations.

S37. Abbreviations and terms used

CMA: Comprehensive Meta-Analysis (a software developed by Borenstein and coworkers. We used V3 (2015)).

Confidence interval, 95% confidence interval (95% CI): Technically, this means that, if the experiment were repeated many times, 95 percent of the CIs would contain the true population mean. (http://www.psychologicalscience.org)

Funnel plot: Plot of the effect size (X-axis) vs. standard error (Y-axis). The plot is used to detect publication bias. “In the absence of publication bias, the studies will be distributed symmetrically about the mean effect size, since the sampling error is random. In the presence of publication bias the studies are expected to follow the model, with symmetry at the top, a few studies missing in the

middle, and more studies missing near the bottom. If the direction of the effect is toward the right (as in our example), then near the bottom of the plot we expect a gap on the left, where the nonsignificant studies would have been if we had been able to locate them" (Borenstein et al. [3], p. 283).

GRADE: Grading of Recommendations Assessment, Development and Evaluation, developed by a group of experts, and described by Guyatt et al. [1, 46].

HA: Highly Annoyed (Survey respondents choosing the higher points on a standardized annoyance rating scale).

Heterogeneity: relates to the dispersion of the effect-size estimates between studies. Borenstein et al. ([3], p. 106) use the term "heterogeneity" to mean heterogeneity in true effects only.

I^2: The proportion of the observed variance which reflects real differences in effect size. I-square relates to the ratio of true heterogeneity to total variance across the observed effect estimates (cf. [3], p. 117).

ICBEN: International Committee on the Biological Effects of Noise.

ISO: International Standards Organization.

L: Acoustic descriptor of sound/noise level.

L_{Aeq}: Acoustic descriptor of energy-equivalent sound pressure levels, frequency-weighted according to the "A" filter, and related to a certain time. For instance, $L_{Aeq,24h}$ comprises the 24 hours of a day, $L_{Aeq,16h}$ comprises 16 hours of a day. There is no specific weighting of certain daytime or nighttime situations. Other time specifications are used, too.

L_{day}: The energy-equivalent sound pressure level over one day (12 or 16 hours). Often used as a yearly average.

L_{den}: The energy-equivalent sound pressure level over 24 hours, often used as a yearly average. In this compound indicator the evening value gets a penalty of 5 dB and the night value of 10 dB.

L_{night}: The energy-equivalent sound pressure level over one night (8 hours). Often used as a yearly average.

PRISMA: Preferred Reporting Items for Systematic reviews and Meta-Analyses, described by Moher et al., 2009.

Q: relates to the homogeneity or heterogeneity of effect size estimates between the studies. Q is a statistic to test the null hypothesis that all studies share a common effect size. Under the null hypothesis Q will follow a central chi-squared distribution with degrees of freedom equal to $k-1$. (cf. [3], p. 112).

Tolerance interval: A tolerance interval around a pooled estimate may be used to judge if a new (single) study is within the interval, and therefore may be expected to come from the same population of studies.

WHO: World Health Organization.

References

1. Guyatt, G.H., Oxman, A.D., Kunz, R., Vist, G.E., Falck-Ytter, Y. & Schünemann, H.J. What is "quality of evidence" and why is it important to clinicians? *BMJ,* ***2008****, 336*(7651), 995-998. doi: 10.1136/bmj.39490.551019.BE
2. Babisch, W., Houthuijs, D., Pershagen, G., Cadum, E., Katsouyanni, K., Velonakis, M., et al. for the HYENA-team. Annoyance due to aircraft noise has increased over the years - results of the HYENA study. *Environment International****, 2009****, 35, 1169-1176.*
3. Borenstein, M., Hedges, L. V., Higgins, J. P. T. & Rothstein, H. *Introduction to Meta-Analysis*. Chichester: Wiley, 2009.
4. Rothstein, H. R. Publication bias as a threat to the validity of meta-analytic results. *Journal of Experimental Criminology****, 2008****, 4(61-81).*
5. Higgins, J. P. T. & Thompson, S. G. Quantifying heterogeneity in a meta-analysis. *Statist. Med.,* ***2002****, 21, 1539-1558.*
6. Higgins, J. P. T. Commentary: Heterogeneity in meta-analysis should be expected and appropriately quantified. *International Journal of Epidemiology,* ***2008****, 37, 1158-1160. 2008, 37, 1158-1160.*
7. Janssen, S. A., Vos, H., Van Kempen, E., Breugelmans, O. & Miedema, H. M. E. Trends in aircraft noise annoyance: The role of study and sample characteristics. *Journal of the Acoustical Society of America,* ***2011,*** *129(4), 1953-1962.*
8. Janssen, S. A. & Guski, R. Aircraft noise annoyance. In S. A. Stansfeld, B. Berglund, S. Kephalopoulos & M. Paviotti (Eds.), *Evidence Review on Aircraft Noise and Health*. Bonn (D): Directorate General Joint Research Center and Directorate General for Environment, European Union. (in press).
9. Cohen, J. *Statistical power analysis for the behavioral sciences*. Hillsdale (USA, NJ): Lawrence Earlbaum Associates, 1988.
10. Öhrström, E., Skånberg, A., Svensson, H. & Gidlöf-Gunnarsson, A. Effects of road traffic noise and the benefit of access to quietness. *Journal of Sound and Vibration,* ***2006****, 295, (1-2), 40-59.*
11. De Kluizenaar, Y., Janssen, S. A., Vos, H., Salomons, E. M., Zhou, H. & van den Berg, F. Road traffic noise and annoyance: a quantification of the effect of quiet side exposure at dwellings. *Int J Environ Res Public Health,* ***2013****, 10(6), 2258-2270. doi: 10.3390/ijerph10062258.*
12. Miedema, H. M. E. Response functions for environmental noise in residential areas (NIPG-Publikatienummer 92.021, pp. 109). Leiden, NL: Nederlands Instituut voor Praeventieve Gezondheidszorg, 1993.
13. Lercher, P., Bockstael, A., Dekoninck, L., Coensel, B. D. & Botteldooren, D. Can noise from a main road be more annoying than from a highway? An environmental health and soundscape approach. Paper presented at the Inter-Noise 2013, Innsbruck (A).
14. Wunderli, J.-M., Pieren, R., Habermacher, M., Vienneau, D., Cajochen, C., Probst-Hensch, N., Röösli, M. & Brink, M. Intermittency Ratio - A metric reflecting short-term temporal variations of transportation noise exposure. *Journal Of Exposure Science And Environmental Epidemiology* (advance online publication, 9 September **2015**; doi:10.1038/jes.2015.56), 1-11.
15. Yano, T., Morihara, T. & Sato, T. Community response to Shinkansen noise and vibration: a survey in areas along the Sanyo Shinkansen Line. Paper presented at the Forum Acusticum, Budapest (H) 2005.
16. Gidlöf-Gunnarsson, A., Ögren, M., Jerson, T. & Öhrström, E. Railway noise annoyance and the importance of number of trains, ground vibration, and building situational factors. *Noise & Health****, 2012****, 14(59), 190-201.*
17. Schomer, P., Mestre, V., Fidell, S., Berry, B., Gjestland, T., Vallet, M. & Reid, T. Role of community tolerance level (CTL) in predicting the prevalence of the annoyance of road and rail noise. *Journal of the Acoustical Society of America****, 2012****, 131(4), 2772-2786.*
18. Sato, T., Yano, T. & Morihara, T. Effects of Situational Variables on Community Response to Shinkansen Noise: A Survey in Kyushu, Japan. Paper presented at the Inter-Noise 2004, Prague (CZ).
19. Lim, C., Kim, J., Hong, J. & Lee, S. The relationship between railway noise and community annoyance in Korea. *The Journal of the Acoustical Society of America,* ***2006****, 120(4), 2037-2042. doi: doi:http://dx.doi.org/10.1121/1.2266539.*
20. Sato, T., Yano, T., Björkman, M. & Rylander, R. Comparison of community response to road traffic noise in Japan and Sweden - Part I: Outline of surveys and dose response relationships. *Journal of Sound and Vibration,* ***2002****, 250, 161-167.*

21. Zeichart, K., Sinz, A., Schweiger, M., Kilcher, H. & Herrmann, W. Untersuchung zur Lästigkeit von Reise- und Güterzügen. Bericht über ein interdisziplinäres Forschungsvorhaben im Auftrag der Deutschen Bahn AG. Abschlussbericht. München: Studiengemeinschaft Schienenverkehr (SGS), 2001.
22. Schreckenberg, D. Exposure-response relationship for railway noise annoyance in the middle Rhine Valley. Paper presented at the Inter-Noise 2013, Innsbruck (A).
23. Kuwano, S., Yano, T., Kageyama, T., Sueoka, S. & Tachibanae, H. Social survey on wind turbine noise in Japan. *Noise Control Engineering Journal,* ***2014****, 62*(6), 503-520.
24. Van den Berg, F., Pedersen, E., Bouma, J. & Bakker, R. WINDFARM perception. Visual and acoustic impact of wind turbine farms on residents. Final report (Vol. FP6-2005-Science-and-Society-20, Project no. 044628). Groningen (NL): University of Groningen 2008.
25. Miedema, H. M. E. & Vos, H. Noise annoyance from stationary sources: Relationships with exposure metric day evening night level (DENL) and their confidence intervals. *Journal of the Acoustical Society of America,* ***2004,*** *116, 334-343.*
26. Janssen, S. A., Vos, H., Eisses, A. R. & Pedersen, E. A comparison between exposure-response relationships for wind turbine annoyance and annoyance due to other noise sources. *Journal of the Acoustical Society of America,* ***2011****, 130(6), 3746-3753*. doi: 10.1121/1.3653984.
27. Wirth, K., Brink, M. & Schierz, C. Lärmstudie 2000. Schlussbericht zur 2. Befragungsstudie vom August 2003 (Vol. http://e-collection.library.ethz.ch/view/eth:29188). Zürich (CH): ETH Zürich, Zentrum für Organisations- und Arbeitswissenschaften, 2006.
28. Terrel, C. C. Table for converting the point biserial to the biserial. *Educational and Psychological Measurement,* ***1982****, 42, 982-986.*
29. Nguyen, T., Yano, T. & Morihara, T. A method to compare the prevalence of annoyance measured with different scales. Paper presented at the InterNoise 2013, Innsbruck (A).
30. Fleiss, J. L. & Berlin, J. A. Effect Sizes for Dichotomous Data. In H. Cooper, L. V. Hedges & J. C. Valentine (Eds.), The Handbook of Research Synthesis and Meta-Analysis, 2nd ed., 2009 (pp. 237-253). New York: Russell Sage Foundation.
31. Sweeting, M. J., Sutton, A. J. & Lambert, P. C. What to add to nothing? Use and avoidance of continuity corrections in meta-analysis of sparse data. *Statistics in Medicine, 23(9),* ***2004****, 1351-1375.* doi: 10.1002/sim.1761.
32. Pedersen, E. Perception and annoyance due to wind turbine noise--a dose-response relationship. (Ph.D. thesis), Göteborgs Universitet, Göteborg (S), 2007.
33. Taylor, S. M. A comparison of models to predict annoyance reactions to noise from mixed sources. *Journal of Sound and Vibration,* ***1982****, 81, 123-138.*
34. Bundesministerium für Umwelt, Naturschutz, Bau und Reaktorsicherheit (BMUB) & Umweltbundesamt. *Umweltbewusstsein in Deutschland 2014. Ergebnisse einer repräsentativen Bevölkerungsumfrage*. Berlin / Dessau-Rosslau: BMUB / UBA, 2014.
35. Miedema, H. M. E. Relationship between exposure to multiple noise sources and noise annoyance. *Journal of the Acoustical Society of America,* ***2004****, 116*, 949-957.
36. Verein Deutscher Ingenieure. VDI 3722 Blatt 2, Bewertung von Verkehrsgeräuschen beim Einwirken mehrerer Quellen. Berlin: Beuth, 2012.
37. Guski, R. Interference of activities and annoyance by noise from different sources: Some new lessons from old data. In A. Schick & M. Klatte (Eds.), Contributions to Psychological Acoustics. Results of the 7th Oldenburg Symposium on Psychological Acoustics, 1997 (pp. 239-258). Oldenburg: BIS Oldenburg.
38. Berglund, B. & Nilsson, M. E. Loudness of Combined Noises Derived from Singular and Concurrent Community Noises. Paper presented at the ICA-1998, Seattle (USA).
39. Lercher, P. Combined Noise Exposure at Home. In J. O. Nriagu (Ed.), Encyclopedia of Environmental Health, 2011 (pp. 764–777). Amsterdam / London Elsevier.
40. Hatfield, J., van Kamp, I. & Job, R. F. S. (2006). Clarifying "Soundscape": Effects of Question Format on Reaction to Noise from Combined Sources. *Acta Acustica united with Acustica, 92*(6), 922-928.
41. Bottom, C. G. A social survey into annoyance caused by the interaction of aircraft noise and traffic noise. *Journal of Sound and Vibration,* ***1971****, 19, 473-476.*
42. Nguyen, T., Yano, T., Nguyen, H., Nishimura, T., Sato, T. & Morihara, T. Community response to aircraft noise around three airports in Vietnam. Paper presented at the Acoustics 2012, Hong Kong.
43. Champelovier, P., Cremezi-Charlet, C. & Lambert, J. Evaluation de la gêne due à l'exposition combinée aux bruits routier et ferroviaire (Report 242). Lyon: INRETS, 2003.

44. Pierrette, M., Marquis-Favre, C., Morel, J., Rioux, L., Vallet, M., Viollon, S. & Moch, A. Noise annoyance from industrial and road traffic combined noises: A survey and a total annoyance model comparison. *Journal of Environmental Psychology*, **2012**, *32(2), 178-186.*
45. Flindell, I. H. & Stallen, P. J. Non-acoustical factors in environmental noise. *Noise & Health*, **1999**, *3, 11-16.*
46. Guyatt, G. H., Oxman, A. D., Vist, G. E., Kunz, R., Falck-Ytter, Y., Alonso-Coello, P. & Schünemann, H. J. (2008). GRADE: an emerging consensus on rating quality of evidence and strength of recommendations. *BMJ, 2008*(336:924).

© 2017 by the authors. Submitted for possible open access publication under the terms and conditions of the Creative Commons Attribution (CC-BY) license (http://creativecommons.org/licenses/by/4.0/).
